# Propofol infusion syndrome: a structured review of experimental studies and 153 published case reports

**DOI:** 10.1186/s13054-015-1112-5

**Published:** 2015-11-12

**Authors:** Adéla Krajčová, Petr Waldauf, Michal Anděl, František Duška

**Affiliations:** Laboratory for Metabolism and Bioenergetics, Third Faculty of Medicine, Charles University in Prague, Prague, Czech Republic; Centre for Research on Diabetes, Metabolism and Nutrition, Third Faculty of Medicine, Charles University in Prague, Prague, Czech Republic; Department of Anaesthesiology and Intensive Care, Third Faculty of Medicine, Charles University in Prague, Prague, Czech Republic; Adult Intensive Care Unit, Nottingham University Hospitals NHS Trust, Nottingham, UK

## Abstract

**Introduction:**

Propofol infusion syndrome (PRIS) is a rare, but potentially lethal adverse effect of a commonly used drug. We aimed to review and correlate experimental and clinical data about this syndrome.

**Methods:**

We searched for all case reports published between 1990 and 2014 and for all experimental studies on PRIS pathophysiology. We analysed the relationship between signs of PRIS and the rate and duration of propofol infusion causing PRIS. By multivariate logistic regression we looked at the risk factors for mortality.

**Results:**

Knowledge about PRIS keeps evolving. Compared to earlier case reports in the literature, recently published cases describe older patients developing PRIS at lower doses of propofol, in whom arrhythmia, hypertriglyceridaemia and fever are less frequently seen, with survival more likely. We found that propofol infusion rate and duration, the presence of traumatic brain injury and fever are factors independently associated with mortality in reported cases of PRIS (area under receiver operator curve = 0.85). Similar patterns of exposure to propofol (in terms of time and concentration) are reported in clinical cases and experimental models of PRIS. Cardiac failure and metabolic acidosis occur early in a dose-dependent manner, while arrhythmia, other electrocardiographic changes and rhabdomyolysis appear more frequently after prolonged propofol infusions, irrespective of dose.

**Conclusion:**

PRIS can develop with propofol infusion <4 mg/kg per hour and its diagnosis may be challenging as some of its typical features (hypertriglyceridaemia, fever, hepatomegaly, heart failure) are often (>95 %) missing and others (arrhythmia, electrocardiographic changes) occur late.

**Electronic supplementary material:**

The online version of this article (doi:10.1186/s13054-015-1112-5) contains supplementary material, which is available to authorized users.

## Introduction

After its launch in 1986, propofol became one of the most popular drugs used for induction and maintenance of anaesthesia and for sedation in intensive care units (ICUs). It is estimated that 1 billion 20-ml equivalents is sold worldwide every year and this amount continues to increase [1]. Over the last decade the average price of a 20-ml equivalent has dropped from US$5 to less than US$3 [2]. Increased affordability of propofol together with its potential to reduce mortality [3] and the length-of-stay [4] of the critically ill resulted in a steep rise in the use of propofol for non-procedural sedation of patients in ICUs.

Propofol infusion syndrome (PRIS) is a rare but potentially lethal side effect of propofol. There is no widely accepted definition, but in most cases various combinations of the following are described: unexplained metabolic acidosis, rhabdomyolysis, hyperkalaemia, hepatomegaly, renal failure, hyperlipidaemia, arrhythmia, Brugada-type electrocardiograph (ECG; elevated ST-segment and coved T-wave) and rapidly progressive cardiac failure. The first cases were described in children in the early 1990s [5–11], and cases in adults were reported soon after [12–15]. PRIS did not gain significant attention until Cremer et al. published a landmark paper in *The Lancet* [16] of seven cases of PRIS in a neurosurgical ICU and they were the first to highlight that the cumulative dose of propofol was the main risk factor for its development. A few months later the US Food and Drug Administration (FDA) warned against the use of propofol for prolonged sedation in paediatric populations [17]. In 2006 the FDA updated the labelling information and limited the maximum dose of propofol recommended for sedation to 4 mg/kg per hour [18]. At the same time European regulatory authorities suggested that patients be monitored for metabolic acidosis, hyperkalaemia, rhabdomyolysis or an elevated creatine kinase level, and/or signs of heart failure. If any of these conditions developed, they recommended a dosage reduction or discontinuation of propofol [19, 20]. Despite increasing public awareness and regulatory measures in place, PRIS continues to occur and kill. A European database of suspected adverse drug reactions run by The European Medicines Agency registered a total of 394 cases of PRIS between December 2001 and March 2015, of which 137 (35 %) were fatal [21].

In this paper we analysed data from 153 patients from case reports and case series published between 1990 and 2014. We also reviewed experimental (animal and ex vivo) studies on mechanisms of PRIS. The primary aim of the study was to determine the relationship of propofol exposure in patients identified with PRIS with clinical and laboratory outcomes. We used multiple regression analysis to analyse factors associated with fatal outcome of PRIS. Secondary aims were to find a link between the clinical presentations of PRIS with proposed cellular mechanisms and to describe trends in the reporting of PRIS over time.

## Methods

In order to find all available publications on the topic, we used a three-stage search technique and minimised language limitations. Consent for the study was not required, as the reports had been previously published. Firstly, we performed a search on the PubMed database using the keywords “propofol-infusion syndrome” which yielded 275 articles. With help from a medical librarian we obtained full texts of all original case reports or case series of patients where the authors felt there was, or may have been, PRIS. We did not exclude any language and obtained articles in English, German, Spanish, Chinese, Danish and Norwegian. We used Google Translate to extract the data from articles in Scandinavian languages and the English abstract only for one article in Chinese. In the second stage, we searched references of these articles for further articles fulfilling the eligibility criteria described above but not identified during our original search. Finally, we also included reports of unexpected metabolic acidosis and arrhythmia in patients on propofol published prior to the 1998 definition of PRIS [22]. This resulted in 94 manuscripts (see Additional file 1 for the full list of references) reporting on 153 patients with suspected PRIS.

In the full texts of these papers we specifically looked at patients’ demographic characteristics (sex and age), underlying disease (categorized as traumatic brain injury (TBI), respiratory infection, status epilepticus, other neurological cause or others), average propofol dose (mg/kg per hour), duration of propofol administration (hours), symptoms of PRIS classified as a binary variable (1 = present, 0 = absent or not reported), unexplained metabolic acidosis, arrhythmias, other ECG changes, cardiac failure, acute respiratory distress syndrome, hypotension, acute kidney injury (AKI; any report of oligoanuria or increased creatinine), rhabdomyolysis (or elevated creatine kinase or myoglobin), lipaemia (or increased plasma triglycerides), liver damage and unexplained fever. We consider a sign as being present if the original case report authors described it as a sign of PRIS—in most case reports this causation is suspected if a sign occurred or worsened after propofol administration and disappeared or improved after cessation of propofol administration. We did not analyse concomitant medication (vasopressors and steroids) as these data were missing in >50 % of published cases. In each case, the outcome was defined as “survived” (if the patient was reported to recover from PRIS) or “died”, regardless of the report authors’ opinion on the cause of death. These values were extracted by one of the authors (AK) and independently checked by another (FD). In order to describe the change in case reports over time, we arbitrarily divided case reports into three groups (n = 27, 53 and 72, respectively) demarcated by the years of publication: 2001 (the year of banning prolonged propofol infusion in children and the acceptance of the existence of PRIS in adults) and 2006 (the year when the safety limit of 4 mg/kg per hour was recommended).

### Statistical analysis

In order to describe trends over time, we used simple linear regression. Multiple logistic regression analysis of risk factors for mortality was performed in three steps. Firstly, we performed univariate analysis of each independent factor that might influence mortality. Continuous variables (age, average dose and duration) were categorised into quintiles. Variables without an associated influence on mortality at *p* < 0.1 were then excluded. For the remaining variables we performed correlation analysis and, if a significant (*p* < 0.05) correlation was found between the two variables, only the one with stronger prediction in univariate analysis was entered into the final model. Finally, we performed multiple logistical regression analysis to calculate the influence of every single independent variable on mortality. The receiver operator curve area under the curve of the final model was calculated. Values are reported as odds ratios (95 % confidence intervals). All calculations were performed using Stata Software (version 13.1; Stata Corp. Ltd., USA), and *p* values <0.05 were considered significant. Detailed step-by-step results of logistic regression analysis are available in Additional file 1.

## Results

### Change of features of PRIS over time

As demonstrated in Additional file 2: Figure S1, PRIS continues to be reported in the literature in both adult and paediatric populations without a clear trend to a reduction. Among 153 cases of PRIS published between 1986 and 2015, 78 (51 %) had a fatal outcome. Fatality rates in reported cases decreased over time from 74 % (20–27 %) before 2001, to 64 % (34–53 %) between 2001 and 2006, and to 32 % (23–72 %) in cases reported after 2006. In 138 cases (90 %) patients developed PRIS as a complication of non-procedural sedation in ICUs and in 15 cases (10 %) as a complication of the use of propofol during anaesthesia.

The duration of infusion leading to PRIS in reported cases increased over time and the mean propofol infusion rate decreased, leading to an overall decrease in the cumulative propofol dose leading to PRIS (see Additional file 3). Mean age of patients in reported cases increased over time, and this trend is apparent even if paediatric cases are excluded from the analysis (data not shown).

There was no change in the underlying disease of patients with PRIS over time (data not shown), but the incidence of some reported signs of PRIS did change (see Additional file 4). Arrhythmia, other ECG changes, hypertriglyceridaemia and fever are all reported with decreasing frequency. Metabolic acidosis remained the most common symptom of PRIS with a constant incidence at around 77 % in reported cases. See Additional file 1: Table S1 for detailed extracted data from all case reports.

### Factors influencing mortality in published cases of PRIS

Demographic characteristics, underlying disease, frequency of symptoms and their influence on mortality in univariate analysis are summarized in Table 1. In order to make sure that only variables independent of each other were entered into the logistical regression model, we performed a correlation analysis. There were significant correlations between arrhythmias and other ECG changes (R = 0.87, *p* < 0.001) and as expected between the cumulative dose and both the infusion duration (R = 0.47, *p* < 0.001) and average infusion rate (R = 0.38, *p* < 0.001). We thus eliminated arrhythmia and cumulative dose from the analysis. Hepatomegaly and fatty liver were also eliminated, as these findings are more likely reported in cases which included autopsy findings (i.e. those with fatal outcome). We also found negative correlations between age >55 and both average dose (R = –0.34, *p* < 0.001) and arrhythmia (R = –0.39, *p* < 0.001). Multivariate logistical regression performed on 128 cases with complete datasets identified higher dose, longer duration of infusion, development of fever and the presence of TBI as factors associated with increased mortality of PRIS, whilst all other factors were eliminated from the model (see Additional files 5 and 6). Detailed step-by-step results of regression analysis can be found in Additional file 1.

**Table 1:** Characteristics of reported cases of propofol infusion syndrome and univariate mortality risk

	Frequency, n (%)	Unadjusted mortality risk		
		Odds ratio	*P*	95 % CI
Demography				
Elderly (age >55 years)	29 (19 %)	0.11	<0.001	0.04–0.34
Child (age <18 years)	56 (36 %)	1.4	0.318	0.72–2.7
Male sex	80 (60 %)^a^	0.85	0.646	0.42–1.7
Underlying disease				
Respiratory infection	22 (14 %)	2.28	0.093	0.87–6.0
Traumatic brain injury	43 (28 %)	4.7	<0.001	2.1–10.5
Status epilepticus	30 (20 %)	0.67	0.331	0.3–1.5
Non-trauma neurological dg. (cerebral vascular malformation, aneurysm, sinus thrombosis, brain tumor etc.)	15 (10 %)	0.60	0.36	0.2–1.8
Other	43 (28 %)	0.25	<0.001	0.12–0.54
Propofol dose and duration of infusion				
Average dose (continuous variable)	128 (84 %)^b^	1.12	0.08	0.99–1.28
Average dose above 5 mg/kg per hour	76 (59 %)	4.20	<0.001	1.98–8.9
Duration (continuous variable)	150 (98 %)^c^	1.005	0.109	0.999–1.012
Duration <20 hours	30 (20 %)	1.00	N/A	N/A
Duration 20–60 hours versus <20 hours	35 (23 %)	43.5	<0.001	9.9–191.4
Duration >60 hours versus <20 hours	85 (57 %)	10.6	<0.001	3.0–37.7
Cumulative dose (continuous variable)	128 (84 %)^b^	1.001	0.079	0.999–1.002
Cumulative dose above 360 mg/kg	76 (59 %)	2.74	0.007	1.33–5.68
Symptoms				
Metabolic acidosis	117 (77 %)	2.48	0.024	1.13–5.45
Brugada-like or ischaemic ECG	102 (67 %)	3.29	0.001	1.61–6.73
Arrhythmia	101 (66 %)	4.6	<0.001	2.19–9.55
Rhabdomyolysis	85 (56 %)	1.44	0.27	0.76–2.73
Acute kidney injury	60 (39 %)	1.02	0.944	0.53–1.96
Hypotension	45 (30 %)	2.15	0.037	1.05–4.4
Hyperkalaemia	37 (24 %)	2.86	0.009	1.29–6.34
Hypertriglyceridaemia	37 (24 %)	1.79	0.132	0.84–3.83
Cardiac failure	35 (23 %)	1.17	0.4	0.55–2.49
Fever	29 (19 %)	8.25	<0.001	2.71–25.15
Abnormal liver function test	20 (13 %)	0.94	0.899	0.37–2.4
Discolouration of urine	16 (11 %)	2.27	0.148	0.75–6.87
Hepatomegaly or fatty liver	16 (11 %)	4.73	0.019	1.29–17.36
Pulmonary oedema	4 (3 %)	N/A	N/A	N/A

^a^Missing sex in 19 (12 %) patients

^b^Missing value for propofol dose in 25 (16 %) patients

^c^Missing value for duration in 3 (2 %) patients

*CI* Confidence interval, *ECG* Electrocardiograph, *N/A* Not available

### Influence of propofol infusion rate and duration on signs of PRIS

Additional file 7: Figure S5 demonstrates that actual propofol infusion rate is a factor significantly influencing the frequency of fever (which occurs in <5 % of patients with an infusion rate <4 mg/kg per hour, but in >40 % with an infusion rate above 8 mg/kg per hour) and there is a trend to similar dose dependency for cardiac failure (*p* = 0.060) and metabolic acidosis (*p* = 0.059), which both occur more frequently with the dose above 4 mg/kg per hour (Additional file 7). The other signs of PRIS occur in similar frequencies across the range of propofol infusion rates. When looking at the influence of the duration of propofol infusion leading to PRIS (Additional file 8), arrhythmia and other ECG changes occur more frequently after 48 hours of infusion. The incidence of rhabdomyolysis and hypertriglyceridaemia rise after 96 hours. On the contrary, metabolic acidosis occurs more frequently in cases reporting a shorter duration of propofol administration leading to the development of PRIS.

## Discussion

Our analysis of 153 published cases of PRIS shows that this syndrome, or at least its reflection in the medical literature, has changed over the last 24 years. A typical patient who died with PRIS in the early 1990s was a child with respiratory infection who developed PRIS after having received an excessive dose of propofol [5–7]. Nowadays, PRIS is more likely to be seen in an adult or elderly patient sedated by a usual dose of propofol in an ICU in whom mild unexplained acidosis and elevation of creatine kinase is noted, sometimes with worsening of AKI and arrhythmia, but other features of PRIS are often missing [23–27]. This may reflect a change of prescription habits, with more ICU patients exposed to propofol, but a smaller proportion of them exposed to dangerously high doses. Our multivariate analysis shows that, after adjustment for covariates, there are only a few independent predictors of death. The most important of these seems to be the cumulative dose of propofol, being represented by both mean infusion rate and the duration of infusion. Out of all the other features of PRIS, which seem to increase the probability of death in univariate analysis, only the presence of TBI and fever were significantly related to PRIS mortality after adjustment for other covariates. Other variables, most notably patient age, the presence of arrhythmia or other ECG changes, were eliminated from the model.

By studying the dependency of the frequency of PRIS signs on the rate and duration of propofol infusion we observed the following associations:

1. Dose-related signs of PRIS occur more frequently with higher infusion rates, irrespective of the duration of infusion. This includes cardiac failure, metabolic acidosis, fever, and perhaps hypotension. Of note, the first two tended to be more frequent in cases caused by a shorter duration of propofol infusion.
2. Signs of PRIS dependent on duration of infusion occur more frequently with longer propofol infusions irrespective of dose; arrhythmia and other ECG changes belong to this category, occurring more frequently in cases where a whole range of propofol doses were administered for more than 48 hours.
3. Signs of PRIS dependent on cumulative dose rise in frequency with both the dose and time of administration. Rhabdomyolysis and hypertriglyceridaemia represent this category and occur most frequently with high doses of propofol after 96 hours of administration.
4. “Idiosyncratic” signs of PRIS occur independently of the rate and duration of infusion. AKI and hepatomegaly belong to this category, even though the latter shows a trend to association with the cumulative dose.

These patterns may reflect the different pathophysiological mechanisms proposed to cause PRIS.

### Proposed pathophysiological mechanisms for PRIS

Mitochondrial toxicity of many anaesthetics used in the 1980s [28] and the structural similarity of propofol to co-enzyme Q led to early formulation of the hypothesis that PRIS is caused by uncoupling or inhibition of the respiratory chain. A series of animal experiments addressed this. Branca et al. [29, 30] exposed isolated rat liver mitochondria to 0–100 μmol/l propofol (plasma concentration in patients sedated with propofol is in the range 5–30 μmol/l). They found a linear dose-dependent reduction of respiratory chain capacity (measured as maximal uncoupled respiration) and transmembranous potential (ΔΨ), but no impairment of ATP synthesis up to 75 μmol/l propofol. It should be stressed that in these experiments succinate was used as a mitochondrial substrate, feeding electrons to Complex II and bypassing Complex I, which is often damaged during mitochondria isolation. Rigoulet et al. [31] later demonstrated that Complex I in isolated rat liver mitochondria is more sensitive to inhibition by propofol than complex II and also demonstrated the ability of propofol (25–400 μmol/l) to cause a leak of protons through the inner mitochondrial membrane, probably via dysfunctional ATP synthase. Mitochondria isolated from rat hearts in similarly designed experiments seemed to be more resistant to propofol toxicity, as reduction of ΔΨ and inhibition of ATP synthesis were only observed at >300 μmol/l propofol [32]. In an ex-vivo study [33] on isolated perfused guinea pig hearts, supratherapeutic propofol concentrations (50–200 μmol/l) caused a delay in myoglobin desaturation and cytochrome c reduction after exposure to ischaemia, consistent with inhibition of the respiratory chain, but not with uncoupling. Of note, these effects were dependent on the dose of propofol, but not on the duration of infusion (0–120 minutes). Decreased activity of cytochrome c oxidase (complex IV) was independently found in skeletal muscle biopsies of two patients with PRIS [34, 35]. The defect was not present in skin fibroblasts suggesting an acquired rather than undetected inborn defect [35]. More recently, significantly reduced activities but normal concentrations of all respiratory complexes were found in a patient who died of PRIS [36], and complex IV was affected most.

New light has now been shed on the conflicting results by a landmark study of Vanlander et al. [36] who in a series of experiments combining a rat model of PRIS and in-vitro exposure of tissue homogenates to propofol demonstrated that propofol in therapeutic concentrations interacts with co-enzyme Q and blocks electron transfer from complexes I and II to complex III, whilst much higher concentrations of propofol are needed to block the activities of individual complexes. Out of these, the activity of complex IV was the most sensitive to inhibition by propofol and only this complex could be inhibited by the concentrations, which were achieved in tissues of animals treated with propofol.

Another proposed mechanism of PRIS is inhibition of fatty acid oxidation, formulated by Wolf et al. [37, 38] and Withington et al. [39] who observed increased plasma concentrations of acyl derivatives of carnitine in children with PRIS, which normalised after cessation of propofol infusion, suggesting a propofol-induced defect of fatty acid oxidation [40]. Also, in one case, PRIS seemed to be triggered by a ketogenic diet [41]. Critical illness itself causes a switch from carbohydrate utilisation to oxidation of lipids. High levels of stress hormones and insulin resistance activate endogenous lipolysis and plasma free fatty acid levels increase [42]. The lipid vehiculum of propofol may further increase the burden imposed on free fatty acid oxidation. Duration of exposure to propofol seems to be a crucial factor in the development of the defect of fatty acid oxidation: in a patient on a propofol infusion (4.1–6.6 mg/kg per hour) [38] plasma C4-carnitine species rose steadily over the period of 5 days. The mechanism by which propofol influences fatty acid oxidation is unknown. One animal study [43] demonstrated an inhibition of the transport of fatty acid into mitochondria at the level of carnitine-acyl transferase I. This enzyme is activated by adenosine monophosphate-activated kinase (AMPK). If propofol interferes with signalling function of co-enzyme Q [36], it may attenuate the activation of fatty acid oxidation by AMPK [44], but the activity of this enzyme has been increased in the myocardium of propofol-sedated rabbits [45]. Finally, propofol triggered apoptosis in human endothelial cell lines and increased vascular permeability in mice [46], but the doses required to elicit these effect were orders of magnitude higher than those seen in clinical situations.

In summary, current experimental data suggest that propofol, structurally similar to co-enzyme Q, interferes with electron flux from upstream complexes (I and II) to complex III. In addition it interferes with fatty acid oxidation, which builds up in plasma over time. Very high doses of propofol seem to directly inhibit the activity of complex IV and uncouple the respiratory chain by modifying the structure of F_1_F_0_ATPase.

Our finding that mechanical cardiac failure and metabolic (in most cases lactic [12, 34, 47–50]) acidosis occur early (often within the first few hours [7, 23, 34, 51–53]) and in a dose-dependent manner, is consistent with a direct inhibition of aerobic phosphorylation observed in experimental studies. The heart is highly reliant on aerobic ATP synthesis [54] and acidosis may represent a combination of hypoperfusion and cytopathic hypoxia in tissues. Fever is reported more frequently with higher propofol infusion rates and this may reflect mitochondrial uncoupling and energy dissipation as heat. Fever is also an independent predictor of mortality in our model, possibly identifying subgroups of patients who are more susceptible to mitochondrial uncoupling. On the other hand, arrhythmia and ECG changes occurred more frequently in PRIS cases caused by prolonged propofol infusions and were the only signs which were not seen in animal models of PRIS triggered by short-term (<38 hours) high-dose propofol [36, 43, 45, 55]. A possible explanation is that arrhythmia and ECG changes are caused by elevated free fatty acids, which steadily increase over days during propofol administration [37] and are known to be proarrhythmogenic [56] (even more so in combination with metabolic acidosis). Similarly, rhabdomyolysis (a common feature of inborn defects of fatty acid oxidation [57, 58]) is associated with the duration of propofol administration and may also be related to the propofol-induced defect of fatty acid oxidation. The frequency of hypertriglyceridaemia seems to also increase with cumulative dose of propofol. If the dose of lipid emulsions exceeds the capacity of hydrolysis in plasma, triacylglyceroles accumulate in the blood and are taken up by the reticulo-endothelial system, causing hepatosplenomegaly, jaundice, and clotting disturbances (“fat overload syndrome” [59]). Recommended lipid dose for parenteral nutrition is 29–54 mg/kg per hour [60]. To match this dose, propofol as a 1 % solution in 10 % intralipid has to run at 2.9–5.4 mg/kg per hour. Most of the patients with PRIS exceeded this rate. Nonetheless, it seems that excessive doses of lipid emulsions are generally well tolerated; rapid infusions of lipid emulsions even in a range of 170–5000 mg/kg per hour have been accidentally administered without side effects [61]. In animal models of PRIS [45, 55], elevated triglycerides was the only sign observed in control animals receiving intralipid alone. In our study, even in the subgroup of patients with infusions at a rapid rate administered over an extended period of time, only a minority (40 %) developed hypertriglyceridaemia and this symptom had no relation to the outcome of PRIS. Possible explanation is that hypertriglyceridaemia represents an epiphenomenon rather than a genuine part of PRIS. Other features of lipid overload syndrome were absent in our series of patients with PRIS, with the exception of one patient with hyperbilirubinaemia [9]. AKI is reported in 39 % of patients with PRIS. It was associated with rhabdomyolysis in our series (R = 0.28, *p* = 0.0004) and myoglobin casts were found in kidneys of rabbits treated with propofol [55], but still there is no link to propofol dose or duration of infusion. It is likely that propofol may have been only a minor factor among many others causing AKI in sick ICU patients.

From the clinical perspective it is important to note that exceeding recommended doses of propofol is not only the main risk factor for the development of PRIS [16, 20], but also that the probability that PRIS will be deadly also increases—at least among the published cases of PRIS. On the other hand, our analysis shows that PRIS can develop with doses well within the recommended safety limits and even after a relatively short duration of administration. In these situations some typical features are often missing, which can make the diagnosis challenging. For example, with doses <4 mg/kg per hour the frequencies of hypertriglyceridaemia, fever, hepatomegaly and heart failure are <5 % in published cases of PRIS, while after short infusions of higher doses, ECG changes and arrhythmia may not be present and PRIS can be manifested as unexplained cardiac failure and metabolic acidosis. Propofol should automatically be suspected if any of the signs of PRIS appear without alternative explanation in an ICU patient on propofol infusion. There is no specific treatment for PRIS and the only way to prevent further deterioration and death is to recognize PRIS early and stop the infusion. Supplementation of co-enzyme Q may become a promising therapeutic strategy (a propofol “antidote”) in the near future [36].

The main limitation of our study is the risk of publication bias, i.e. that the published cases are not representative of the population of patients with PRIS. For example, in our mortality analysis, alternative explanations to a seemingly obvious causative relation between PRIS mortality and propofol cumulative dose is that non-fatal cases of PRIS may be more likely to be written up and published if caused by only a small dose of propofol. Analogously, after safety limits of dosage were introduced, authors may become less likely to report cases of PRIS caused by propofol doses which had exceeded these limits. Apart from the fact that not all cases of PRIS are recognised and published, there is no guarantee that all published cases analysed by us were reporting a condition causally related to propofol administration. PRIS is difficult to identify and to characterise in the critically ill patient because of the overlap of the syndrome with common ICU clinical conditions and our analysis is reliant on interpretation of clinical signs by the authors of original case reports. It is possible that the occurrence of a sign after the start of propofol infusion was just coincidental manifestation of another condition rather than a genuine manifestation of PRIS. Similarly, death reported to be due to PRIS might have occurred anyway, without a causal relation to propofol infusion. In light of this, our mortality analysis must be interpreted with caution. Unfortunately, obtaining prospectively collected data in this relatively rarely occurring syndrome would be extremely difficult. Adverse drug reaction databases may be an alternative source of information, potentially affected less by publication bias, but the main problem will be the large proportion of patients with missing data. The European Medicines Agency only provides cumulative data with basic demographic data and outcome [21], but further details of individual cases are not accessible. Fong et al. [62] looked up PRIS symptoms in the FDA MedWatch [63] database and aimed to determine predictors of death. Unfortunately, the outcome was reported for less than one-third of patients and among them the propofol infusion rate and duration were reported in only 18 % of cases, forcing the authors to remove these key variables from their mortality analysis. After doing so they found cardiac symptoms, rhabdomyolysis, hypotension, metabolic acidosis, renal failure, and age to affect survival in populations with at least one sign of PRIS. Even though most of these signs were associated with mortality in univariate analysis in our series, they were not significant in the multivariate model.

## Conclusions

In conclusion, after propofol labelling information updates in 2001 and 2006, PRIS case reports continue to occur with unchanged frequency, but its presentation has evolved. More survivable cases, occurring with lower infusion rates, have replaced lethal PRIS with multiple clinical manifestations. In multivariate logistical analysis, the rate and duration of propofol infusion, the presence of TBI and fever were associated with higher mortality in published cases of PRIS. Most experimental data point towards mitochondrial toxicity of propofol. We hypothesise that short-term administration of high doses of propofol may cause metabolic acidosis and heart failure by inhibiting aerobic phosphorylation and fever by mitochondrial uncoupling. With prolonged infusions, fatty acid oxidation is likely inhibited by propofol or its metabolite, which causes arrhythmia and other ECG changes. Rhabdomyolysis occurs if the administered dose is high enough, and in some cases this may exacerbate AKI. Hypertriglyceridaemia in the context of PRIS may be an epiphenomenon caused by fat overload.

## Key messages

- Propofol infusion syndrome (PRIS) may occur even after short duration of infusion and with moderate doses (<4 mg/kg per hour). In this situation, diagnosis may be challenging as most typical symptoms are missing.
- Overall mortality in reported cases of PRIS is 51 % and is decreasing over time.
- Propofol infusion rate and duration, the presence of TBI and fever are factors independently associated with mortality in reported cases of PRIS.
- Metabolic acidosis and heart failure occur early and in a dose-dependent manner, whilst rhabdomyolysis, arrhythmia and other ECG changes are rather dependent on the duration of propofol infusion.
- Experimental data suggest that early, dose-dependent signs may be caused by inhibition of respiratory chain, whilst late, time-dependent signs by inhibition of fatty acid oxidation.

## Additional files

Additional file 1: Part S1. Table of published case reports between 1990 and 2014 including complete list of references. **Part S2.** Statistical analysis: step-by-step multiple logistic regression. (DOCX 1948 kb) Additional file 2: Figure S1. Cumulative number of published cases of PRIS between 1990 and 2014 (left) and proportion of fatal cases published each year (right). Dashed line shows a trend determined by linear regression. (PNG 577 kb) Additional file 3: Figure S2. The evolution of patients’ age, cumulative propofol dose, propofol infusion duration and mean propofol infusion rate leading to PRIS in published cases between 1990 and 2014. Each dot represents an individual case, the lines are trends determined by linear regression from all patients. (PNG 455 kb) Additional file 4: Figure S3. Frequency of symptoms in reported cases of PRIS. Data presented as means, vertical bars are 95 % confidence intervals. (PNG 356 kb) Additional file 5: Figure S4A. Adjusted odds ratio of factors influencing mortality of PRIS in multivariate model. (PNG 224 kb) Additional file 6: Figure S4B. Receiver operator curve (ROC) of the final model. (PNG 304 kb) Additional file 7: Figure S5. Relative frequency (y axis) of symptoms of PRIS in patients divided into four quartiles of reported propofol infusion rate. Data presented as means, vertical bars are 95 % confidence interval. (PNG 375 kb) Additional file 8: Figure S6. Relative frequency (y axis) of symptoms of PRIS in patients divided into four quartiles of reported propofol infusion duration. Data presented as means, vertical bars are 95 % confidence intervals. (PNG 389 kb)

## Abbreviations

ΔΨ: Transmembranous potential
AKI: Acute kidney injury
AMPK: Adenosine monophosphate-activated kinase
ECG: Electrocardiograph
FDA: Food and Drug Administration
ICU: Intensive care unit
PRIS: Propofol infusion syndrome
TBI: Traumatic brain injury

## References

[1] Tiwari R. U.S. Anesthesia Drugs Market Worth $3 Billion + by 2018. http://www.prnewswire.com/news-releases/us-anesthesia-drugs-market-worth--3-billion-by-2018-285487841.html. Accessed 6 May 2015.

[2] Global market study on propofol. http://www.aranca.com/downloads/case-studies/business-research/vertical-expertise/global-market-study-on-propofol-2-pharma-and-medical-devices-br-case-study.pdf. Accessed 6 May 2015.

[3] Lonardo NW, Mone MC, Nirula R, Kimball EJ, Ludwig K, Zhou X (2014). Propofol is associated with favorable outcomes compared with benzodiazepines in ventilated intensive care unit patients. Am J Respir Crit Care Med.

[4] Fraser GL, Devlin JW, Worby CP, Alhazzani W, Barr J, Dasta JF (2013). Benzodiazepine versus nonbenzodiazepine-based sedation for mechanically ventilated, critically ill adults: a systematic review and meta-analysis of randomized trials. Crit Care Med.

[5] Notis fra Bivirkningsnaenet. Propofol (Diprivan) bivirkninger. [Adverse effects of propofol (Diprivan)]. Ugeskr Laeger. 1990;152(16):1176. 2330646

[6] Parke TJ, Stevens JE, Rice AS, Greenaway CL, Bray RJ, Smith PJ (1992). Metabolic acidosis and fatal myocardial failure after propofol infusion in children: five case reports. BMJ.

[7] Bodd E, Endresen L (1992). Use of propofol for children. Tidsskr Nor Laegeforen.

[8] Kirkpatrick M, Cole G (1992). Propofol infusion in children. BMJ.

[9] Barclay K, Williams AJ, Major E (1992). Propofol infusion in children. BMJ.

[10] Bray RJ (1995). Fatal myocardial failure associated with a propofol infusion in a child. Anaesthesia.

[11] Strickland RA, Murray MJ (1995). Fatal metabolic acidosis in a pediatric patient receiving an infusion of propofol in the intensive care unit: is there a relationship?. Crit Care Med.

[12] Marinella MA (1996). Lactic acidosis associated with propofol. Chest.

[13] Hanna JP, Ramundo ML (1998). Rhabdomyolysis and hypoxia associated with prolonged propofol infusion in children. Neurology.

[14] Perrier ND, Baerga-Varela Y, Murray MJ (2000). Death related to propofol use in an adult patient. Crit Care Med.

[15] Stelow EB, Johari VP, Smith SA, Crosson JT, Apple FS (2000). Propofol-associated rhabdomyolysis with cardiac involvement in adults: chemical and anatomic findings. Clin Chem.

[16] Cremer OL, Moons KG, Bouman EA, Kruijswijk JE, de Smet AM, Kalkman CJ (2001). Long-term propofol infusion and cardiac failure in adult head-injured patients. Lancet.

[17] U.S. Food and Drug Administration MedWatch. 2001 safety alerts for drugs, biologics, medical devices, and dietary supplements. Diprivan (propofol). http://www.fda.gov/medwaTCH/SAFETY/2001/safety01.htm#diprivan%5D. Accessed 23 Dec 2014.

[18] U.S. Food and Drug Administration. MedWatch. Detailed view: safety labeling changes approved by FDA Center for Drug Evaluation and Research (CDER)—February 2007. Diprivan (propofol) injectable emulsion for IV administration. http://www.fda.gov/Medwatch/SAFETY/2007/feb07.htm#Diprivan. Accessed 23 Dec 2014.

[19] Ahlen K, Buckley CJ, Goodale DB, Pulsford AH (2006). The ‘propofol infusion syndrome’: the facts, their interpretation and implications for patient care. Eur J Anaesthesiol.

[20] Summary of Product Characteristics: PROPOFOL. https://www.medicines.org.uk/emc/medicine/2275#UNDESIRABLE_EFFECTS. Accessed 6 May 2015.

[21] European Medicines Agency. European database of suspected drug reaction reports. https://bi.ema.europa.eu/analyticsSOAP/saw.dll?PortalPages. Accessed 6 May 2015.

[22] Bray RJ (1998). Propofol infusion syndrome in children. Pediatr Anesth.

[23] Chukwuemeka A, Ko R, Ralph-Edwards A (2006). Short-term low-dose propofol anaesthesia associated with severe metabolic acidosis. Anaesth Intensive Care.

[24] Roberts RJ, Barletta JF, Fong JJ, Schumaker G, Kuper PJ, Papadopoulos S (2009). Incidence of propofol-related infusion syndrome in critically ill adults: a prospective, multicenter study. Crit Care.

[25] Richter S, Brugada P (2012). Propofol-induced coved-type electrocardiogram during catheter ablation of paroxysmal atrial fibrillation. A case of Brugada syndrome?. Herzschrittmacherther Elektrophysiol.

[26] Schroeppel TJ, Fabian TC, Clement LP, Fischer PE, Magnotti LJ, Sharpe JP (2014). Propofol infusion syndrome: a lethal condition in critically injured patients eliminated by a simple screening protocol. Injury.

[27] Linko R, Laukkanen A, Koljonen V, Rapola J, Varpula T (2014). Severe heart failure and rhabdomyolysis associated with propofol infusion in a burn patient. J Burn Care Res.

[28] Rottenberg H (1983). Uncoupling of oxidative phosphorylation in rat liver mitochondria by general anesthetics. Proc Natl Acad Sci U S A.

[29] Branca D, Roberti MS, Lorenzin P, Vincenti E, Scutari G (1991). Influence of the anesthetic 2,6-diisopropylphenol on the oxidative phosphorylation of isolated rat liver mitochondria. Biochem Pharmacol.

[30] Branca D, Roberti MS, Vincenti E, Scutari G (1991). Uncoupling effect of the general anesthetic 2,6-diisopropylphenol in isolated rat liver mitochondria. Arch Biochem Biophys.

[31] Rigoulet M, Devin A, Avéret N, Vandais B, Guérin B (1996). Mechanisms of inhibition and uncoupling of respiration in isolated rat liver mitochondria by the general anesthetic 2,6-diisopropylphenol. Eur J Biochem.

[32] Branca D, Vincenti E, Scutari G (1995). Influence of the anesthetic 2,6-diisopropylphenol (propofol) on isolated rat heart mitochondria. Comp Biochem Physiol C Pharmacol Toxicol Endocrinol.

[33] Schenkman KA, Yan S (2000). Propofol impairment of mitochondrial respiration in isolated perfused guinea pig hearts determined by reflectance spectroscopy. Crit Care Med.

[34] Mehta N, DeMunter C, Habibi P, Nadel S, Britto J (1999). Short-term propofol infusions in children. Lancet.

[35] Cray SH, Robinson BH, Cox PN (1998). Lactic acidemia and bradyarrhythmia in a child sedated with propofol. Crit Care Med.

[36] Vanlander AV, Okun JG, de Jaeger A, Smet J, De Latter E, De Paepe B (2015). Possible pathogenic mechanism of propofol infusion syndrome involves coenzyme Q. Anesthesiology.

[37] Wolf A, Weir P, Segar P, Stone J, Shield J (2001). Impaired fatty acid oxidation in propofol infusion syndrome. Lancet.

[38] Wolf AR, Potter F (2004). Propofol infusion in children: when does an anesthetic tool become an intensive care liability?. Paediatr Anaesth.

[39] Withington DE, Decell MK, Al AT (2004). A case of propofol toxicity: further evidence for a causal mechanism. Paediatr Anaesth.

[40] Rinaldo P, Cowan TM, Matern D. Acylcarnitine profile analysis. http://www.nature.com/gim/journal/v10/n2/pdf/gim200822a.pdf. Accessed 6 May 2015. 10.1097/GIM.0b013e3181614289 18281923

[41] Baumeister FA, Oberhoffer R, Liebhaber GM, Kunkel J, Eberhardt J, Holthausen H (2004). Fatal propofol infusion syndrome in association with ketogenic diet. Neuropediatrics.

[42] Cree MG, Wolfe RR (2008). Postburn trauma insulin resistance and fat metabolism. Am J Physiol Endocrinol Metab.

[43] Tong XX, Kang Y, Liu FZ, Zhang WS, Liu J (2010). Effect of prolonged infusion of propofol on the liver mitochondria respiratory function in rabbits. Sichuan Da Xue Xue Bao Yi Xue Ban.

[44] Lee SK, Lee JO, Kim JH, Kim N, You GY, Moon JW (2012). Coenzyme Q10 increases the fatty acid oxidation through AMPK-mediated PPARα induction in 3 T3-L1 preadipocytes. Cell Signal.

[45] Jiang W, Yang ZB, Zhou QH, Huan X, Wang L (2012). Lipid metabolism disturbances and AMPK activation in prolonged propofol-sedated rabbits under mechanical ventilation. Acta Pharmacol Sin.

[46] Lin MC, Chen CL, Yang TT, Choi PC, Hsing CH, Lin CF (2012). Anesthetic propofol overdose causes endothelial cytotoxicity in vitro and endothelial barrier dysfunction in vivo. Toxicol Appl Pharmacol.

[47] Kill C, Leonhardt A, Wulf H (2003). Lacticacidosis after short-term infusion of propofol for anaesthesia in a child with osteogenesis imperfecta. Paediatr Anaesth.

[48] Koch M, De Backer D, Vincent JL (2004). Lactic acidosis: an early marker of propofol infusion syndrome?. Intensive Care Med.

[49] Holzki J, Aring C, Gillor A (2004). Death after re-exposure to propofol in a 3-year-old child: a case report. Paediatr Anaesth.

[50] Romero PC, Morales RM, Donaire RL, Llanos VO, Cornejo RR, Gálvez AR (2008). Severe lactic acidosis caused by propofol infusion: report of one case. Rev Med Chil.

[51] Burow BK, Johnson ME, Packer DL (2004). Metabolic acidosis associated with propofol in the absence of other causative factors. Anesthesiology.

[52] Haase R, Sauer H, Eichler G (2005). Lactic acidosis following short-term propofol infusion may be an early warning of propofol infusion syndrome. J Neurosurg Anesthesiol.

[53] Westhout FD, Muhonen MG, Nwagwu CI (2007). Early propofol infusion syndrome following cerebral angiographic embolization for giant aneurysm repair. Case report. J Neurosurg.

[54] Neubauer S (2007). The failing heart—an engine out of fuel. N Engl J Med.

[55] Ypsilantis P, Politou M, Mikroulis D, Pitiakoudis M, Lambropoulou M, Tsigalou C (2007). Organ toxicity and mortality in propofol-sedated rabbits under prolonged mechanical ventilation. Anesth Analg.

[56] Jouven X, Charles MA, Desnos M, Ducimetière P (2001). Circulating nonesterified fatty acid level as a predictive risk factor for sudden death in the population. Circulation.

[57] Stanley CA, Bennett MJ, Mayatepek E (2006). Disorders of mitochondrial fatty acid oxidation and related metabolic pathways. Inborn metabolic diseases.

[58] Terrone G, Ruoppolo M, Brunetti-Pierri N, Cozzolino C, Scolamiero E, Parenti G (2014). Child neurology: recurrent rhabdomyolysis due to a fatty acid oxidation disorder. Neurology.

[59] Heyman MB, Storch S, Ament ME (1981). The fat overload syndrome. Report of a case and literature review. Am J Dis Child.

[60] Adolph M, Heller AR, Koch T, Koletzko B, Kreymann KG, Krohn K (2009). Lipid emulsions—Guidelines on Parenteral Nutrition, Chapter 6. Ger Med Sci.

[61] Gura KM, Puder M (2010). Rapid infusion of fish oil-based emulsion in infants does not appear to be associated with fat overload syndrome. Nutr Clin Pract.

[62] Fong JJ, Sylvia L, Ruthazer R, Schumaker G, Kcomt M, Devlin JW (2008). Predictors of mortality in patients with suspected propofol infusion syndrome. Crit Care Med.

[63] Commissioner OOT. MedWatch: The FDA Safety Information and Adverse Event Reporting Program. http://www.fda.gov/Safety/MedWatch/. Accessed 6 May 2015.

