# Supplementary material for: Propofol infusion syndrome: a structured review of experimental studies and 153 published case reports
Source: Crit Care. 2015 Nov 12;19:398. doi: 10.1186/s13054-015-1112-5 (PMC4642662; doi:10.1186/s13054-015-1112-5)
Supplement: Additional file 1: Part S1. — Table of published case reports between 1990 and 2014 including complete list of references. Part S2. Statistical analysis: step-by-step multiple logistic regression. (DOCX 1948 kb) [file 13054_2015_1112_MOESM1_ESM.docx]

Additional file 1

To paper: Krajcova et al: **Propofol Infusion Syndrome in 2015: A Systematic Review of Experimental Studies and 153 Published Case Reports**

Content:

Part S1: Table of published case reports between 1990 and 2014 incl. complete list of references

Part S2: Step-by-step multiple logistic regression

# Part S1: Table of published case reports of PRIS between 1990-2014

| **Reference** | **Age (years)** | **Sex** | **Diagnosis** | **Purpose of propofol infusion** | **Dosage**  **[average rate]** | **Dosage [max. rate] (hours)** | **Duration [days]** | **Clinical features** | **Biochemical analysis** | **ECG changes** | **Concomitant use of vasopressors or corticoids** | **Treatment** | **Histology/other examination** | **Outcome** |
| --- | --- | --- | --- | --- | --- | --- | --- | --- | --- | --- | --- | --- | --- | --- |
| Ugeskr Laeger^1^ *1990* | 2 | F | Croup | S | 10 | NA | 4 days | Hypotension, hepatomegaly, heart failure, multiorgan failure of unknown etiology | Metabolic acidosis | NA | NA | NA | NA | Died |
| Parke et al.^2^  *1992* | <3 | F | Laryngotracheobronchitis | S | 7.5 | 11.5 (6) | 5 (115 hours) | Fever, arrhythmia, hepatomegaly | Metabolic acidosis, lipaemia | Nodal bradycardia  with a right bundle branch block pattern → pharmacologically resistant asystole | NA | Ionotropes (dobutamine, dopamine, isoprenaline, adrenaline), atropine, Tris buffer | Myocardial myocytolysis, microvesicular fatty liver, pulmonary oedema; lumbar punction and abdominal ultrasonography: normal; all cultures negative | Died |
|  | <2 | F | Laryngotracheobronchitis | S | 7.4 | 8.0 (29) | 3 (66 hours) | Fever, arrhythmia, hepatomegaly, oliguria, pulmonary oedema | Metabolic acidosis, hyperkaelamia, lipaemia | Nodal bradycardia with a right bundle branch block → asystole | NA | Ionotropes: dopamine, dobutamine, adrenaline, isoprenaline, furosemide, peritoneal dialysis, arterio-venous haemofiltration, calcium resonium, glucose-insulin infusion | X-ray scan: bilateral patchy consolidation | Died |
|  | <2 | F | Laryngotracheobronchitis | S | 10.0 | 13.6 (8) | 76 hours | Fever, arrhytmia | Metabolic acidosis, lipaemia; normal liver function and haematologic tests | Bradycardia → procedeed to asystole | Dexamethasone  at dose 0,5 mg/kg | Sodium bicarbonate, ionotropes, pacing electrodes | X-ray scan: bilateral patchy consolidation; all cultures negative; echocardiography: normal; brain CT: normal; Histology: fatty infiltration of liver, bilaterally consolidated lungs with histological changes (probably viral interstitial pneumonia), normal heart, with no evidence of myocarditis | Died |
|  | <1 | F | Bronchiolitis | S | 8.0 | 10.7  (5) | 74 hours | Fever, arrhythmia, hepatomegaly, acute renal failure | Metabolic acidosis, lipaemia, hypocalcemia –serum ionised calcium concentration 0.68 mmol/l | Bradycardia with frequent atrial ectopics | NA | Atropine, isoprenaline,  peritoneal dialysis | Microbiology: respiratory syncytial virus in nasopharyngeal secretion; Echocardiogram: no abnormality but viral myocarditis was postulated | Died |
|  | 6 | M | Laryngotracheobronchitis | S | 8.1 | 9.2 (1) | 104 hours | Hypotension, hepatomegaly, anuria, arrhythmia | Metabolic acidosis, lipaemic serum | Varying PR interval, bradycardia → leading to asystole | NA | Atropine, dobutamine, adrenaline, transvenous pacing | Microbiology: parainfluenza type 2 virus in tracheal aspirate; sputum  sample: Pseudomonas aeruginosa. Histology: no structural abnormality of the heart, no evidence of myocarditis, fatty microvesicular steatosis of liver, cerebral oedema, degenerative changes in the epithelial cells of kidney; | Died |
| Bodd et al.^3^ *1992* | NA | NA | Laryngitis | S | 7 | NA | 4 hours | NA | Metabolic acidosis | NA | NA | NA | NA | Died |
|  | NA | NA | Epiglotitis | S | NA | NA | 96 hours | NA | Metabolic acidosis | NA | NA | NA | NA | Died |
| Kircpatrick et al.^4^  *1992* | <1 (1month) | F | Pertussis (paroxysmal cough, whoop, vomiting) | S | 10 | NA | 4 days | NA (no clinical evidence of haemodynamic compromise) | Lipaemic serum; no metabolic acidosis | NA | NA | Early discontinuation of propofol infusion | NA | Survived |
| Barclay et al.^5^  *1992* | <2 (20 months) | F | Acute epiglottitis | S | 5-10 | 10 | 56 hours | Arrhythmia, hypotension, oliguria, fever, generalised convulsion | Metabolic acidosis (hyperlactatemia), lipaemic serum, ,hyperbilirubinemia, high levels of AST and creatine phosphokinase ˃100,000 U/l; myoglobinuria; leucocytosis | Bradycardia → asystolic arrest | NA | Sodium bicarbonate, adrenaline, dobutamine, adrenaline, calcium infusions, bicarbonate, venovenous haemodiafiltration, peritoneal dialysis | Cranial CT: normal; Microbiology: cerebrospinal fluid – normal; Abdominal ultrasonography: normal; Echocardiogram: normal; Microbiology: negative blood cultures, later candidaemia; Histology – muscle biopsy: focal necrosis with basophilic fibres and histiocytes in skeletal muscle (no evidence of dystrophy or neurogenic atrophy), electronmicroscopy: foci of necrosis with some large mitochondria and distorted cristae | Survived |
| Bray et al.^6^  *1995* | 9 | M | Viral upper respiratory infection | S | 4.5 mg/kg/hour | 6.2 mg/kg/hour | 72 hours | Hepatomegaly, fever, myocardial failure | No metabolic acidosis or lipaemic plasma | T-wave inversion → widened ventricular complexes → bradycardia with complete heart block → disapearance of P-waves, multifocal ventricular complexes → asystole | NA | Atropine, isoprenaline, dobutamine, adrenaline | Microbiology: influenza A virus in nasopharyngeal secretions. X-ray scan: enlarged heart; echocardiogram: impairment of ventricular function, but no structural abnormality; Histology: mild microvesicular steatosis in the liver, some lymphocyte infiltration of the heart, but no histological evidence of myocarditis or damage to the conducting system | Died |
| Strickland et al.^7^  *1995* | 11 | F | Astrocytoma (surgery: frontotemporal craniotomy with subtotal removal of a grade IV astrocytoma) | S | 9.4 | 12 | 38 hours | Hypotension, fever, oliguria, arrhythmia | Metabolic acidosis, hyperkalemia, lipemic serum, leucocytosis, myoglobinuria | Tachycardia → normal sinus rhythm → junctional rhythm with tall peaked T waves → ventricular tachycardia and fibrillation | Dexamethasone (16 mg the first hospital day), methylprednisolone 250 mg the second day, 50 mg the third day) | Dopamine, sodium bicarbonate, atropine, glucose and insuline, fluid replacement, lidocaine, calcium chloride, bretylium, epinephrine, direct cardioversion | CT: astrocytoma, postoperative extensive cerebral edema; Microbiology: Haemophilus influenzae in tracheal secretion | Died |
| Marinella et al.^8^  *1996* | 30 | F | Exacerbation of asthma complicated by acute respiratory failure | S | NA | NA | ˂13 hours | NA | Metabolic lactic acidosis; serum ketones negative | NA | Methylprednisolone | Early discontinuation of propofol infusion | Microbiology: cultures of sputum, urine and blood negative | Survived |
| Plotz et al.^9^  *1996* | 6 | M | Laryngitis | S | 5-10 | NA | 60 hours | Heart failure, arrhythmia, fever | Metabolic acidosis, lipaemic serum, CK ˃ 33 000 U/l, myoglobinuria | Nodal bradycardia andl tachycardia, ventricular tachycardia | NA | Inotropes, dantrolene | NA | Died |
| van Straaten et al.^10^  *1996* | 4 | M | Laryngitis with sub-glottic stenosis | S | 8.6 | NA | 3 days | Rhabdomyolysis, pulmonary hypertension | Lipaemic serum, elevated serum carnitine, CK 127000U/l | NA | NA | Propofol infusion ceased, bicarbonate, veno-venous haemofiltration | NA | Survived |
| Bray et al.^11^ *1998* | 2 | NA | Epiglottitis | S | 5.2 | 9 | 4 days | Enlarged fatty liver, arrhythmia | Metabolic acidosis | Bradycardia → to asystole | NA | NA | NA | Died |
|  | 1 | NA | Laryngitis, encephalitis | S | 6.3 | 8.7 | 4 days | Enlarged fatty liver, arrhythmia | Metabolic acidosis | Bradycardia, AV block | NA | NA | NA | Died |
|  | 3 | NA | NA | S | 200mg/hour | NA | 2 days | An arrhythmia, resp failure | Acidosis/alkalosis | NA | NA | NA | NA | Died |
|  | 1 | NA | Croup | S | NA | NA | 2 days | Cardiac failure | NA | NA | NA | NA | NA | Died |
|  | 0.5 | NA | Con. heart disease | S | 1-6 | 6 | 3 days | Enlarged fatty liver, arrhythmia | NA | Bradycardia, resp failure | NA | NA | NA | Died |
|  | 8 | NA | Status epilepticus | S | 15.2 | 25 | 29 hours | Fever, muscle rigidity, arrhythmia | Metabolic acidosis, creatine phosphokinase 38770, myoglobinuria | Bradycardia → to asystole | NA | NA | NA | Died |
| Cray et al.^12^  *1998* | 10 months | M | Viral upper respiratory infection (upper respiratory obstruction secondary to an esophageal foreign body) | S | 10.0 mg/kg/hour | 12.8 mg/kg/hour | 50.5 hours | Hypotension, arrhythmia, enlarged liver, fever, oliguria, olive green urine | Lipaemic plasma – high triglyceride concentration with a normal cholesterol level; Mixed respiratory alkalosis and severe lactic metabolic acidosis; significantly increased liver transaminases, high serum amylase, hypocalcemia, hypoglycemia, abnormal coagulation, leukocytosis, CPK ˃ 30 000units/L, myoglobinuria; very large peak of lactic acid and a high level of 3-hydroxybutyrate in urine | Bradycardia with first degree atrioventricular block with right bundle branch block → junctional rhythm with right bundle branch block → sinus rhythm | NA | Sodium bicarbonate, dopamine, atropine, external transthoracic pacing, isoproterenol, fluid replacement, epinephrine; plasmapheresis followed by continuous veno-venous hemofiltration (CVVH) | Microbiology: negative bacterial cultures, Parainfluenza III virus in nasopharyngeal aspirate; Blood toxicology screening: propofol metabolite; Abdominal ultrasonography: decreased liver echogenicity; Echocardiogram: a structurally normal heart; Histology – liver biopsy: 10% zone III necrosis with fatty change – similar to those seen in acetaminophen poisoning; muscle biopsy: muscle necrosis associated with histiocytic response, regenerating fibers; analysis of muscle homogenate: a reduction in cytochrome C oxidase aktivity; Skin fibroblasts culture – no underlying respiratory defect; CT scan of the brain: normal | Survived |
| Hanna et al.^13^  *1998* | 17 | M | Refractory status epilepticus | S | NA | 228µg/kg/min | 44 hours | Hypotension, hypoxemia, arrhythmia, fever, rusty brown urine, anuria | Metabolic acidosis (lactate 19 mmol/L), hyperkalemia, hypocalcaemia, creatine kinase 83,000U/L, WBC 7,900/µL, | Wide complex bradycardia with 3- to 5- second episodes of asystole | NA | Neosynephrine, dopamine, coloids, sodium bicarbonate, calcium gluconate, atropine, dialysis | Microbiology: blood, urine, sputum specimens cultured for bacteria sterile; Histology: nyocytolysis of skeletal muscles, rhabdomyolysis of the diaphragm, sediment consistent with myoglobin filled the renal tubules; mild acute bronchopneumonia; transesophageal echocardiography and lower extremity venous ultrasonography – normal, pulmonary embolism unlikely | Died |
|  | 7 | M | Refractory status epilepticus | S | NA | 449µg/kg/min | 63 hours | Tea-colored urine, anuria, hypotension, arrhythmia, fever, hypoxia | Metabolic acidosis (lactate 24.9 mmol/L), CK 49,992 U/L, leukocytosis WBC 24,950/µL | Tachycardia → wide complex tachycardia → bradycardia with 3- to 5-second episodes of asystole | Hydrocortisone | Dobutamine, dopamine, epinephrine, phenylephrine, dialysis | Microbiology: blood, urine, sputum specimens cultured for bacteria sterile; Chest radiograph: no infiltrate; Echocardiography: normal left ventricular function, no evidence of elevated right ventricular pressure – pulmonary embolism unlikely; Histology: myocytolysis of skeletal muscles, renal tubules contained amorphous debris consistent with myoglobin casts; patchy aspiration bronchopneumonia | Died |
| Mehta et al.^14^  *1999* | 18 months | F | Elective surgery for bilateral talipes correction (arthrogryposis multiplex congenita) | A | 6mg/kg | NA | 5 hours | Oligoanuria, renal failure, arrhythmia, myocardial failure, low oxygen saturation | Metabolic acidosis (lactate 3.4mmol/l), hypertriglyceridaemia 3.4 mmol/l, normal serum concentrations of aminoacids and organic acids; no myoglobinuria | Persistant bradyarrhythmias | NA | Bicarbonate, volume resuscitation, ionotropic support, peritoneal dialysis | Chest radiograph: consistent with acute lung injury and pulmonary aspiration of gastric contents; Histology – muscle biopsy: decreased complex IV activity, lowered cytochrome oxidase ratio (0.004), possible mitochondrial respiratory-chain enzyme deficiency; mirobiological and virological tests were negative | Survived |
| Perrier et al.^15^  *2000* | 18 | M | Trauma head injury + multiple bone fractures | S | Bolus of 220 mg + propofol infusion at a rate of 50-55 mg/hour | 530-700mg/hour (chyba???) | 98 hours | Hypotension, fever, arrhythmia | Metabolic acidosis, lipemic serum, hyperkalaemia 6.4mEq/dL, increased methemoglobin 13%, myoglobinuria, increased creatine kinase concentration 20,520 U/L (also due to traumatic insult of extremities) | Sinus rhythm → atrial fibrillation with rapid ventricular response → a nonspecific intraventricular conduction block, with a possible anterolateral infarction and a new left axis deviation → a left bundle-branch block with bradycardia → development of pulseless electrical activity followed by asystole | NA | Atropine, epinephrine, fluid boluses, diuretics, bicarbonate | Microbiology: Gram-negative bacilli in tracheal secretion; blood and urine cultures negative; Chest radiograph: pneumomediastinum, and no change in previously noted bilateral pulmonary infiltrates; Echocardiography: global hypokinesis and no evidence of pericardial effusion; | Died |
| Stelow et al.^16^  *2000* | 47 | F | Exacerbation of asthma | S | NA | 200µg/kg/min | ˂5days | Anuria, severe hypotension, fever, arrhythmia, darkened urine | Creatine kinase activity 762,000U/L, troponin I (cTnI) concentration 4µg/L, hyperkalemia, metabolic acidosis, hypocalcemia, white blood cell count increased, possible hematuria | Arrhythmias and episodes of ventricular tachycardia → cardiac arrest → supraventricular rhythm | Corticosteroids | Fluid replacement, diuretics, bicarbonate therapy, calcium replacement, dopamine, phenylephrine, haemodialysis, dantrolene | Chest X-ray scan: possible infiltrates; Skeletal muscle histology:  a disorganization of myofibrils and  sarcomeres. Most of the muscle fibers showed an  acute necrotic reaction with swelling, loss of striations,  and vacuoles, degeneration of nuclei.  Sections of the heart revealed numerous focal areas of myofibril degeneration surrounded by an acute inflammatory  reaction with macrophages and neutrophils.  Presence of myoglobin casts in ˃50%  of the tubular lumen in kidney - acute tubular necrosis | Died |
|  | 41 | M | Exacerbation of asthma | S | NA | 222µg/kg/min | ˂6 days | Oliguria, brown urine | Serum myoglobin concentration 6800µg/L, creatine kinase activity 204,000U/L, cTnI 46µg/L, hematuria | NA | Corticosteroids | Diuretic and fluid therapy | Echocardiography: globally reduced left ventricular function without a focal lesion | Survived |
| Badr et al.^17^  *2001* | 21 | F | Left hemispheric arteriovenous malformation (treatment: embolization of the arteriovenous malformation) | S | 75-150ug/kg/min | 150ug/kg/min | ˃48 hours | Hemodynamic deterioration | Metabolic acidosis (hyperlactataemia), normal creatinine measurements, serum chloride concentration 107-115 mm | NA | Dexamethasone | Sodium bicarbonate, dopamine | Cerebral angiography: a diffuse, left parietal arteriovenous malformation with hemorrhage from the anterior communicating and middle cerebral arteries | Died |
| Cannon et al.^18^  *2001* | 13 | F | Trauma head injury | S | 100ug/kg/min | NA | 4 days | Green urine, fever, hypotension, arrhythmia, myocardial failure, acute renal failure, rhabdomyolysis | Leukocytosis, metabolic acidosis (nonlactate), increased ALT, AST, creatinine 2.1mg/dl, CK MB isoenzyme 1053U/L, CK 389.000U/L, troponin I 0.1ng/ml, total lactate dehydrogenase 15524U/L; lactate dehydrogenase isoenzyme 1 261U/L | Right bundle branch block → bizzare wide QRS complexes | NA | Dopamine, epinephrine, intravascular volume support | CT of the brain: right frontal and temporal contusion, SAH, a small right subdural hematoma;  Microbiology: Staphylococcus aureus in tracheal aspirate; Histology – skeletal muscle: focal necrosis of myocytes with surrounding inflammatory cells, which was consistent with a diagnosis of focal rhabdomyolysis; kidney: myoglobin cast nephropathy; pneumonia, no hepatic steatosis | Died |
| Cremer et al.^19^  *2001* | ≥ 16-55≤ | NA | Head injury | S | 7.3mg/kg/hour (propofol formulation 2%) | NA | ˃58 hours | NA | No metabolic acidosis, no lipaemic serum,  hyperkalaemia, creatine kinase concentration >4000 U/L or myoglobin concentration >1000 µg/L | Ventricular tachycardia | NA | NA | Histology: muscle-cell degradation; Echocardiography: normal | Died |
|  | ≥ 16-55≤ | NA | Head injury | S | 5.7mg/kg/hour (propofol formulation 2%) | NA | ˃58 hours | NA | Metabolic acidosis, no lipaemic serum, hyperkalaemia; Creatine kinase concentration >4000 U/L or myoglobin concentration >1000 µg/L | Sinus tachycardia, supraventricular tachycardia | NA | NA | Histology: muscle-cell degradation, echocardiography: poor contractility | Died |
|  | ≥ 16-55≤ | NA | Head injury | S | 6.6mg/kg/hour (propofol formulation 2%) | NA | ˃58 hours | NA | Metabolic acidosis, hyperkalaemia, lipaemic serum;  Triglyceride concentrations >3·0 mmol/L | Atrial fibrillation, ventricular tachycardia | NA | NA | NA | Died |
|  | ≥ 16-55≤ | NA | Head injury | S | 5.5mg/kg/hour (propofol formulation 2%) | NA | ˃58 hours | NA | Metabolic acidosis, no lipaemic serum, creatine kinase concentration >4000 U/L or myoglobin concentration >1000 µg/L | Sinus tachycardia, idioventricular rhythm | NA | NA | Histology: muscle-cell degradation; Echocardiography: large right ventricle | Died |
|  | ≥ 16-55≤ | NA | Head injury | S | 7.4mg/kg/hour (propofol formulation 2%) | NA | ˃58 hours | NA | Metabolic acidosis, hyperkalaemia; Creatine kinase concentration >4000 U/L or myoglobin concentration >1000 µg/L | Supraventricular tachycardia, ventricular tachycardia | NA | NA | Histology: muscle-cell degradation; Echocardiography: poor contractility | Died |
|  | ≥ 16-55≤ | NA | Head injury | S | 5.8mg/kg/hour (propofol formulation 1%) | NA | ˃58 hours | NA | Metabolic acidosis, hyperkalaemia, lipaemic serum; Triglyceride concentrations >3·0 mmol/L | Supraventricular tachycardia, nodal rhythm, ventricular tachycardia | NA | NA | NA | Died |
|  | ≥ 16-55≤ | NA | Head injury | S | 6.9mg/kg/hour (propofol formulation 1%) | NA | ˃58 hours | NA | Metabolic acidosis, hyperkalaemia, lipaemic serum; Triglyceride concentrations >3·0 mmol/L | Sinus tachycardia, idioventricular rhythm | NA | NA | NA | Died |
| Wolf et al.^20^  *2001* | 2 | M | Trauma head injury | S | 5.2mg/kg | 5.4mg/kg | 72 hours | Oliguria, impaired renal function | Hyperkaleamia, increased serum concentration of urea, creatinine; metabolic acidosis (raised plasma lactate), raised malonylcarnitine, C5-acylcarnitine, creatine kinase, troponin T, serum triglyceride, myoglobinaemia | Sudden and persistant nodal bradycardia (28/min) | NA | Isoprenaline infusion, transvenous pacing, haemofiltration | NA | Survived |
| Kelly et al.^21^  *2001* | NA (adult) | NA | Severe closed-head injury | S | 126 ug/kg/min | 200ug/kg/min | 55 hours | Renal failure, cardiovascular collapse | Metabolic acidosis | NA | NA | NA | NA | Died |
| Friedman et al.^22^  *2002* | 23 | F | Status epilepticus | S | 12mg/kg/hour | NA | 106 hours | Arrhythmia, cardiac failure, acute renal failure | Metabolic acidosis, hyperkalaemia | Tachycardia | NA | NA | NA | Died |
| Abrahams et al.^23^  *2002* | 13 | M | Arterio-venous malformation resection | A+S | NA | NA | 4 days | Cardiac failure, rhabdomyolysis, acute renal failure | NA | NA | NA | Propofol infusion ceased, ECMO, haemodialysis | NA | Survived |
| Ernest et al.^24^  *2003* | 31 | M | Trauma head injury | S | 4.1 mg/kg/hour | 6.8mg/kg/hour | 157 hours | Deterioration of renal function, green discolouration of urine, rhabdomyolysis | Creatine kinase 11,044 IU/l, creatinine level of 0.37 mmol/l, metabolic acidosis (serum lactate 0.6mmol/l), lipaemic plasma | Incomplete RBBB with some peaking of the anterior T-waves → lateral T-wave inversion → lateral T wave inversion with inferior flattening and some prolongation of the QT interval → tachycardia, slurred elevation of the ST-T wave segments → developed into inferolateral ST segment elevation with reciprocal anterior changes → bizarre ST-T wave changes and broadening of the QRS complex, giving the appearance of a near „sinusoidal“ rhythm → a polymorphic ventricular tachycardia → ventricular fibrillation | NA | NA | CT scan of the brain: normal → sinusitis; Chest X-ray: no mediastinal or aortic injury; Microbiology: ↑protein level, not positive for infection; A transthoracic echocardiography examination: normal left ventricular size associated with moderate hypertrophy and preserved left ventricular function; An autopsy: no specific pathology to explain the circulatory colapse, rhabdomyolysis or renal impairment, no evidence of an undetected myocardial or aortic injury | Died |
| Kill et al.^25^  *2003* | 7 | M | Osteogenesis imperfecta type 3 – surgical repair of a distal femur fracture + (minor respiratory tract infection) | A | Bolus of 120 mg, 13.5 mg/kg per hour | NA | 150 min | NA, no fever, no enlargement of the liver, no bradyarrhythmias, no signs of myocardial failure | Lactic acidosis | Tachycardia | Methylprednisolone | Propofol infusion discontinuation; frusemide | Chest X-ray: no pathological cardiopulmonary findings | Survived |
| Lewejohann et al.^26^  *2004* | 27 | M | Polytrauma (severe head trauma, multiple fractures of: rips, extremities with severe vascular damage, cervical vertebra, pericardial effusion, hematoma of the spleen, ARDS | S | 25ml/hour | 10-25ml/hod (2% propofol) | 7 days | Severe rhabdomyolysis (initially to a multiple trauma), renal failure | Initial myoglobin level: 6937µg/l (as a result of the multiple trauma), decreased on 3865µg/l on admission. After propofol infusion with maximum rate 25ml/hour: a dramatic increase of the myoglobin level to 17414µg/l… After propofol discontinuation: myoglobin level decreased | NA | Norepinephrine, epinephrine | Hemofiltration (because of renal failure), propofol infusion discontinuation | NA | Survived |
| Koch et al.^27^  *2004* | 5 | F | Endovascular coil embolization of a complex high output arterio-venous malformation of the right middle cerebral artery | S | 15 mg/kg per h | 15 mg/kg per h | 10 hours | NA | Lactic acidosis | Bradycardia | High-dose corticoid treatment; labetalol | *Trigger* conditions: CNS disease, glucocorticoids, catecholamines, beta-blockers | Chest X-ray scan: normal | Survived |
| Holzki et al.^28^  *2004* | 3 | F | Aspiration pneumonia | S | 20mg/kg per hour (initially) | Re-exposure: 4.2 mg/kg per hour | 8 hours (re-exposure of 4.2mg/kg per hour) | Fever, hypotension, hepatomegaly, arrhythmia | Initially: respiratory and metabolic acidosis, (simultaneously bronchospasm. After re-exposure: severe metabolic acidosis; serum glutamate oxalacetate transaminase 2175 U/l, serum glutamate pyruvate  transaminase 1600 U/l, creatine kinase 2000 U/l, lactate 5.1 mmol/l; lipaemic serum | Normal rhythm after first propofol exposure → after re-exposure: bradycardic dysrhythmias, ventricular ectopics, incomplete right bundle-branch block → pronounced conduction disturbances,  broadened QRS patterns | NA | Catecholamines, insertion of cardiac pacemaker – transvenous pacing of the heart | Echocardiography demonstrated normal contractility; X-ray: aspiration pneumonia; lumbar puncture: normal | Died |
| Withington et al.^29^  *2004* | 5 months | M | Operation of cleft lip and palate | A + S | 11.7 mg/kg/hour | 15mg/kg/hour | 61.75 h | Hypotension, oliguria, hepatic dysfunction, acute renal failure with rhabdomyolysis, green brown color of urine, arrhythmia | Lipaemic serum, lactic acidosis, coagulopathy, hyperkalemia, hyperphosphatemia, hypertriglyceridemia, increased levels of acetyl and hydroxy-butyryl species with generalized elevation of fatty acylcarnitine intermediates (especially medium-chain unsaturated and dicarboxylic species) | Wide complex tachycardia with right bundle branch block and left axis deviation → sinus bradycardia with ventricular escape of different morphologies → second and third degree heart block → ventricular tachycardia → supraventricular tachycardia → sinus rhythm | NA | Isoprenaline, dopamine, epinephrine, trancutaneous pacing without improvement…  Charcoal hemoperfusion with success, hemodialysis | NA | Survived |
| Culp et al.^30^  *2004* | 13 | M | Resection of parietal arteriovenous malformation | A + S | Bolus of 100mg; 190µg/kg/min | NA |  | Cardiogenic shock, oliguria, renal failure, rhabdomyolysis, arrhythmia | Severe metabolic acidosis, creatinine 3mg/dL, creatine kinase in the thousands, the cardiac enzymes, including troponin and creatine kinase MB isoenzyme, were within normal limits; myoglobinuria;  Hemolysis, a mild unconjugated hyperbilirubinemia  (2–3 mg/dL) (consistent with continuing  hemolysis) | Sinus rhythm with a normal QT interval → prolonged QT interval and T wave inversions → polymorphic ventricular tachycardia → accelerated junctional  tachycardia → normalization of cardiac rhythm (sinus  rhythm with a normal QT interval) | No steroids | Cardioversion, IV magnesium and potassium supplementation, lidocaine and amiodarone infusions, epinephrine, norepinephrine, bicarbonate;  Extracorporeal circulation with membrane oxygenation | CT scan of the brain: brain edema; Transthoracic echocardiogram: severe biventricular dysfunction, no pericardial effusion, no significant valvular disease → transesophageal echocardiography: normal biventricular function; Chest film: pulmonary edema | Survived |
| Baumeister et al.^31^  *2004* | 10 | M | Status epilepticus (fasting for two days, a classic ketogenic diet: 55kcal/day, containing 90% of energy as long-chain triglycerides (3 days) | S | 5.5-9 mg/kg/hour | 9 | 4 days | Cardio-circulatory instability, fever, rhabdomyolysis, progressive respiratory insufficiency, congestive heart failure, arrhythmia | Hyperlipidemia (serum triglycerides 5200mg/dl; serum cholesterol 440mg/l), rhabdomyolysis (maximum of serum CK 18 900U/l, CK-MB isoenzyme 700U/l, pigmenturia), metabolic acidosis with a lactate of 6.4 mmol/l | Right bundle-branch block, ventricular arrhythmia → polymorphic ventricular tachycardia (torsade de pointes) → after treatment by lidocaine and magnesium it disappeared → bigeminal rhythm and salves of ventricular extrasystoles → after esmolol sinus rhythm → ventricular tachycardia → ventricular fibrillation | NA | Lidocaine, magnesium, dopamine, stopping of propofol infusion and ketogenic diet; glucose-insulin-infusion; esmolol, catecholamines, ajmalin | NA | Died |
| Casserly et al.^32^  *2004* | 42 | M | Cerebral sinus thrombosis, cerebral hemorrhage, hydrocephalus | S | NA | 143 µg/kg/min | 3 days | Acute renal failure, anuria, myocardial dysfunction, arrhythmia | Creatinine level ˃ 451µmol/L, blood urea nitrogen levels 9.9mmol/L, serum bicarbonate 14mmol/L, metabolic acidosis, CK 22.426U/L, serum myoglobin 230mg/dL, troponin 20µg/L | Right bundle branch block with diffuse ST and T-wave changes → asystolic arrest | Phenylephrine (to maintain adequate cerebral perfusion pressure) | Fluid resuscitation - aggressive hydration  (and urine alkalization), norepinephrine | NA | Died |
|  | 17 | F | Polytrauma (including head injury – closed head contusion) – intraabdominal hemorrhage (owing to splenic, hepatic, pancreatic, bladder, and right colonic mesentery lacerations) | S | NA | 118 µg/kg/min | 5 days | Rhabdomyolysis, oliguria | CK 172.833U/L, serum creatinine 512 µmol/L, blood urea nitrogen 16mmol/L,hyperkalemia 5.6mmol/L, bicarbonate 18mol/L, normal troponin levels, urine myoglobin negative | Diffuse ST and T-wave changes | Phenylephrine | Renal replacement therapy, hemodialysis | CT scan of the brain: cerebral edema without herniation; | Survived |
| Burow et al.^33^  *2004* | 31 | F | Radiofrequency ablation of chronic atrial fibrillation | A | 83 µg/kg/min | 125 µg/kg/min | ˃ 6 hours | NA | Metabolic acidosis | NA | NA | Early discontinuation of propofol, sodium bicarbonate | Echocardiography: normal ventricular function with a left ventricular ejection fraction of 55% and mild atrial enlargement; | Survived |
| Salengros et al.^34^  *2004* | 71 | M | Laparoscopic radical prostatectomy | A | NA | 7.8mg/kg/hour | ˃ 4.5 hours | Arrhythmia | Metabolic acidosis | Tachycardia | NA | Early discontinuation of propofol | Transesophageal echocardiography: good left and right contractility, adequate  ventricular filling, and an adequate cardiac output; Microbiology: blood cultures negative; the abdominal cavity was searched for any  septic source or hepatic necrosis, without success. | Survived |
| Haase et al.^35^  *2005* | 7 | M | Craniosynostosis admitted following surgery (11 hours fasting before anesthesia) | A | 100 mg bolus (total dose 6.1 mg/kg BW) | NA | 40 minutes | ↑CK ← surgical trauma or subclinical rhabdomyolysis; Hypotensio, arrhythmia | Metabolic acidosis (lactate 9.4 mmol/l), CK 5.27 µmol/lxs; myoglobin, glutamate oxaloacetate  transaminases, and triglycerides –normal range, urine examination  and urinary output – normal. | Tachycardia | NA | Early discontinuation of propofol infusion | NA | Survived |
| Liolios et al.^36^  *2005* | 42 | M | Brainstem cavernous angioma | A + S | 5.2 (1 hour) –9 mg/kg/hour (3 hours) | 9 mg/kg | 38 hours | Renal function impairment | Lactic acidosis 10.8 mmol/L, CK 3470 IU/L, serum creatinine 1.6 mg/dL, absent ketonuria;  Serum potassium concentration – normal; urinary output was adequate | ECG without abnormalities | Methylprednisolone | Early discontinuation of propofol infusion | NA | Survived |
| Kumar et al.^37^  *2005* | 27 | F | Seizures secondary to hemorrhage from an arteriovenous malformation | S | NA | NA | ˃36 hours | Hypotension, arrhythmia | Metabolic (lactic) acidosis, creatine phophokinase 37.749 U/L, hyperkaelemia, normal troponin and myoglobin level | Wide complex bradycardia → asystole | NA | Fluid resuscitation, epinephrine, dopamine, sodium bicarbonate, calcium chloride, transvenous pacemaker | CT of the brain: a large intraventricular  hemorrhage; echocardiogram: lateral  ventricular wall ischemia; microbiology: all cultures negative | Died |
|  | 64 | M | Status epilepticus | S | NA | NA | ˃24 hours | Hypotension, arrhythmia | Metabolic acidosis (pH=7.16), CPK level 48.000U/L | Bradycardia with a junctional rhythm → asystole | NA | Neosynephrine, dopamine, bicarbonate, calcium | Microbiology: all cultures negative | Died |
|  | 24 | F | Status epilepticus secondary to encephalitis | S | NA | NA | ˃24 hours | Hypotension, arrhythmia | Metabolic acidosis, hyperkaelemia, hypocalcemia | Bradycardia → pulseless electrical activity | NA | Dopamine, transvenous pacing, sodium bicarbonate, insuline with dextrose, calcium chloride | CT of the brain: normal; microbiology: all cultures negative | Died |
| Machata et al.^38^  *2005* | 40 | M | Trauma head injury, fractures of the fifth cervical vertebra + 6 weeks after trauma: septic shock – cause of death (multiresistant *Pseudomonas aeruginosa*) | A+S | 3 (during sedation) | 3 (during sedation) | ˃ 72 hours | Fever 41°C, darkened urine, anuria, renal insufficiency | Serum creatine kinase 708.04 nmol/L, serum myoglobin 4625.1 nmol/L, creatinine 0.48 mmol/l, hyperkalemia 5.9 mmol/L, metabolic acidosis | NA | NA | Continuous veno-venous hemofiltration, dantrolene; Serum  potassium and body temperature could be normalized  rapidly under hemofiltration. Serum myoglobin and CK also  decreased) | Chest X-ray: no infiltration; microbiology: no infection in blood cultures; microscopic examination of skeletal muscle: muscle fibers with signs of vacuole formation and cytochrome-oxidase-negative fibers; Biochemical examination of the muscle fibers: an increase in free carnitine and NADH-CoQ-oxidoreductase (may be an indicator for a metabolic myopathy) | Died (later septic complications) |
| Suen et al.^39^  *2006* | 31 | M | Thoracoscopic wedge resection (lung biopsy of suspicious tumor) + minithoracotomy → adult respiratory distress syndrome | S | NA (range 7.5-13.75) | 13.75 | 5 days | Greenish color urine, arrhythmia, hypotension, acute renal failure, anuria, fever, cardiovascular collapse | Urinanalysis: no hemoglobin, no infection; elevation of total CK 257.500 U/L, CK-MB 156.2 ng/mL, troponin-T 0,09 ng/mL, severe metabolic acidosis (elevated lactate), hyperkalemia, creatinine 3.7 mg/dL, calcium 1.13 mmol/l, serum glutamic pyruvic transaminase 304 U/L, serum glutamic oxaloacetic transaminase 1094 U/L, phosphorus 7.76 mmol/L | Severe ST-segment elevation → ventricular tachycardia and fibrillation → junctional rhythm, widened QRS complex and bradycardia | NA | Resuscitation, cardioversion, transvenous pacing, inotropics, fluid, diuretics, sodium bicarbonate, dantrolene | Chest radiography: new right upper zone infiltrate → increasing bilateral ground  glass opacities.; echocardiography: normal cardiac function; Emergency cardiac catheterization: insignificant coronary artery disease; Histology: a lipemic appearance of the blood, rhabdomyolysis of the diaphragm, quadriceps, and psoas muscles, cardiomegaly, hepatomegaly with steatosis, and splenomegaly | Died |
| Eriksen et al.^40^  *2006* | 20 | F | Osteosynthesis after trauma – multiple fractures – bilateral femoral fractures, fractures of the mandibular and the maxilla, a minor pneumothorax and a slight contusion of both lungs; fractures of the dens axis, the right occipital condyle, and the right articular process of the 4^th^ cervical vertebra suspected | S (not during A) | 4.5-5.1mg/kg/h | NA  5.1?? | 5 days | Green color of urine, hemodynamic instability, no signs of compartment syndrome, rhabdomyolysis, arrhythmia | CK 52.295 IU/l, myoglobine 1030 µg/l, CK-B 18 IU/l, troponin 3.1 µg/l, creatinine 158 µmol/l, hyperkalemia, no metabolic acidosis, normal liver, pancreas and coagulation blood samples | Ventricular tachycardia | Dopamine 6 mg/kg/min | Sodium bicarbonate, dopamine, norepinephrine, forced alkalized diuresis, furosemide, lidocaine, amiodarone | Initial brain scan: normal; chest X-ray: normal; microbiology: pulmonary secretion, blood and urine: normal; Later CT scan of the head, thorax and abdomen: a superficial intracerebral bleeding and slight cerebral oedema, bilateral basal atelectasis of the lungs; echocardiography: no pericardial effusion; Histology: a normal-sized brain with increased volume of the gyri and three superficial intracerebral bleedings in the grey matter, normal-sized lungs without contusion, blood or secretion, but with a firm consistence, normal heart, liver, pancreas and spleen; normal kidneys, but a little pale with increased fluid content; no necrosis of myocardium, but a slight interstitial and perivascular fibrosis and a small bleeding in the tissure; no fatty infiltration of the liver; Toxicology: traces of propofol in the liver tissue ; cause of death: multi-organ failure | Died |
| Vernooy et al.^41^  *2006* | 15 | M | Head injury | S | 4.5mg/kg/hour | NA | 6days | Rhabdomyolysis, fever, arrhythmia | Myoglobinuria, creatine kinase 92,300U/L, creatinine 265µmol/L | ST-segment elevation in the right precordial leads (Brugada like ECG patern) and inverted T waves; after withdrawal of propofol ST segment elevations normalized over the following hours, inversion of T waves persisted; no evidence of arrhythmia | Concomitant administration of dopamine, norepinephrine; (to maintain cerebral perfusion pressure) | Early withdrawal of propofol infusion | Screening of DNA mutation performed (SCN5A^8^) → inherited form of ST-segment elevation excluded | Survived |
|  | 17 | F | Head injury | S | 7.3 | NA | ˃58 hours | Fever, arrhythmia | Hyperkalemia ˃5.5mmol/l | Brugada-like ECG pattern →ventricular tachyarrhythmia →ventricular fibrillation |  | - | - | Died |
|  | 44 | M | Head injury | S | 5.7 | NA | ˃58 hours | Fever, severe hypotension, arrhythmia | - | Supraventricular tachycardia with poor ventricular contractility |  | Ventricular pacing | - | Died |
|  | 17 | M | Head injury | S | 6.6 | NA | ˃58 hours | Arrhythmia | - | Brugada-like ECG pattern →ventricular tachyarrhythmia →ventricular fibrillation |  | - | - | Died |
|  | 20 | F | Head injury | S | 5.5 | NA | ˃58 hours | Arrhythmia | Metabolic acidosis | Brugada-like ECG pattern →ventricular tachyarrhythmia →ventricular fibrillation |  | - | - | Died |
|  | 24 | M | Head injury | S | 7.4 | NA | ˃58 hours | Arrhythmia | - | Brugada-like ECG pattern →ventricular tachyarrhythmia →ventricular fibrillation |  | - | - | Died |
|  | 40 | M | Head injury | S | 5.8 | NA | ˃58 hours | Fever, arrhythmia | Hyperkalemia ˃5.5mmol/l | Brugada-like ECG pattern →ventricular tachyarrhythmia →ventricular fibrillation |  | - | - | Died |
|  | 31 | F | Head injury | S | 6.9 | NA | ˃58 hours | Arrhythmia | Metabolic acidosis, hyperkalemia ˃5.5mmol/l | Brugada-like ECG pattern →ventricular tachyarrhythmia →ventricular fibrillation |  | - | - | Died |
| Merz et al.^42^  *2006* | 24 | M | Spinal injury, aspiration, ARDS, SIRS | S | 1.9 | 2.6 | ˂4 days | Acute renal failure, rhabdomyolysis, massive cerebral edema, arrhythmia | Increased cardiac troponin I 6,3ng/mL and creatine kinase 486,900U/L, hyperkalemia | Bradycardia | Concomitant use of large-dose methylprednisolone + moderate adrenergic support (dopamine, norepinephrine) | NA | NA | Died |
| Corbett et al.^43^  *2006* | 21 | M | Traumatic brain injury | S | 1.9-12 | 12 | 3 days | Oliguria, transient renal insufficiency, arrhythmia | Metabolic acidosis (lactic level 10.9mmol/l), creatinine 1.3mg/dL, creatinine kinase 3076µ/L | Sinus tachycardia → normal sinus rhythm | Concomitant use of high-dose vasopressor therapy (dopamine, norepinephrine, phenylephrine) (to maintain CPP) | Therapy with metoprolol, captopril, early withdrawal of propofol | CT of the head: a comminuted  depressed skull fracture of the  right frontal bone, with a subdural hematoma  and interventricular hemorrhage; plain  films of the chest and pelvis and computed  tomography of the abdomen and  pelvis: negative; Echocardiogram: severe global left ventricular dysfunction and moderate global dysfunction of the right ventricle → resolution of cardiomyopathy | Survived |
| Hermanns et al.^44^  *2006* | 16 | F | Scoliosis surgery (in patient with neonatal progeroid syndrome) | A | 9.1 | NA | ˃6.5 h | Darkish urine, hypotension, arrhythmia | Urine myoglobin concentration 1660µg/l, metabolic (lactic) acidosis | Tachycardia → atrioventricular nodal rhythm (nodal tachycardia) → tertiary AV block with a ventricular heart rate of 10-20 beats/min → sinus rhythm | Concomitant use of norepinephrine | Therapy by verapamil → epinephrine, atropine, orciprenaline | NA | Survived |
| Tramptisch et al.^45^  *2006* | 66 | NA | Postoperative period after aortocoronary bypass | S | 1-3 mg/kg/h with boluses | 3 | 9 days | Hepatomegaly?, arrhythmia | ↑ lactate 4.1 mmol/l, myoglobin 12.467 µg/ml, creatin kinase 27.580 U/l, serum creatinine 2.64 mg/dl, kalium 5,4 mmol/l | Paroxysmal atrial fibrillation | Noradrenalin, dobutamin | Discontinuation of propofol | CT: lesion in the periventricular  medullary and hepatomegaly | Survived |
| Chukwuemeka et al.^46^  *2006* | 45 | M | Coronary artery bypass grafting | A+S | 44µg/kg/min + 80 mg bolus | 44µg/kg/min | ˂ 10 hours | Hypotension, arrhythmia | Metabolic (lactic) acidosis | Sinus tachycardia with widespread ST segment elevation → ventricular tachycardia | Dopamine | Fluid resuscitation, intravenous calcium chloride, adrenaline, electrical direct current cardioversion, amiodarone, epinephrine, hydrocortisone, diphenhydramine, sodium bicarbonate; withdrawal of propofol | Echocardiography: good left ventricular, no cardiac valvular pathology; → transoesophageal echocardiography: good biventricular contractility and adequate ventricular filling; | Survived |
| De Waele et al.^47^  *2006* | 30 | M | After laparotomy for drainage of an abdominal abscess (quadriplegic patient) + bilateral *Pseudomonas aeruginosa* pneumonia | S | 5.7 (2%) | 5.7 | 56 hours | Arrhythmia | Increased troponin T (0,16ng/ml), creatine phosphokinase 9008IU/l, lactate (19mg/dl) | Sinus arrhythmias and intermittent atrial fibrillation, inferolateral T-wave inversion → cardiac arrhythmias disappeared after discontinuation of propofol infusion | Concomitant use of norepinephrine | Withdrawal of propofol | Transthoracic echocardiography: normal, no pericardial effusion; Microbiology: bilateral *Pseudomonas aeruginosa* pneumonia | Survived |
| Sabsovich et al.^48^  *2007* | 16 | M | Traumatic brain injury | S | 1.66-8.33 | 8.33 | 3 days | Rusty brown urine, acute renal failure, hypotension, arrhythmia | Creatinine 371µmol/L, Metabolic acidosis (pH 7.1), bicarbonate 10mmol/L, CPK 251762U/L, aspartate aminotransferase 3082U/L, alanine aminotransferase 1144U/L, lactate dehydrogenase 4687U/L, MB fraction of CPK 333.5U/L, troponin 17.39ng/mL | Left bundle branch block with diffuse changes in the ST segment and the T wave → new onset of diffuse changes in the ST segment and T wave → wide-complex tachycardia → bradycardia → asystole | Concomitant use of phenylephrine | Treatment by aggressive hydration, bicarbonate infusion, fluid resuscitation; discontinuation of propofol; | Echocardiography: normal myocardial infusion | Died |
| Westhout et al.^49^  *2007* | 3 | F | Angiographic embolization (aneurysm in the left internal carotid artery) | A + S | Bolus of 80 mf propofol (for 3 hours) + 200 µg/kg/hr (4 hours) | 200 µg/kg/hr | 8 hours | Hypotension, arrhythmia | Metabolic acidosis, CK 591 U/L, lactate dehydrogenase 179 U/L, lactate 1.1 mmol/L; the blood urea nitrogen 11 mg/dl, creatinine 0.6 mg/dl, normal salicylate and ammonia; a creatine phosphokinase  2 - myoglobin level of 20.7 ng/ml  and a relative index of 3.5  . | Tachycardia | NA | Bicarbonate, aggressive fluid resuscitation with 5% albumin, vasopressors, 5 mg dexamethasone | NA | Survived |
| Zarovnaya et al.^50^  *2007* | 36 | F | Status epilepticus | S | 4.2-7.2 | 7.2 | 64 hours | Dark urine, fever, hypotension, decreasing urine output, arrhythmia | CPK 150.000U/L, metabolic (lactic) acidosis, elevated serum creatinine | Pulseless electrical activity → asystole followed by wide complex tachycardia → diffuse low voltage, diffuse T-wave flattening, normal QT → wide QRS complexes → bradyarrhythmia → asystole | NA | Treatment by aggressive resuscitation including defibrillation, pacing, hemodialysis, phenylephrine, vasopressin, dobutamine | Biopsy of skeletal muscle: acute aseptic necrosis, consistent with rhabdomyolysis; myocyte swelling, loss of striation, vacuole formation, interstitial edema, variable myocyte hypertrophy; red myoglobin casts in the dilated tubules; congestion of the lungs, liver and spleen  Echocardiogram: global biventricular dysfunction | Died |
| Karakitsos et al.^51^  *2007* | 35 | F | Head injury | S | 25-30ml/h (2% propofol) | 30ml/h | 4 days | Acute renal failure with concomitant myocardial dysfunction | Metabolic acidosis, hyperkalaemia 6.2mmol/l, creatine kinase 42.000U/l, myoglobin 2100µg/l, lipaemia 4.1mmol/l | NA | Concomitant use of vasopressors | Therapy by haemofiltration, early withdrawal of propofol | Transoesophageal echocardiography: global left ventricular hypokinesia | Survived |
| Rosen et al.^52^  *2007* | 18 | M | Trauma brain injury | S | NA | 7,5 | ˃72 hours | Dark urine, anuria, worsening hemodynamics, arrhythmia | Creatine phophokinase 95.440U/L, serum creatinine 3.6g/dL, metabolic acidosis pH 7.1 | Cardiac arrhythmias → asystole | Low-dose vasopressors | Resuscitation | NA | Died |
|  | 29 | F | Trauma brain injury | S | 4-12 | 12 | 6 days | Hypotension, cardiovascular collapse | Metabolic (lactic) acidosis 7.01, CPK 6.966U/L, myoglobinuria | Cardiovascular collapse | Concomitant use of epinephrine | Treatment by discontinuation of propofol (with thiopental), phenylephrine, aggressive intravascular volume support | NA | Died |
| Bordes et al.^53^  *2008* | 66 | NA | Glioblastoma | A | 6.9 | 7.6 | 5 hours | NA | Hyperlactataemia | NA | Concomitant use of steroids | Discontinuation of propofol infusion | NA | Survived |
| Fudickar et al.^54^  *2008* | 21 | F | Cerebellar bleeding + surgical decompression of the occipital cranium | S + A | 5.7 mg/kg/hour (2%) | 5.7 | 5 days | Arrhythmia | CK 5194 U/L, CK-MM 29424 U/L, myoglobin 4880 µg/L, normal lactate, sodium bicarbonate and pH, increased AST and ALT, GGT, amylase and lipase, normal bilirubin, serum creatinine, serum urea and urinary output | Ventricular ectopic beats and ST-segment depression in leads II, III and aVF; | Concomitant use of norepinephrine. hydrocortisone | Discontinuation of propofol and replacement by midazolam, amiodarone | Digital subtraction angiography: arteriovenous malformation of the brain stem; Ultrasound liver scan: no signs of liver damage; Brain magnetic resonance imaging (MRI): no ischaemic damage; Brain stam acoustic evoked potentials and somatosensory evoked potentials: normal | Survived |
| Romero et al.^55^  *2008* | 43 | F | Brain surgery due to a vascular malformation | A+S | 3.5-7 | 7 | 15 hours (7 hours anaesthesia, 8 hours sedation) | NA | Metabolic (lactic) acidosis | ECG without abnormalities | Concomitant use of catecholamines and corticosteroids | Discontinuation of propofol, treatment of norepinephrine, fluid resuscitation | NA | Survived |
| Shimony et al.^56^  *2008* | 52 | M | Cardiac arrest – stenting of an occluded proximal left anterior descending coronary artery | S | NA | 400mg/hour | 7 days | Acute renal failure, rhabdomyolysis | Hyperbilirubinemia, elevated liver enzymes (alanine aminotransferase 206U/L, aspartate aminotransferase 828U/L), creatine kinase 42.700, creatinine 7.6mg/dl, metabolic acidosis, hyperlipaemia (triglycerides 3050mg/dl) | Initially: anterior wall ST elevation | NA | Discontinuation of propofol and replacement by midazolam, hemodiafiltration | Initial echocardiography: severe left ventricular dysfunction | Survived |
| Robinson et al.^57^  *2008* | 9 months | M | Status epilepticus | S | Bolus + 14mg/kg/hour | 14 | ˂ 3days | Arrhythmia | CK 21.000U/L, mild metabolic acidosis (bicarbonate 15mmol/L, lactate 3.5mmol/L), creatine kinase muscle brain isoform (CKMB) relative index 3.1%, troponin T 0.03ng/mL | Slow sinus mechanism with low-amplitude P waves, first-degree atrioventricular block, low ventricular voltages, a slightly widened QRS complex consistent with right ventricular conduction delay, nonspecific ST and T-wave changes, escape beats with a slightly different QRS morphology → progression of the intraventricular conduction delay to complete right-bundle-branch block → diffuse abnormalities (markedly low voltages and irregular QRS pattern with complete right-bundle-branch block), AV conduction with first and second degree AV block → sustained wide-QRS tachycardia with left-bundle-branch-block morphology → after cardioversion: irregular pattern with the baseline QRS morphology of the right-bundle-branch block with some premature beats and short salvos of tachycardia with a left-bundle-branch block QRS morphology; diffuse conduction disturbances complicated by ventricular tachycardia of right-ventricular origin with a left-bundle-branch block QRS morphology; Lidocaine infusion: episods of ventricular tachycardia; sinus rhythm with improved voltages and less intraventricular conduction delay, dramatic ST elevation in lead V1 in a pattern similar to Brugada syndrome → ECG normalized | NA | Stopping propofol; Treatment by lidocaine, adenosine, atrial overdrive pacing via the esophageal electrode, synchronized direct current cardioversion with 1.5 J/kg, lidocaine infusion | Echocardiography: normal biventricular function and no structural defects` Cranial magnetic resonance  imaging revealed a mild Chiari I malformation  and changes in the right thalamus consistent with  prolonged seizure activity, but no major pathology. | Survived |
| Zaccheo et al.^58^  *2008* | 33 | F | Trauma head injury | S | 20-190µg/kg/min | 190µg/kg/min | 6 days | Hypotension, arrhythmia | Serum triglyceride level 11.420mg/dL, metabolic acidosis (pH 7.17, bicarbonate 8.9mEq/L, lactic acid 12.5mg/dL), total creatine kinase 18.333U/L, myoglobin 18.470ng/mL | Normal sinus rhythm with a first-degree atrioventricular block, left anterior hemiblock, right bundle branch block → wide-complex ventricular rhythm → tachycardic and bradycardic rhythms → cardiac arrest with pulseless electrical activity | Concomitant use of norepinephrine | Treatment by fluid replacement and norepinephrine, epinephrine, sodium bicarbonate, transvenous pacemaker, discontinuation of propofol | Computed tomography of  the head showed a right temporal  subdural hematoma and a subarachnoid  hemorrhage. | Died |
|  | 64 | M | Status epilepticus (alcohol withdrawal seizures) | S | Bolus of 1mg/kg + infusion of 120-140µg/kg/min | 140µg/kg/min | 48 hours | Hypotension, acute oliguric renal failure, shock, rhabdomyolysis | Metabolic acidosis (lactic acid 8.4mg/dL pH 7.34, bicarbonate 14.1mEq/L), myoglobin 210.030ng/mL, CK 69.175U/L, serum triglyceride 628mg/dL | NA | Norepinephrine | Crystalloid/colloid volume replacement, norepinephrine, continuous renal replacement therapy | NA | Died |
| Aloizos et al.^59^  *2008* | 20 | M | Upper airway obstruction due to Epstein-Barr infection | S | 2.14-5.71 | 5.71 | 4 days | Rhabdomyolysis, acute renal failure with oliguria progressing to complete anuria → polyuria | CPK ˃7000iu/lt, creatinine 7mg/dl, no sign of lactic acidosis | NA (without a signs of heart failure) | NA | Hydration, bicarbonate administration, replacement of propofol with midazolam, continuous veno-venous haemodiafiltration dialysis | Bed side abdominal ultrasonography: mild liver and spleen enlargement; Microbiology: blood, urine and broncho alveolar lavage (BAL) cultures negative.  Abdominal  computed tomography (CT) findings: consistent  with the EBV infection. | Survived |
| Laquay et al.^60^  *2008* | 12 | F | Surgical correction of congenital mitral regurgitation on a mitral cleft with normothermic cardiopulmonary bypass (CPB) | A | ˂3mg | ˂3mg | 15 hours | NA | Metabolic acidosis (lactate 9.3mmol/l) | NA | Concomitant use of epinephrine | Treatment by sodium bicarbonate, early cessation of propofol infusion | Transoesophageal echocardiography at the end of surgery: a balanced contractility of the two ventricles and the lack of residual mitral regurgitation | Survived |
|  | 16 | F | Surgical correction of congenital mitral regurgitation on a mitral cleft with normothermic cardiopulmonary bypass (CPB) | A | ˂3mg | ˂3mg | 8 hours | NA | Metabolic acidosis (lactate 14.7mmol/l) | NA | Concomitant use of epinephrine | Treatment by sodium bicarbonate, early cessation of propofol infusion | Transoesophageal echocardiography at the end of surgery: a balanced contractility of the two ventricles and the lack of residual mitral regurgitation | Survived |
| Smith et al.^61^  *2009* | 28 | M | Trauma brain injury | A+S | A:50-75mcg/kg/min  S: 95-125mcg/kg/min | 125mcg/kg/min | ˃85 hours | Cardiac complications | Mild metabolic acidosis, CK 12.858U/l, CK-MB 59.5ng/ml, LDH 618U/l, troponin 0.9ng/ml | T-wave inversion and prolonged QTc intervsl | Concomitant use of phenylephrine | Treatment by dobutamine, replacement of propofol with a midazolam | Echocardiogram: a left ventricular ejection fraction of 40-45% | Survived |
|  | 38 | M | Trauma brain injury | S | 75-125mcg/kg/min | 125mcg/kg/min | 89 hours | Arrhythmia | CK 5.111U/l, CK-MB 30.9ng/ml, LDH 409U/l | ST elevation and tachyarrhythmias → polymorphic ventricular tachycardia → spontaneously reverted to normal waveform morphology 12 h after stopping propofol | Concomitant use of phenylephrine, dopamine | Treatment by replacement of propofol with a thiopental infusion | Echocardiogram: preserved left ventricular ejection fraction of 55-60% | Survived |
|  | 25 | M | Trauma brain injury | S | 30-134mcg/kg/min | 134mcg/kg/min | 135 hours | NA | CK ˃25.300U/l, metabolic acidosis, CK-MB 17.8ng/ml, troponin 0.7ng/ml, LDH 1.098U/l, positive urine myoglobin | Inverted T waves | Concomitant use of phenylephrine | Treatment by replacement of propofol with a pentobarbital infusion | NA | Died |
| Ilyas et al.^62^  *2009* | 67 | M | Coronary artery bypass grafting (CABG) using cardiopulmonary bypass + atrial fibrillation | A | 0.8-5.2 | 5.2 | 7,5 hours | NA | Metabolic acidosis (lactic acid 13.3 mmol/L), myoglobinuria, hemoglobinuria, no ketonuria, creatine kinase 260 mmol/L, with  predominant skeletal muscle expressed fraction of creatine  kinase CK-MM, normal troponin, liver enzymes | No electrocardiographic abnormalities | Concomitant use of epinephrine; Preoperative medications: prednisolone | Discontinuation of propofol infusion | Initial transoesophageal echocardiography: mild left ventricular dysfunction; | Survived |
| Veldhoen et al.^63^  *2009* | 17 | M | Multiple skull fractures – trauma | S | NA (range 3-8 mg/kg/h) | Max. 8 mg/kg/hrfor a total of  14 hours | 4 days | Oliguria → progressive cardiac failure with anuria and rhabdomyolysis, arrhythmia | Progressive lactic acidosis, ↑ CK 7476 U/L; ↑ acylcarnitine C2 22.9 µmol/L,  and ↑ C4 0.46  µmol/L | Tachycardia, cardiac failure | Ionotropic support | Discontinuation of propofol, high carbohydrate intake, high doses of inotropic support, repeated resuscitation; mechanical cardiac support with intra-aortic balloon pump: failed because of tachycardia | Initial  computerized tomography scan of the  brain: no abnormalities → generalized edema of the brain; Microbiology: blood cultures negative; Histology: cerebral edema, no signs of pneumonia; Microscopy of the muscles: no features of degeneration, but enzymatic and histochemical abnormalities of acute muscle degeneration present, mild liver steatosis, contraction band necrosis in myocardial cells | Died |
| Orsini et al.^64^  *2009* | 36 | F | Respiratory failure and sepsis, likely secondary to pneumonia | S | 3.5-6 | 6 | 8 days | Morbilliformrash on the neck, shoulders, chest, dark-green urine, hepatomegaly, arrhythmia | Hypertriglyceridemia 1.005mg/dL, increased amylase 294U/L, lipase 608U/L, creatine kinase levels 36.327U/L, abnormal liver function test values (AST 115U/L,ALT 536U/L, γGT 501IU/L), pH 7.42, normal troponin level | Sinus tachycardia | Concomitant use of norepinephrine, vasopressin, hydrocortisone; | Discontinuation of propofol | Computed tomography of abdomen: hepatomegaly with fatty infiltration of liver; Transthoracic echocardiogram: normal left ventricular function | Survived |
| Iyer et al.^65^  *2009* | NA (range: 31-77) | M | Status epilepticus | S | NA (range: 10-118µg/kg/min) | NA (range:50-200 µg/kg/min) | NA (range: 36-391 hours) | NA (occurrence of one or more of the following: metabolic acidosis, rhabdomyolysis, bradycardia, heart failure, hyperkalemia, renal failure, lipemia, other arrhythmias and death) | NA | Without cardiac arrest | NA | NA | NA | Survived |
|  | NA (range: 31-77) | M | Status epilepticus | S | NA (range: 10-118µg/kg/min) | NA (range:50-200 µg/kg/min) | NA (range: 36-391 hours) | NA (occurrence of one or more of the following: metabolic acidosis, rhabdomyolysis, bradycardia, heart failure, hyperkalemia, renal failure, lipemia, other arrhythmias and death) | NA | Without cardiac arrest | NA | NA | NA | Survived |
|  | NA (range: 31-77) | M | Status epilepticus | S | NA (range: 10-118µg/kg/min) | NA (range:50-200 µg/kg/min) | NA (range: 36-391 hours) | NA (occurrence of one or more of the following: metabolic acidosis, rhabdomyolysis, bradycardia, heart failure, hyperkalemia, renal failure, lipemia, other arrhythmias and death) | NA | Without cardiac arrest | NA | NA | NA | Survived |
|  | NA (range: 31-77) | M | Status epilepticus | S | NA (range: 10-118µg/kg/min) | NA (range:50-200 µg/kg/min) | NA (range: 36-391 hours) | NA (occurrence of one or more of the following: metabolic acidosis, rhabdomyolysis, bradycardia, heart failure, hyperkalemia, renal failure, lipemia, other arrhythmias and death) | NA | Without cardiac arrest | NA | NA | NA | Survived |
|  | NA (range: 31-77) | M | Status epilepticus | S | NA (range: 10-118µg/kg/min) | NA (range:50-200 µg/kg/min) | NA (range: 36-391 hours) | NA (occurrence of one or more of the following: metabolic acidosis, rhabdomyolysis, bradycardia, heart failure, hyperkalemia, renal failure, lipemia, other arrhythmias and death) | NA | Without cardiac arrest | NA | NA | NA | Survived |
|  | NA (range: 31-77) | M | Status epilepticus | S | NA (range: 10-118µg/kg/min) | NA (range:50-200 µg/kg/min) | NA (range: 36-391 hours) | NA (occurrence of one or more of the following: metabolic acidosis, rhabdomyolysis, bradycardia, heart failure, hyperkalemia, renal failure, lipemia, other arrhythmias and death) | NA | Without cardiac arrest | NA | NA | NA | Survived |
|  | NA (range: 31-77) | M | Status epilepticus | S | NA (range: 10-118µg/kg/min) | NA (range:50-200 µg/kg/min) | NA (range: 36-391 hours) | NA (occurrence of one or more of the following: metabolic acidosis, rhabdomyolysis, bradycardia, heart failure, hyperkalemia, renal failure, lipemia, other arrhythmias and death) | NA | Without cardiac arrest | NA | NA | NA | Survived |
|  | NA (range: 31-77) | F | Status epilepticus | S | NA (range: 10-118µg/kg/min) | NA (range:50-200 µg/kg/min) | NA (range: 36-391 hours) | NA (occurrence of one or more of the following: metabolic acidosis, rhabdomyolysis, bradycardia, heart failure, hyperkalemia, renal failure, lipemia, other arrhythmias and death) | NA | Without cardiac arrest | NA | NA | NA | Survived |
|  | NA (range: 31-77) | F | Status epilepticus | S | NA (range: 10-118µg/kg/min) | NA (range:50-200 µg/kg/min) | NA (range: 36-391 hours) | NA (occurrence of one or more of the following: metabolic acidosis, rhabdomyolysis, bradycardia, heart failure, hyperkalemia, renal failure, lipemia, other arrhythmias and death) | NA | Without cardiac arrest | NA | NA | NA | Survived |
|  | NA (range: 31-77) | F | Status epilepticus | S | NA (range: 10-118µg/kg/min) | NA (range:50-200 µg/kg/min) | NA (range: 36-391 hours) | NA (occurrence of one or more of the following: metabolic acidosis, rhabdomyolysis, bradycardia, heart failure, hyperkalemia, renal failure, lipemia, other arrhythmias and death) | NA | Without cardiac arrest | NA | NA | NA | Survived |
|  | NA (range: 31-77) | F | Status epilepticus | S | NA (range: 10-118µg/kg/min) | NA (range:50-200 µg/kg/min) | NA (range: 36-391 hours) | NA (occurrence of one or more of the following: metabolic acidosis, rhabdomyolysis, bradycardia, heart failure, hyperkalemia, renal failure, lipemia, other arrhythmias and death) | NA | Without cardiac arrest | NA | NA | NA | Survived |
|  | 37 | M | Status epilepticus | S | 140µg/kg/min | 140µg/kg/min | 4 days | Fever 38,4°C, diminished urine output, hypotension, arrhythmia | Metabolic acidosis (lactate 2.4mmol/L), elevated ALT and AST values, myoglobinuria | Tachycardia → widened QRS complex (QTc 549 msecs), right bundle branch block, junctional rhythm, bradycardia → ventricular tachycardia → ventricular fibrillation → asystolic cardiac arrest | NA | Aggressive fluid and pressor resuscitation | Microbiology: Blood cultures obtained on arrival  negative. | Died |
|  | 46 | F | Status epilepticus | S | 70-175µg/kg/min | 175µg/kg/min | 66 hours | Hypotension, arrhythmia | Metabolic acidosis (pH 7.26), elevated CK 3.538U/L, elevated liver function tests (ALT 94U/L, AST 187U/L), elevated triglyceride 720mg/dL | Incomplete right bundle branch block with a prolonged QTc (546msesc) along with T-wave inversions in the inferior limb leads and lateral precordial leads → a markedly changed rhythm with complete right bundle branch block, bradycardia, QRS prolongation, junctional rhythm, greatly magnified T-wave inversions → wide, complex, pulseless electrical state | NA | Treatment by phenylephrine, norepinephrine, epinephrine treatment, immediately stopping of propofol | Echocardiogram: normal sized right and left ventricles and a calculated LVEF of 30% | Died |
|  | 55 | F | Status epilepticus | S | 17-110µg/kg/min | 110µg/kg/min | ˃ 3.5 days | Hypontesion, arrhythmia | NA | Sinus bradycardia, a very prolonged QTc interval (720msecs), and widespread T-wave inversion → a wide complex tachycardia resembling torsades de pointes | NA | Stopping of propofol and replacement by midazolam, cardiopulmonary resuscitation with magnesium and an advanced cardiac life support protocol | NA | Survived |
| Mali et al.^66^  *2009* | 48 | F | Thoracoscopic esophagectomy for carcinoma of the esophagus | A | 3 (+bolus of 1mg/kg/hour) | 3 | 4 hours | Arrhythmia | Serum potassium 8.2mmol/L, pH 7.33 (not considered as acidosis by authors), myoglobinuria | Tall T waves, flattened P waves, widened QRS complex, episode of bradycardia → taller T waves, wide QRS complex, absent P waves → QRS widening decreased | NA | Treatment by atropine, calcium gluconate, sodium bicarbonate, glucose with insulin | NA | Survived |
| Blum et al.^67^  *2009* | 40 | M | A type B aortic dissection (after stenting of the superior mesenteric, renal, and iliac arteries) | S | 50-90µg/kg/min | 90µg/kg/min | ˃5 days | Arrhythmia | Slightly elevated lactate without acidosis, elevated CK 18.902IU/L, urinary myoglobin 66IU/L, troponin 51.6ng/ml | Elevated ST segments throughout → multiple periods of unstable ventricular tachycardia (requiring ACLS medications and defibrillation), after pacing: dramatically improved ECG showing ST elevations in leads V1-V3 → later normal | NA | Discontinuation of propofol, transvenous pacer | Transoesophageal echocardiography: normal function, left ventricular hypertrophy, mild pericardial effusion, no evidence of retrograde dissection → an ejection fraction of 40%; coronary angiogram: no abnormality | Survived |
| Jorens et al.^68^  *2009* | 12 | M | Traumatic cerebral edema, epidural hematoma | S | NA | NA | 5 days | Rhabdomyolysis, arrhythmia | CK 863.000IU/L, elevated cardiac troponin levels, lactic acidosis, lipemic serum with a triglyceride level of 591ng/ml | A sudden nodal bradyarrhythmia with coved-type ST-segment elevation in the right precordial leads → ventricular arrhythmias | Concomitant use of catecholamines | Bicarbonate administration, dialysis, cardiac extracorporeal support | Autopsy: an accumulation of fat droplets and acute vacuolar degeneration and myocytolysis of skeletal and cardiac muscle; massive reddish brown, myoglobin-immunoreactivepigment casts in the renal tubular lumina | Died |
| Weiner et al.^69^  *2009* | 21 | M | Drug intoxication (alprazolam, tramadol) | S | 25mcg/kg/min | 25mcg/kg/min | ˃14 hours | Arrthythmia | Mixed acidosis (pH 7.14), potassium 5.4mEq/L, creatinine 2.34mg/dl, CPK 3.269 U/L, troponin-I 2.82 ng/mL | RSR’ pattern with mild ST elevation in V1-V2 → a pronounced Type I Brugada pattern with coved type ST segment elevations in V1-V2, anteroseptal leads → after stopping propofol: resolution of the Brugada pattern | Concomitant use of dopamine | Discontinuation of propofol | Coronary angiography: no obstructive coronary disease; a cardiac magnetic resonance: normal LV and RV function with no structural abnormalities | Survived |
| Roberts et al.^70^  *2009* | 11 patients at the age of: 58 ± 14 | 82% males, 18% females | Critically ill patients | S | NA | NA | 5 (3-7) | Renal failure, (11 patients), cardiac dysfunction (11 patients) | Metabolic acidosis (11 patients), hypertriglyceridemia (1) | NA | Catecholamines (10 patients) | NA | NA | 2 of the patients died |
| Da-Silva et al.^71^  *2010* | 4 | M | Malignant status epilepticus associated with bacterial meningitis | S | 0.6-15.6 | 15.6 | 6 days | Dark green urine, hypotension, rhabdomyolysis, arrhythmia | Metabolic acidosis (pH 7.32, bicarbonates 15mEq/L), CK 155.400U/L, CK myoglobin 2.317ng/mL, troponin T 0.10µg/L, triglycerides 5160mg/dL | Intermitent episodes of nonsinus bradycardia → idioventricular vs.junctional rhythm, increased QTc, and ventricular escape → after transfusion and CVVH: sinus rhythm | NA | Discontinuation of propofol, treatment by dopamine, calcium gluconate, sodium bicarbonate; manual exchange transfusion with 600mL of packed red blood cells (PRBCs) by using alternate push-pull method (PECT: Parcial-exchange blood transfusion) followed by CVVH | Echocardiography: normal shortening fractions of 38.9%, resp. 33.6% | Survived |
| Guitton et al.^72^  *2010* | 17 | F | Refractory status epilepticus, with aseptic meningoencephalitis | S | NA | 8.8 | 58 hours | Arterial hypotension, renal failure, rhabdomyolysis, cardiocirculatory failure, arrhythmia | Metabolic acidosis ( pH 7.24, lactate 7.2mmol/L, bicarbonate 12.2mmol/L), altered renal function (potassium 5.3mmol/L, urea 13.7mg/dL, creatinine 2.7mg/dL),myoglobin˃30.000µg/L, CPK 168.000U/L, increased troponin, AST 1.577U/L, ALT 245U/L | Bradycardia → prolonged QRS → normal QRS with sinus rhythm → QRS waves gradually widened → cardiocirculatory arrest with refractory ventricular fibrillation → after ECMO: correction of rhythmic conduction | Because of hypotension norepinephrine | Fluid challenge and norepinephrine, calcium gluconate, molar lactate infusions, bicarbonates, veno-venous hemofiltration, followed by extracorporeal membrane oxygenation (ECMO) | Chest X-ray: normal; Brain computed tomography (with intravenous contrast): normal. Analysis of cerebrospinal fluid (CSF):  raised protein (1.24 g/L), three leukocytes/mL, and no  bacteria (a diagnosis of aseptic meningoencephalitis  suspected). echocardiography: normal, without any changes in left ventricular function → left ventricular function gradually decreased; Abdominal and hepatic ultrasounds: normal; after ECMO: progressive improvement of left ventricular function | Survived |
| Sammartino et al.^73^  *2010* | 33 PCA preterm baby (born at 24-week gestation) | NA | Laser therapy for retinopathy of prematurity | A | Bolus of 3mg/kg + infusion: 60-80mg/kg/hour | 80mg/kg/hour | 2 hours | Hypotension, hyposaturation | Lipemic serum, AST 1.200UI/l, ALT 5.760UI/l, cholesterol 102mg/dl, triglycerides 2.168mg/dl, K 5.1mEq/l, blood urea nitrogen 33mg/dl | A decrease in heart rate ˂100bpm | NA | Concomitant use of parenteral nutrition (suspended 3 hours before surgery);  Discontinuation of propofol, glucose 10% + NaCl + albumin, dopamine | NA | Survived |
| Soler-Rodenas et al.^74^  *2010* | 20 | F | Traumatic brain injury | S | 2,6-6 | 6 | 5 days | Arrhythmia, rhabdomyolysis, renal insufficience, hypotension, cardiac failure | Hyperkalemia, metabolic acidosis, phosphatemia 3,7 mmol/l, CK 36.204 U/l, myoglobin 590mg/l | Bradycardia → cardiac arrest | Catecholamines and steroids | Noradrenaline, adrenaline | NA | Died |
| Power et al.^75^  *2011* | NA | M | Refractory status epilepticus + viral encephalitis | S | 229,3 mg/h | NA | 107, 5 hours | Cardiac arrhythmia, hypotension and rhabdomyolysis | Respiratory and metabolic acidosis | Cardiac arrhythmia | NA | NA | NA | Died – PRIS cause of death? |
| Amrein et al.^76^  *2011* | 20 | M | Traumatic head injury + bilateral decompressive craniectomy | S | 4.5 mg/kg/h firstly + 1.4 mg/kg h secondly | 4.5 | Firstly: 6 days, after that reexposure for procedure | Hypotension, rhabdomyolysis, arrhythmia | Serum creatine kinase 16.249 IU/L, no metabolic acidosis | Broad complex arrhythmia | Concomitant use of norepinephrine | Aggressive fluid resuscitation | Computed tomography of the brain: bilateral brain contusions and intracerebral hematoma; Transesophageal echocardiogram: diffuse hypokinesia with a left ventricular ejection fraction of 40%, transthoracic echocardiogram: normalization of left and right ventricular function | Survived |
| Diedrich et al.^77^  *2011* | 37 | M | Status epilepticus | S | 1.8-8.4 | 8.4 | ˃5 days | Rhabdomyolysis, fever, hypotension, arrhythmia | Increased CK, myoglobin and serum creatinine, metabolic acidosis (2.4 mmol/L) | Tachycardia → bradycardia with a RBBB → wide complex tachycardia → ventricular tachycardia, fibrillation → asystole | NA | Intravenous hydration, phenylephrine, amiodarone, epinephrine, atropine, calcium, sodium bicarbonate, vasopressin | NA | Died |
|  | 47 | F | Astrocytoma | S | 6-10.5 | 10.5 | ˃8 days | Arrhythmia | Increased creatinine kinase and triglycerides, metabolic acidosis, pH 7.25, bicarbonate 20mmol/L, creatinine kinase 3538U/L | Bradycardia with a RBBB → wide complex pulseless electrical activity → asystole | NA | Phenylephrine; discontinuation of propofol; intravenous calcium, glucagon; atropine, epinephrine, atrial and ventricular pacing | Transthoracic echocardiogram (TTE): generalized hypokinesis, significant decrease in left ventricular function with an ejection fraction (EF) of 30%, | Died |
| Faulkner et al.^78^  *2011* | 23 | M | Refractory status epilepticus (traumatic brain injury and seizure disorder) | S | 4,8 | 4,8 | 5 days | Acute renal failure, severe rhabdomyolysis | Lactic acidosis, hypertriglyceridemia | A typical  type I Brugada pattern characterized by up to 15 mm of high take-off coved ST-segment elevation in leads V1 and V2 → the precordial STsegment  elevation completely resolved | NA | Discontinuation of propofol infusion, aggressive hydration, plasma exchange | Genetic testing negative for several cardiac sodium-channel  and L-type calcium channel abnormalities | Survived |
| Ramaiah et al.^79^  *2011* | 42 | F | Parathyroidectomy, morbid obesity (BMI 75), postoperative respiratory failure secondary to basal atelectasis and ventilator-associated pneumonia, septic shock secondary to urinary tract infection and ventilator-associated pneumonia | A+S | Bolus 300 mg + 20-80 μg/kg/min | 80 μg/kg/min (4 mg/kg/h) | 65 hours | Acute renal failure, rhabdomyolysis, oliguria, | Creatinine kinase (66900 IU/l) and myoglobin (19470 ng/ml) levels started to climb leading to the diagnosis of rhabdomyolysis, increasing creatinine (3.1 mg/dl) and BUN (41 mg/dl), metabolic acidosis with the base deficit of more than 10 mmols/l | NA | Norepinephrine | Propofol infusion replaced with titrating doses of lorazepam and fentanyl for sedation, hemodialysis | NA | Died (cause of death other than PRIS) |
| Testerman et al.^80^  *2011* | 17 | M | Traumatic brain injury | S | From 70 µg/kg/min up | NA | ˃2 days | Hypotension, metabolic acidosis, rhabdomyolysis, acute renal failure, circulatory collapse | Metabolic acidosis | NA | NA | Vasopressors, continuous venovenous hemodialysis | NA | Died |
|  | 40 | M | Head injury | S | 50-90 µg/kg/min | 90 µg/kg/min | 3 days | Arrhythmia | Metabolic acidosis | Bradyarrhythmia | NA | Stopping the propofol infusion, supportive care measurements | NA | Survived |
| Pisapia et al.^81^  *2011* | 37 | F | Aneurysmal subarachnoid hemorrhage+ right frontotemporal craniotomy and microsurgical aneurysm  clipping; respiratory infection | S | ˃4mg/kg/hour | 80 mcg/kg/min (4.8 mg/kg/hour) | ˃48 hours | Rhabdomyolysis, acute renal failure, hypotension, arrhythmia | Metabolic (lactic) acidosis, CPK greater than ˃ 29,000U/l, worsening transaminitis, increased levels of cerebral LPR | Tachycardia, cardiac arrest | Catecholamine vassopressor use | Epinephrine, Norepinephrine, Phenylepinephrine, Vasopressin | A head computed  tomography (CT) scan: subarachnoid hemorrhage, right frontal intraparenchymal  hemorrhage, and subdural blood over the right  convexity → a postoperative head CT the following  day revealed infarction of the inferior right frontal,  temporal, and parietal lobes → an evolving infarct in the distribution of  the right middle cerebral artery; A cerebral angiogram: a 12-mm multilobulated aneurysm arising from the  supraclinoid segment of the right internal carotid artery; Microbiology: *Staphylococcus aureus* in respiratory culture | Died |
| Vanlander et al.^82^  *2012* | 40 | M | Trauma head injury (urgent trepanation and postoperative care) + Leber  hereditary optic neuropathy (LHON) | S | 4.8 mg/kg/h | 5.35 mg/kg/h | 88 hours | Hypotension, rhabdomyolysis, arrhythmia, multiorgan failure, refractory shock | Metabolic (lactic) acidosis, CK ˃ 63711 IU/l, | Nodal bradyarrhythmia with coved ST-T segment elevation in the right precordial leads and atrioventricular dissociation | Noradrenalin | Discontinuation of propofol infusion, renal replacement therapy, supportive drugs (carnitine, thiamine, vitamin B12) with the intention to support mitochondrial function | Computed tomography: multiple hemorrhagic contusions in the left frontoparietal region and an epidural hematoma in the right frontoparietal region → expanding lesions and edema; Histology: congestion of the liver, the lower lobes of the lungs and the brain, atrophy of the optic nerve, widespread myocytolysis in the diaphragm, skeletal and cardiac muscle, and massive accumulation of fat in skeletal muscle fibers; Spectrophotometric analysis of post-mortem skeletal muscle biopsy: a severely deficient complex I activity, with significantly increased activity of other OXPHOS complexes (II, III, IV) and citrate synthase | Died |
| Annecke et al.^83^  *2012* | 36 | F | Severe head injury – a severe bleeding from a midfacial injury | S | NA | 2.8 mg/kg/h | 7 days | Severe shock | Elevated creatine kinase activity 1.800 U/L, metabolic (lactic) acidosis, hyperkalemia 6.0 mmol/L, hyperphosphatemia 7.7 mg/dL, increased ALT and AST, GGT, normal bilirubin | Brugada  syndrome-like electrocardiographic pattern → intermittent episodes of ventricular tachycardia → bradyarrhythmia → episodes of temporary pulsless electrical activity | Norepinephrine | Dobutamine, norepinephrine, hemofiltration, epinephrine, vasopressin, pacer wire introduction | Whole-body computed tomographic scan: an open skull fracture with a small intraparenchymal hematoma of the right temporal lobe,  a traumatic subarachnoid hemorrhage, and multiple complex  facial fractures; moderate contusions of the left  lung, a non-dislocated pelvic fracture, and an open fracture of  the ankle joint also were noted; there was no evidence of  compartment syndromes → no hematoma growth but moderate diffuse  brain swelling, again with no indication for neurosurgical  intervention | Died |
| Richter et al.^84^  *2012* | 39 | M | Catheter ablation of symptomatic, drug-refractory paroxysmal atrial fibrillation | S during the ablation procedure | NA | 70 ml/h | <2 h | Arrhythmia | No metabolic acidosis | Brugada pattern of coved-type > 2 mm ST-segment eleva­tion appeared in the right precordial leads V1 and V2 → after discontinuation of propofol coved-type ST-segment elevation gradual­ly resolved and no malignant ventric­ular arrhythmias occurred → normal sinus rhythm without any Bru­gada-like repolarization abnormalities | NA | Discontinuation of propofol infusion | To screen for drug-induced Brugada syndrome, a class I AAD challenge (aj­maline 1 mg/kg over 5 min) was per­formed but failed to unmask a char­acteristic coved-type Brugada electro­cardiogram. Genetic testing was negative for known cardiac sodi­um or calcium channel mutations re­lated to Brugada syndrome. | Survived |
| Annen et al.^85^  *2012* | 7 | M | Trauma brain injury | S | 7.3-12 | 12 | 49 hours | Fever, generalized muscle weakness and slow awakening, generalized seizure, progressive cardiac and pulmonary failure, peracute renal failure liver failure, rhabdomyolysis | Metabolic (hyperchloremic) acidosis (pH 7.19), ASAT ˃ 20,000U/L, creatine kinase ˃ 100,000 U/L, coagulopathy  (thrombin time ˃120 s, prothrombin time 48%) | Supraventricular tachycardia (up to 180 bpm) → bradycardia → asystole → resuscitation → bradyarrhytmia resistant to therapy | Concomitant use of norephinephrine, succinylcholine | Cessation of propofol infusion, mechanical and medical resuscitation, renal replacement therapy | CT brain scan: generalized edema of the brain with obliteration of the basal cisterns and transtentorial herniation; Muscle biopsy: histology revealed completely normal values, the muscle could not be stimulated for in vitro muscle contracture testing, but presented with spontaneous muscle contracture; Blood sample-molecular genetic investigations: no mutation in the gene encoding for the skeletal muscle type 1 ryanodine receptor (RyR1), altered intracellular calcium regulation via of dysfunction of ryanodine receptors | Died |
| Mijzen et al.^86^ 2012 | 23 | M | Trauma brain injury; a few subcortical and pontine high density lesions suspected for diffuse axonal injury (DAI) | S | 4.7-5.8 | 5.8 | 7 days | Arrhythmia, circulatory failure | Creatine kinase 246U/l, CKMB 36 U/l, HS-troponins 9ng/l, metabolic (hyperchloremic) acidosis (pH 7.28), hyperlactataemia 3.8mmol/l, hyperkalemia, hypertriglyceridemia 9,04 mmol/l, cardiac enzymes - normal | Biphasic T-waves in the leads II, III and aVF → broad QST-complexes, ST depression in leads II, III, aVF and V4-V6 coved ST-elevations in V1-V3 → refractory circulatory failure | Concomitent use of norepinephrine | Treatment with glucose and insulin and calcium gluconate, hemodialysis; | CT brain scan: diffuse brain swelling, a few subcortical and pontine high density lesions suspected for diffuse axonal injury (DAI); transthoracic echocardiography: normal left and right ventricular function; obduction: no signs of widespread infection, no structural abnormalities of the heart, and no cardial or hepatic histologic abnormalities | Died |
| Karaman et al.^87^  *2013* | 5 | F | Neonatal adrenoleukodystrophy – operation of bilateral tenotomy, myotomy, bilateral achilloplasty for the spasticity | A | Bolus 2.5 mg/kg + 50 mcg/kg/min | NA | 65 min | NA | Hypertriglyceridemia 697mg/dl, metabolic acidosis (pH 7.17), hyperpotassemia 5.4, mEq/dl, high levels of creatinine kinase 6640, CKMB 86.7, urea 12.8 mg/dl, SGOT (serum glutamic oxaloacetic transaminase) 199UI/l, and SGPT (serum glutamic pyruvic transaminase) 74 UI//l, normal urine organic acid analysis and normal tandem mass results | ECG normal | NA | NA | Echocardiography: normal; MRI: diffuse density loss of periventricular white matter; EEG: left hemisphere frontocentral paroxysmal aktivity; Detection of high levels of plasma very long-chain fatty acids (VLCFA) and mutation in the PEX1 gene | Survived |
| Imam et al.^88^  *2013* | 50 | M | Pneumothorax, aspiration pneumonia, respiratory distress | S | 5.1 | 5.1 | 6 days | Subfebrile temperature, hypotension, bradycardia, mild pulmonary edema, acute kidney injury | Severe lactic acidosis, leucocytosis 9550/uL, CK 51.000IU/L | Bradycardia → ventricular fibrillation | NA | Treatment by norepinephrine and dobutamine; intravenous fluids | A repeated chest  radiograph: pneumothorax and a  small zone of haziness on the right lower  side suggesting aspiration pneumonia | Died |
| Mayette et al.^89^  *2013* | 20 | F | Status epilepticus | S | NA | 9 mg/kg/h | 2 days | Fluid resistant shock, acute oliguric kidney failure, arrhythmia | Severe metabolic acidosis, serum  bicarbonate 10 mmol/L, lactate 11 mg/dL, a creatine kinase  655,200 U/L, white  blood cells count (WBC) 32 × 109/L, AST 3827 U/L,  ALT 789 U/L, alkaline phosphatase 193 U/L, total bilirubin  0.6 mg/dL, INR 1.4, hyperkalemia 8.8mmol/L, hypocalcemia | Right bundle branch block with left anterior  fascicular block →  unstable ventricular  tachycardia → sinus tachycardia with a persistent  right bundle branch block and septal lead  ST-segment depression →  intraventricular conduction  delays (IVCDs), progressively widening QRS, atrial flutter/  fibrillation, and an accelerated junctional escape rhythm →  ventricular tachycardia →  accelerated junctional rhythm/ventricular tachycardia →  ventricular fibrillation → ventricular tachycardia →  ventricular fibrillation → asystole → paced rhythm → a sinus rhythm  with minimal lateral T-wave abnormalities | NA | Crystalloids, epinephrine treatment, defibrillation, amiodarone, aggressive calcium/magnesium replacement, lidocaine, extracorporeal membrane oxygenation, vasopressin, bicarbonate,  calcium chloride, bicarbonate,  insulin, glucose, sodium polystyrene sulfonate, isoproterenol, dopamine, transvenous pacing, hemodialysis | NA | Survived |
| Agrawal et al.^90^  *2013* | 53 | F | Trauma head injury, polytrauma (multiple fractures with active bleeding in the pelvis, liver lacerations with active bleeding), angiogram embolization of right hepatic arterial branch  and right internal iliac artery for the hepatic and pelvic  bleeding | S | 1,2 – 5,7 | 5,7 | 5 days | Arrhythmia | Metabolic acidosis (pH  7.03, lactate 11.5 mmol/L) and hyperkalemia (K 9.3 mmol/L), hypertriglyceridemia | Recurrent episodes of ventricular  Tachycardia → a cardiac arrest with pulseless  electrical activity | Adrenaline 0.05–0.25 μg/kg/min (16 μg/250 ml dextrose 5 %) | Resuscitation, adrenaline  boluses and insulin infusion | CT scanning: subarachnoid  hemorrhages, intraparenchymal contusions, right  humeral neck fracture, grade 3 liver lacerations with active  bleeding, comminuted right superior and inferior pubic rami  fractures, right sacral ala fracture with active bleeding in the  pelvis; An exploratory laparotomy: evidence of hypoperfused small bowel,  large bowel, stomach and liver with no evidence of ischemia  or gangrene; liver laceration was also noted with no signs of  active bleeding. | Died |
| Schroeppel et al.^91^  *2014* | 27 | M | Trauma brain injury | S | NA | NA | NA | Rhabdomyolysis, cardiac arrhythmias | NA | Cardiac arrhythmias | NA | Stopping the propofol infusion | NA | Survived |
| Linko et al.^92^  *2014* | 19 | M | 45% TBSA flame burn (smoke inhalation injury suspicion) | A+S | 1.96-6.95 | 6.95 | 11 days | Hypotension, oliguria, rhabdomyolysis, heart failure, fever | Hyperlactataemia 6.1mmol/L, increased plasma creatinine concentration, serum troponin T (TnT) concentration  Increased, plasma myoglobin concentration increase to  1897 μg/L, no hypertermia, lactatemia,  electrolyte disorders, hypertriglyceridemia | ST-segment changes →  a Brugada-  type  ST-segment elevation developed with a further  increase in plasma TnT concentration → Brugada-like ECG pattern → normalization | Norepinephrine infusion at a dosage  of 0.01 μg/kg/min to 0.1 μg/kg/min  . | Norepinephrine, fluid resuscitation, sodium bicarbonate, renal replacement therapy and cardiac  support therapy, ontinuous veno-venous hemodiafiltration, levosimendan, hydrocortisone (suspected sepsis), | Transthoracic echocardiography: an extremely enlarged right side of  the heart with an increased pulmonary systolic pressure, left ventricular function normal → a normal  function of the heart; A muscle biopsy of the right  forearm: no signs of myocitis or  hereditary muscle pathologies, but signs of postnecrotic regeneration, typical for  rhabdomyolysis; CT scan of the thorax: significant stress of the right side of the  heart without pulmonary embolism. A focal muscle  compartment syndrome of gluteal muscle was ruled  out, with incision of a gluteal region revealing normal  muscle. A magnetic resonance image (MRI): a pathologic, diffuse muscle edema consistent with  rhabdomyolysis. X-ray scan of chest and mikrobiology: no pneumonia, sepsis | Survived |
| Poretti at al.^93^  *2014* | 3 | F | Sclerotherapy of large venous malformation | A | NA | NA | NA | Acute renal failure, rhabdomyolysis; transient neurological deficits (encephalopathy, weakness of arms and legs) | Metabolic acidosis | NA | NA | NA | MR: T2-hyperintensity and reduced diffusion  within the supra- and infratentorial white matter | Survived |
| Diaz et al.^94^  2014 | 32 | M | Trauma, postoperative development of ARDS | S | 50-125mcg/kg/min | 125mcg/kg/min | 8 days | Acute renal failure, rhabdomyolysis | Metabolic acidosis, hypertriglyceridemia 2,370 mg/dL, hyperkalaemia 7.1 mmol/l, creatine kinase 162,000U/L, elevated hepatic transaminases, no lipaemia | Cardiac arrest | NA | Norepinephrine, multiple vasopressors, hemodialysis | Autopsy:  acute bilateral pneumonia with pulmonary  congestion and edema, cardiomegaly (770 g) was  present with concentric left ventricular hypertrophy  (1.6 cm). The liver: evidence of chronic  passive congestion without hepatomegaly or evidence  of fatty liver | Died |

References:

1. Notis fra Bivirkningsnaenet. Propofol (Diprivan) bivirkninger. Ugeskr Laeger. 1990;152:1176.
2. Parke TJ, Stevens JE, Rice AS, Greenaway CL, Bray RJ, Smith PJ, Waldmann CS, Verghese C: Metabolic acidosis and fatal myocardial failure after propofol infusion in children: five case reports. BMJ 1992, 305:613–616.
3. Bodd E, Endresen L. Use of propofol for children. Tidsskr Nor Laegeforen. 1992;112:1636-1637.
4. Kirkpatrick M, Cole G. Propofol infusion in children. BMJ. 1992 Nov 14;305(6863):1223.
5. Barclay K, Williams AJ, Major E. Propofol infusion in children. BMJ. 1992;305(6859):953-954.
6. Bray RJ. Fatal myocardial failure associated with a propofol infusion in a child. Anaesthesia. 1995 Jan;50(1):94.
7. Strickland RA, Murray MJ.: Fatal metabolic acidosis in a pediatric patient receiving an infusion of propofol in the intensive care unit: is there a relationship? Crit Care Med. 1995 Feb;23(2):405-9.
8. Marinella MA. Lactic acidosis associated with propofol. Chest. 1996;109(1):292.
9. Plotz FB, Waalkens HJ, Verkade HJ, Strengers JL, Knoester H, Mandema JM. Fatal side-effects of continuous propofol infusion in children may be related to malignant hyperthermia. Anaesth Intensive Care. 1996;24(6):724.
10. van Straaten EA, Hendriks JJ, Ramsey G, Vos GD.  Rhabdomyolysis and pulmonary hypertension in a child, possibly due to long-term high-dose propofol infusion. Intensive Care Med. 1996 Sep;22(9):997.
11. Bray RJ: Propofol infusion syndrome in children. Pediatr Anesth 1998, 8:491–499.
12. Cray SH, Robinson BH, Cox PN. Lactic acidemia and bradyarrhythmia in a child sedated with propofol. Crit Care Med 1998;26:2087–2092.
13. Hanna JP, Ramundo ML. Rhabdomyolysis and hypoxia associated with prolonged propofol infusion in children. Neurology. 1998; 50(1):301-303.
14. Mehta N, DeMunter C, Habibi P, Nadel S, Britto J. Short-term propofol infusions in children. Lancet. 1999 Sep 4;354(9181):866-7.
15. Perrier ND, Baerga-Varela Y, Murray MJ. Death related to propofol use in an adult patient. Crit Care Med. 2000 Aug;28(8):3071-4.
16. Stelow EB, Johari VP, Smith SA, Crosson JT, Apple FS. Propofol-associated  rhabdomyolysis with cardiac involvement in adults: chemical and anatomic findings. Clin Chem. 2000;46(4):577-581.
17. Badr AE, Mychaskiw G 2nd, Eichhorn JH. Metabolic acidosis associated with a new formulation of propofol.  Anesthesiology. 2001 Mar;94(3):536-8.
18. Cannon ML, Glazier SS, Bauman LA. Metabolic acidosis, rhabdomyolysis, and cardiovascular collapse after prolonged propofol infusion. J Neurosurg. 2001 Dec;95(6):1053-6.
19. Cremer OL, Moons KG, Bouman EA, Kruijswijk JE, de Smet AM, Kalkman CJ. Long-term propofol infusion and cardiac failure in adult head-injured patients. Lancet. 2001 Jan 13;357(9250):117-8.
20. Wolf A, Weir P, Segar P, Stone J, Shield J. Impaired fatty acid oxidation in propofol infusion syndrome. Lancet. 2001 Feb 24;357(9256):606-7.
21. Kelly DF. Propofol-infusion syndrome. J Neurosurg. 2001 Dec;95(6):925-6.
22. Friedman JA, Manno E, Fulgham JR. Propofol. J Neurosurg. 2002 Jun;96(6):1161-1162.
23. Abrahams JM, Reiter GT, Acker MA, Sinson GP. J Neurosurg. 2002 Jun;96(6):1160-1161.
24. Ernest D, French C. Propofol infusion syndrome--report of an adult fatality. Anaesth Intensive Care. 2003 Jun;31(3):316-9.
25. Kill C, Leonhardt A, Wulf H. Lacticacidosis after short-term infusion of propofol for anaesthesia in a child with osteogenesis imperfecta. Paediatr Anaesth. 2003 Nov;13(9):823-6.
26. Lewejohann J C, Hansen M, Zimmermann C, Muhl E, Bruch H P. Propofol infusion syndrome in a patient with severe multiple trauma. European Society of Intensive Care Medicine, 17th Annual Congress. Berlin,Germany, 10-13 October 2004.
27. Koch M, De Backer D, Vincent JL. Lactic acidosis: an early marker of propofol infusion syndrome? Intensive Care Med. 2004 Mar;30(3):522.
28. Holzki J, Aring C, Gillor A. Death after re-exposure to propofol in a 3-year-old child: a case report. Paediatr Anaesth. 2004 Mar;14(3):265-70.
29. Withington DE, Decell MK, Al Ayed T. A case of propofol toxicity: further evidence for a causal mechanism. Paediatr Anaesth. 2004;14(6):505-508.
30. Culp KE, Augoustides JG, Ochroch AE, Milas BL. [Clinical management of cardiogenic shock associated with prolonged propofol infusion.](http://www.ncbi.nlm.nih.gov/pubmed/15281533) Anesth Analg. 2004 Jul;99(1):221-6.
31. Baumeister FA, Oberhoffer R, Liebhaber GM, Kunkel J, Eberhardt J, Holthausen H, Peters J. Fatal propofol infusion syndrome in association with ketogenic diet. Neuropediatrics. 2004;35(4):250-252.
32. Casserly B, O’Mahony E, Timm EG, Haqqie S, Eisele G, Urizar R. Propofol infusion syndrome: an unusual cause of renal failure. Am J Kidney Dis. 2004;44(6):e98-e101.
33. Burow BK, Johnson ME, Packer DL. Metabolic acidosis associated with propofol in the absence of other causative factors. Anesthesiology. 2004 Jul;101(1):239-41.
34. Salengros JC, Velghe-Lenelle CE, Bollens R, Engelman E, Barvais L. Lactic acidosis during propofol-remifentanil anesthesia in an adult. Anesthesiology. 2004;101:241–3.
35. Haase R, Sauer H, Eichler G. Lactic acidosis following short-term propofol infusion may be an early warning of propofol infusion syndrome. J Neurosurg Anesthesiol. 2005 Apr;17(2):122-3.
36. Liolios A, Guérit JM,  Scholtes JL, Raftopoulos C, Hantson P.  Propofol infusion syndrome associated with short-term large-dose infusion during surgical anesthesia in an adult. Anesth Analg. 2005 Jun;100(6):1804-6.
37. Kumar MA, Urrutia VC, Thomas CE, Abou-Khaled KJ, Schwartzman RJ. The syndrome of irreversible acidosis after prolonged propofol infusion. Neurocrit Care. 2005;3(3):257-259.
38. Machata AM, Gonano C, Birsan T, Zimpfer M, Spiss CK. Rare but dangerous adverse effects of propofol and thiopental in intensive care. J Trauma. 2005;58(3):643-645.
39. Suen HC, Haake RJ, Chavez VM, Hayat SA. A lethal complication of propofol. Asian Cardiovasc Thorac Ann. 2006 Feb;14(1):60-2.
40. Eriksen J, Povey HM. A case of suspected non-neurosurgical adult fatal propofol infusion syndrome. Acta Anaesthesiol Scand. 2006;50(1):117-119.
41. Vernooy K, Delhaas T, Cremer OL, Di Diego JM, Oliva A, Timmermans C, Volders PG, Prinzen FW, Crijns HJGM, Antzelevitch C, Kalkman CJ, Rodriguez L-M, Brugada R: Electrocardiographic changes predicting sudden death in propofol-related infusion syndrome. Heart Rhythm 2006, 3:131–137.
42. Merz TM, Regli B, Rothen HU, Felleiter P. Propofol infusion syndrome–a fatal case at a low infusion rate. Anesth Analg. 2006; 103(4):1050.
43. Corbett SM, Moore J, Rebuck JA, Rogers FB, Greene CM. Survival of propofol infusion syndrome in a head-injured patient. Crit Care Med. 2006 Sep;34(9):2479-83.
44. Hermanns H, Lipfert P, Ladda S, Stevens MF. Propofol infusion syndrome during anaesthesia for scoliosis surgery in an adolescent with neonatal progeroid syndrome. Acta Anaesthesiol Scand. 2006 Mar;50(3):392-4.
45. Trampitsch E, Oher M, Pointner I, Likar R, Jost R, Schalk HV. [Propofol infusion syndrome]. Anaesthesist. 2006 Nov;55(11):1166-8.
46. Chukwuemeka A, Ko R, Ralph-Edwards A. Short-term low-dose propofol anaesthesia associated with severe metabolic acidosis. Anaesth Intensive Care. 2006 Oct;34(5):651-5.
47. De Waele JJ, Hoste E. Propofol infusion syndrome in a patient with sepsis. Anaesth Intensive Care. 2006 Oct;34(5):676-7.
48. Sabsovich I, Rehman Z, Yunen J, Coritsidis G. Propofol infusion syndrome: a case of increasing morbidity with traumatic brain injury. Am J Crit Care. 2007;16(1):82-85.
49. Westhout FD, Muhonen MG, Nwagwu CI. Early propofol infusion syndrome following cerebral angiographic embolization for giant aneurysm repair. Case report. J Neurosurg. 2007 Feb;106(2 Suppl):139-42.
50. Zarovnaya EL, Jobst BC, Harris BT. Propofol-associated fatal myocardial failure and rhabdomyolysis in an adult with status epilepticus. Epilepsia. 2007;48(5):1002-1006.
51. Karakitsos D, Poularas J, Kalogeromitros A, Karabinis A. The propofol infusion syndrome treated with haemofiltration. Is there a fime for genetic screening? Acta Anaesthesiol Scand. 2007 May;51(5):644-5.
52. Rosen DJ, Nicoara A, Koshy N, Wedderburn RV. Too much of a good thing? Tracing the history of the propofol infusion syndrome.  J Trauma. 2007 Aug;63(2):443-7.
53. Bordes J, Meaudre E, Asencio Y, Montcriol A, Kaiser E. [Lactic acidosis associated with propofol during general anaesthesia for neurosurgery]. Ann Fr Anesth Reanim. 2008 Mar;27(3):261-4.
54. Fudickar A, Tonner PH, Mihaljovic Z, Dellien C, Weiler N, Scholz J, Bein B. Suggested beginning of propofol infusion syndrome in an adult patient without lactacidosis: a case report. Eur J Anaesthesiol. 2008 Sep; 25(9):777-8.
55. Romero P C, Morales R M, Donaire R L, Llanos V O, Cornejo R R, G8lvey A R, Castro O J.  [Severe lactic acidosis caused by propofol infusion: report of one case]. Rev Med Chil. 2008 Jan;136(1):88-92.
56. Shimony A, Almog Y, Zagher D. Propofol infusion syndrome: a rare cause of multi-organ failure in a man with complicated myocardial infarction. Isr Med Assoc J. 2008 Apr;10(4):316-7.
57. Robinson JD, Melman Y, Walsh EP. Cardiac conduction disturbances and ventricular tachycardia after prolonged propofol infusion in an infant. Pacing Clin Electrophysiol. 2008 Aug;31(8):1070-3.
58. Zaccheo MM, Bucher DH.  Propofol infusion syndrome: a rare complication with potentially fatal results. Crit Care Nurse. 2008 Jun;28(3):18-26.
59. Aloizos S, Gourgiotis S, Oikonomou K, Stakia P. Acute renal failure and rhabdomyolysis in a patient with infectious mononucleosis: a case report. Cases J. 2008 Oct 7;1(1):222.
60. Laquay N, Pouard P, Silicani MA, Vaccaroni L, Orliaguet G. Early stages of propofol infusion syndrome in paediatric cardiac surgery: two cases in adolescent girls. Br J Anaesth. 2008 Dec;101(6):880-1.
61. Smith H, Sinson G, Varelas P. Vasopressors and propofol infusion syndrome in severe head trauma. Neurocrit Care. 2009;10(2):166-72.
62. [Ilyas MI](http://www.ncbi.nlm.nih.gov/pubmed/?term=Ilyas%20MI%5BAuthor%5D&cauthor=true&cauthor_uid=19101256), [Balacumaraswami L](http://www.ncbi.nlm.nih.gov/pubmed/?term=Balacumaraswami%20L%5BAuthor%5D&cauthor=true&cauthor_uid=19101256), [Palin C](http://www.ncbi.nlm.nih.gov/pubmed/?term=Palin%20C%5BAuthor%5D&cauthor=true&cauthor_uid=19101256), [Ratnatunga C](http://www.ncbi.nlm.nih.gov/pubmed/?term=Ratnatunga%20C%5BAuthor%5D&cauthor=true&cauthor_uid=19101256). Propofol infusion syndrome in adult cardiac surgery. [Ann Thorac Surg.](http://www.ncbi.nlm.nih.gov/pubmed/?term=ilyas+propofol+infusion) 2009 Jan;87(1):e1-3.
63. [Veldhoen ES](http://www.ncbi.nlm.nih.gov/pubmed/?term=Veldhoen%20ES%5BAuthor%5D&cauthor=true&cauthor_uid=19265362), [Hartman BJ](http://www.ncbi.nlm.nih.gov/pubmed/?term=Hartman%20BJ%5BAuthor%5D&cauthor=true&cauthor_uid=19265362), [van Gestel JP](http://www.ncbi.nlm.nih.gov/pubmed/?term=van%20Gestel%20JP%5BAuthor%5D&cauthor=true&cauthor_uid=19265362). Monitoring biochemical parameters as an early sign of propofol infusion syndrome: false feeling of security. [Pediatr Crit Care Med.](http://www.ncbi.nlm.nih.gov/pubmed/?term=veldhoen+propofol) 2009 Mar;10(2):e19-21.
64. Orsini J, Nadkarni A, Chen J, Cohen N. Propofol infusion syndrome: case report and literature review. Am J Health Syst Pharm. 2009 May 15;66(10):908-15.
65. Iyer VN, Hoel R, Rabinstein AA. Propofol infusion syndrome in patients with refractory status epilepticus: an 11-year clinical experience. Crit Care Med. 2009 Dec;37(12):3024-30.
66. Mali AR, Patil VP, Pramesh CS, Mistry RC. Hyperkalemia during surgery: is it an early warning of propofol infusion syndrome? J Anesth. 2009;23(3):421-3.
67. Blum JM, Brunsvold ME. Non-acidotic propofol infusion syndrome. Br J Anaesth. 2009 Oct;103(4):617-8.
68. Jorens PG, Van den Eyden GG. Propofol infusion syndrome with arrhythmia, myocardial fat accumulation and cardiac failure. Am J Cardiol. 2009 Oct 15;104(8):1160-2.
69. Weiner JB, Haddad EV, Raj SR. Recovery following propofol-associated brugada electrocardiogram. Pacing Clin Electrophysiol. 2010 Apr;33(4):e39-42.
70. Roberts RJ, Barletta JF, Fong JJ, Schumaker G, Kuper PJ, Papadopoulos S, Yogaratnam D, Kendall E, Xamplas R, Gerlach AT, Szumita PM, Anger KE, Arpino PA, Voils SA, Grgurich P, Ruthazer R, Devlin JW. Incidence of propofol-related infusion syndrome in critically ill adults: a prospective, multicenter study. Crit Care. 2009; 13(5):R169.
71. Da-Silva SS, Wong R, Coquillon P, Gavrilita C, Asuncion A. Partial-exchange blood transfusion: an effective method for preventing mortality in a child with propofol infusion syndrome. Pediatrics. 2010 Jun;125(6):e1493-9.
72. Guitton C, Gabillet L, Latour P, Rigal JC, Boutoille D, Al Habash O, Derkinderen P, Bretonniere C, Villers D.  Propofol Infusion syndrome during refractory status epilepticus in a young adult: successful ECMO resuscitation. Neurocrit Care. 2011 Aug;15(1):139-45.
73. Sammartino M, Garra R, Sbaraglia F, Papacci P. Propofol overdose in a preterm baby: may propofol infusion syndrome arise in two hours? Paediatr Anaesth. 2010 Oct;20(10):973-4.
74. Soler-Rodenas A, Manero E, Marruecos L, Mangues MA. [Rhabdomyolysis and metabolic acidosis associated with propofol use]. Farm Hosp. 2010 Mar-Apr;34(2):99-100.
75. [Power KN](http://www.ncbi.nlm.nih.gov/pubmed/?term=Power%20KN%5BAuthor%5D&cauthor=true&cauthor_uid=21300522), [Flaatten H](http://www.ncbi.nlm.nih.gov/pubmed/?term=Flaatten%20H%5BAuthor%5D&cauthor=true&cauthor_uid=21300522), [Gilhus NE](http://www.ncbi.nlm.nih.gov/pubmed/?term=Gilhus%20NE%5BAuthor%5D&cauthor=true&cauthor_uid=21300522), [Engelsen BA](http://www.ncbi.nlm.nih.gov/pubmed/?term=Engelsen%20BA%5BAuthor%5D&cauthor=true&cauthor_uid=21300522). Propofol treatment in adult refractory status epilepticus. Mortality risk and outcome. [Epilepsy Res.](http://www.ncbi.nlm.nih.gov/pubmed/21300522) 2011 Mar;94(1-2):53-60.
76. Amrein S, Amrein K, Amegah-Sakotnik A, Reist U, Ensner R. Propofol infusion syndrome--a critical incident report highlighting the danger of reexposure. J Neurosurg Anesthesiol. 2011 Jul;23(3):265-6.
77. Diedrich DA, Brown DR. Analytic reviews: propofol infusion syndrome in the ICU. J Intensive Care Med. 2011 Mar-Apr;26(2):59-72.
78. Faulkner MJ, Haley MW, Littmann L. Propofol infusion syndrome with severe and dynamic Brugada electrocardiogram but benign clinical outcome. J Cardiovasc Electrophysiol. 2011 Jul;22(7):827-8.
79. Ramaiah R, Lollo L, Brannan D, Bhananker SM. Propofol infusion syndrome in a super morbidly obese patient (BMI = 75). Int J Crit Illn Inj Sci. 2011 Jan;1(1):84-6.
80. Testerman GM, Chow TT, Easparam S 4th. Propofol infusion syndrome: an algorithm for prevention. Am Surg. 2011 Dec;77(12):1714-5.
81. Pisapia JM, Wendell LC, Kumar MA, Zager EL, Levine JM. Lactate-to-pyruvate ratio as a marker of propofol infusion syndrome after subarachnoid hemorrhage. Neurocrit Care. 2011 Aug;15(1):134-8.
82. Vanlander AV, Jorens PG, Smet J, De Paepe B, Verbrugghe W, Van den Eynden GG, Meire F, Pauwels P, Van der Aa N, Seneca S, Lissens W, Okun JG, Van Coster R. Inborn oxidative phosphorylation defect as risk factor for propofol infusion syndrome. Acta Anaesthesiol Scand. 2012 Apr;56(4):520–5.
83. [Annecke T](http://www.ncbi.nlm.nih.gov/pubmed/?term=Annecke%20T%5BAuthor%5D&cauthor=true&cauthor_uid=22284319), [Conzen P](http://www.ncbi.nlm.nih.gov/pubmed/?term=Conzen%20P%5BAuthor%5D&cauthor=true&cauthor_uid=22284319), [Ney L](http://www.ncbi.nlm.nih.gov/pubmed/?term=Ney%20L%5BAuthor%5D&cauthor=true&cauthor_uid=22284319). Propofol-related infusion syndrome induced by "moderate dosage" in a patient with severe head trauma. [J Clin Anesth.](http://www.ncbi.nlm.nih.gov/pubmed/?term=Propofol+infusion+syndrome%3A+annecke) 2012 Feb;24(1):51-4.
84. [Richter S](http://www.ncbi.nlm.nih.gov/pubmed/?term=Richter%20S%5BAuthor%5D&cauthor=true&cauthor_uid=22302080), [Brugada P](http://www.ncbi.nlm.nih.gov/pubmed/?term=Brugada%20P%5BAuthor%5D&cauthor=true&cauthor_uid=22302080). Propofol-induced coved-type electrocardiogram during catheter ablation of paroxysmal atrial fibrillation. A case of Brugada syndrome? [Herzschrittmacherther Elektrophysiol.](http://www.ncbi.nlm.nih.gov/pubmed/?term=Propofol+infusion+syndrome%3A+richter) 2012 Mar;23(1):56-8.
85. [Annen E](http://www.ncbi.nlm.nih.gov/pubmed/?term=Annen%20E%5BAuthor%5D&cauthor=true&cauthor_uid=24765478), [Girard T](http://www.ncbi.nlm.nih.gov/pubmed/?term=Girard%20T%5BAuthor%5D&cauthor=true&cauthor_uid=24765478), [Urwyler A](http://www.ncbi.nlm.nih.gov/pubmed/?term=Urwyler%20A%5BAuthor%5D&cauthor=true&cauthor_uid=24765478). Rare, potentially fatal, poorly understood propofol infusion syndrome. [Clin Pract.](http://www.ncbi.nlm.nih.gov/pubmed/?term=Propofol+infusion+syndrome%3A+annen) 2012 Sep 3;2(3):e79.
86. [Mijzen EJ](http://www.ncbi.nlm.nih.gov/pubmed/?term=Mijzen%20EJ%5BAuthor%5D&cauthor=true&cauthor_uid=22847396), [Jacobs B](http://www.ncbi.nlm.nih.gov/pubmed/?term=Jacobs%20B%5BAuthor%5D&cauthor=true&cauthor_uid=22847396), [Aslan A](http://www.ncbi.nlm.nih.gov/pubmed/?term=Aslan%20A%5BAuthor%5D&cauthor=true&cauthor_uid=22847396), [Rodgers MG](http://www.ncbi.nlm.nih.gov/pubmed/?term=Rodgers%20MG%5BAuthor%5D&cauthor=true&cauthor_uid=22847396). Propofol infusion syndrome heralded by ECG changes. [Neurocrit Care.](http://www.ncbi.nlm.nih.gov/pubmed/?term=Propofol+infusion+syndrome%3A+mijzen) 2012 Oct;17(2):260-4.
87. [Karaman Y](http://www.ncbi.nlm.nih.gov/pubmed/?term=Karaman%20Y%5BAuthor%5D&cauthor=true&cauthor_uid=23302011), [Goktay A](http://www.ncbi.nlm.nih.gov/pubmed/?term=Goktay%20A%5BAuthor%5D&cauthor=true&cauthor_uid=23302011), [Agin H](http://www.ncbi.nlm.nih.gov/pubmed/?term=Agin%20H%5BAuthor%5D&cauthor=true&cauthor_uid=23302011), [Karaarslan U](http://www.ncbi.nlm.nih.gov/pubmed/?term=Karaarslan%20U%5BAuthor%5D&cauthor=true&cauthor_uid=23302011). Propofol infusion syndrome or adrenoleukodystrophy? [Paediatr Anaesth.](http://www.ncbi.nlm.nih.gov/pubmed/?term=Propofol+infusion+syndrome%3A+karaman) 2013 Apr;23(4):368-70.
88. Imam TH. Propofol-related infusion syndrome: role of propofol in medical complications of sedated critical care patients. Perm J. 2013 Spring;17(2):85-7.
89. [Mayette M](http://www.ncbi.nlm.nih.gov/pubmed/?term=Mayette%20M%5BAuthor%5D&cauthor=true&cauthor_uid=24059786), [Gonda J](http://www.ncbi.nlm.nih.gov/pubmed/?term=Gonda%20J%5BAuthor%5D&cauthor=true&cauthor_uid=24059786), [Hsu JL](http://www.ncbi.nlm.nih.gov/pubmed/?term=Hsu%20JL%5BAuthor%5D&cauthor=true&cauthor_uid=24059786), [Mihm FG](http://www.ncbi.nlm.nih.gov/pubmed/?term=Mihm%20FG%5BAuthor%5D&cauthor=true&cauthor_uid=24059786). Propofol infusion syndrome resuscitation with extracorporeal life support: a case report and review of the literature. [Ann Intensive Care.](http://www.ncbi.nlm.nih.gov/pubmed/?term=Propofol+infusion+syndrome%3A+mayette) 2013 Sep 23;3(1):32.
90. [Agrawal N](http://www.ncbi.nlm.nih.gov/pubmed/?term=Agrawal%20N%5BAuthor%5D&cauthor=true&cauthor_uid=24426631), [Rao S](http://www.ncbi.nlm.nih.gov/pubmed/?term=Rao%20S%5BAuthor%5D&cauthor=true&cauthor_uid=24426631), [Nair R](http://www.ncbi.nlm.nih.gov/pubmed/?term=Nair%20R%5BAuthor%5D&cauthor=true&cauthor_uid=24426631). A death associated with possible propofol infusion syndrome. [Indian J Surg.](http://www.ncbi.nlm.nih.gov/pubmed/?term=Propofol+infusion+syndrome%3A+agrawal) 2013 Jun;75(Suppl 1):407-8.
91. [Schroeppel TJ](http://www.ncbi.nlm.nih.gov/pubmed/?term=Schroeppel%20TJ%5BAuthor%5D&cauthor=true&cauthor_uid=23742861), [Fabian TC](http://www.ncbi.nlm.nih.gov/pubmed/?term=Fabian%20TC%5BAuthor%5D&cauthor=true&cauthor_uid=23742861), [Clement LP](http://www.ncbi.nlm.nih.gov/pubmed/?term=Clement%20LP%5BAuthor%5D&cauthor=true&cauthor_uid=23742861), [Fischer PE](http://www.ncbi.nlm.nih.gov/pubmed/?term=Fischer%20PE%5BAuthor%5D&cauthor=true&cauthor_uid=23742861), [Magnotti LJ](http://www.ncbi.nlm.nih.gov/pubmed/?term=Magnotti%20LJ%5BAuthor%5D&cauthor=true&cauthor_uid=23742861), [Sharpe JP](http://www.ncbi.nlm.nih.gov/pubmed/?term=Sharpe%20JP%5BAuthor%5D&cauthor=true&cauthor_uid=23742861), [Lee M](http://www.ncbi.nlm.nih.gov/pubmed/?term=Lee%20M%5BAuthor%5D&cauthor=true&cauthor_uid=23742861), [Croce MA](http://www.ncbi.nlm.nih.gov/pubmed/?term=Croce%20MA%5BAuthor%5D&cauthor=true&cauthor_uid=23742861). Propofol infusion syndrome: a lethal condition in critically injured patients eliminated by a simple screening protocol. [Injury.](http://www.ncbi.nlm.nih.gov/pubmed/?term=Propofol+infusion+syndrome%3A+schroeppel) 2014 Jan;45(1):245-9.
92. Linko R, Laukkanen A, Koljonen V, Rapola J, Varpula T. Severe heart failure and rhabdomyolysis associated with propofol infusion in a burn patient. J Burn Care Res. 2014 Sep-Oct;35(5):e364-7.
93. Poretti A, Bosemani T, Huisman TA. Neuroimaging findings in pediatric propofol infusion syndrome. Pediatr Neurol. 2014 Apr;50(4):431-2.
94. Diaz JH, Roberts CA, Oliver JJ, Kaye AD. Propofol infusion syndrome or not? A case report. Ochsner J. 2014 Fall;14(3):434-7.

# Part S2: Step-by-step multiple logistic regression

Last change: 7.5.2015

Statistician: P. Waldauf

Software: Stata 14

Content of Part S2

Part S2: Step-by-step multiple logistic regression 56

Descriptive statistics + univariant mortality analysis 59

Mortality 59

Age 59

Child 65

Sex 66

Underlying dg 67

Resp.infection 67

TBI 68

Status epilepticus 69

Non-trauma neurological dg. 70

Other 71

Average infusion rate 72

Duration 78

Cumulative dose mg/kg 83

Symtoms 89

Arrhythmia 90

Cardiac failure 90

Metabolic acidosis 91

Lipaemia or elevated triacylglyceroles (TAG) 92

Hyperkalemia 93

Derranged liver function tests 94

Rhabdomyolysis (myo, ^CK) 95

AKI (anuria, oliguria, ^creatinine) 96

Discoloration of urine 97

Hepatomegaly or fatty infiltration of liver 98

Pulmonary oedema 99

Fever 100

Hypotension 101

ECG changes 102

Multivariant analysis 103

Missing values analysis 103

Correlation analysis 104

Symptoms 104

Model 1 107

Additional calculations 109

Fever and cummulative dose 109

Fever and average infusion rate 111

# Descriptive statistics + univariant mortality analysis

## Mortality

tab Mortality

Mortalita in the whole data set is 51,32%

## Age

tabstat Age , stat( N mean sd median q min max)

graph box Age

histogram Age

#### Relation to mortality:

tabstat Age , by( Mortality ) stat( N mean sd median q min max)

graph box Age, by(Mortality)

regress Age i.Mortality

Pacienti who died are on average by 7.7 years younger (p=0,021). Now we divide patients into subgroups according to age and compare mortality among these groups. .

##### Patients divided into 5 pentiles of age:

xtile Age_pentiles= Age , nq(5)

tabstat Age , by( Age_pentiles ) stat( N mean sd median q min max)

tab Age_pentiles Mortality, row

Table shows that the lowest mortality (13.8%) is in the 5th age pentile (55-71 yr). In other age groups (0.2-53 yr) is mortality very uniform around 60%.

logistic Mortality i.Age_pentiles

pwcompare Age_pentiles , effects

logistic Mortality i.Age_pentiles

margins Age_pentiles

marginsplot, xdimension( Age_pentiles ) xscale(range(0.5 3.5)) title("MORTALITY") legend(off) ytitle("") xtitle(Age pentiles[years])

I create a Binary parametr AgeBelow55 (coding: 55+ years = 0, <55 years = 1)

recode Age_pentiles (5 = 0) (1 = 1) (2=1) (3=1) (4=1), gen(AgeBelow55)

tab Age_pentiles AgeBelow55

logistic Mortality i.AgeBelow55

Pacients <55 years have higher mortality OR 8.9 p<0,001,logistic regression.

## Child

tab Child

The group contains 36,4% children <18 years

#### Relation to mortality:

tab Child Mortality, row

Mortality of children is 56% and of adults 48% which is not significantly different (logistic regression p=0,318).

logistic Mortality i.Child

Lastly I explore whether small children (<2 yrs) differ in mortality.

gen AgeBelow2 =.

replace AgeBelow2 = 1 if Age < 2

tab AgeBelow2 Mortality, row

There are 14 babies in the dataset and their mortality is 50%.

## Sex

tab Male

logistic Male

There is 60,2% of female patients in the dataset. Of note, sex is not known for 19 patients.

#### Relation to mortality:

tab Male Mortality, row

logistic Mortality i.Male

There is no difference in mortality between female (49%) and male (45%) patients – (logistic regression p=0,646).

## Underlying dg

### Resp.infection

tab Resp_infection

14,5% pacients had respirátory infection.

#### Relation to mortality:

tab Resp_infection Mortality, row

logistic Mortality i.Resp_infection

Mortality of patients with Resp Inf is 68.2%, as compared to 48.5% in patients without respirátory infection , OR 2.3 p=0.093

### TBI

tab TBI

There are 28.3% of patients with TBI.

#### Relations to mortality:

tab TBI Mortality, row

logistic Mortality i.TBI

Pacients with TBI have mortality of 76.7% and without TBI 41.3%, OR 4,7 p< 0,001 (logistic regression).

### Status epilepticus

**tab Status_epilepticus** **tab Status_epilepticus**

tab Status_epilepticus

There are 19.7% of patients with status epilepticus .

#### Relations to mortality:

tab Status_epilepticus Mortality, row

logistic Mortality i.Status_epilepticus

Pacients with SE have mortality of 43.3% as compared to 53.3% in patients without SE (p=0.331, logistic regression).

### Non-trauma neurological dg.

tab Nontrauma_neuro_dg

There are 9.9% of patients with non-traumatic non-epileptic neurological dg.

#### Relations to mortality:

tab Nontrauma_neuro_dg Mortality, row

logistic Mortality i.Nontrauma_neuro_dg

Patients with non-traumatic neurological dg. Have mortality of 40% as compared to 52.6% , p=0.36 (logistic regression)

### Other

replace Other = 0 if Other ==.

tab Other

There are 28.1% of patients with other diagnoses.

#### Relation to mortality:

tab Other Mortality, row

logistic Mortality i.Other

Pacients with Other diagnoses have mortality of 27.9% as compared to 60.6% in the restof the data set, p<0,001 OR 0,25, logistic regression.

## Average infusion rate

tabstat Average_dose , stat( N mean sd median q min max)

Missing data in 153-128 = 25 pacients

graph box Average_dose

#### Relation to mortality:

tabstat Average_dose, by( Mortality ) stat( N mean sd median q min max)

graph box Average_dose, by(Mortality)

regress Average_dose i.Mortality

Nonsurvivors recieved on average 6.7 mg/kg/h as compared to 5.8 mg/kg/h in survivors, p=0,076, linear regression.

logistic Mortality c.Average_dose

Now I divide patients into pentils and

xtile Average_dose_pentiles= Average_dose, nq(5)

tabstat Average_dose, by(Average_dose_pentiles ) stat( N mean sd median q min max)

tab Average_dose_pentiles Mortality, row

logistic Mortality i. Average_dose_pentiles

margins Average_dose_pentiles

marginsplot, xdimension( Average_dose_pentiles ) xscale(range(0.5 3.5)) title("MORTALITY") legend(off) ytitle("") xtitle(Average dose pentiles[mg/kg/hr])

pwcompare Average_dose_pentiles , effects

logistic Mortality c.Average_dose

margins, at (Average_dose =( 1(1) 15))

marginsplot, recastci(rarea) ci1opts(fintensity(20) lwidth(vvthin))

Now I create categorial parameter Average_dose_above5, identifiying patients with PRIS induced by the dose abowe the maximum allowed propofol dose. I will merge pentils 1 and 2 (Group 0) and pentiles 3, 4 and 5 (Group 1)

recode Average_dose_pentiles (1 = 0) (2=0) (3=1) (4=1) (5 = 1), gen(Average_dose_above5)

logistic Mortality i.Average_dose_above5

Pacients in Group 1 have higher mortality than Group 0 – OR 4,2, p<0,001

## Duration

tabstat Duration , stat( N mean sd median q min max)

graph box Duration

#### Relations to mortaliny:

tabstat Duration , by( Mortality ) stat( N mean sd median q min max)

graph box Duration , by(Mortality)

regress Duration i.Mortality

There is a trend to longer duration in nonsurvivors (mean 80 hours) as compared to survivors (mean 67 hours), p=0,107 linear regression.

Now I divide patients into pentils according to infusion duration and compare mortality among them.

xtile Duration_pentiles= Duration, nq(5)

tabstat Duration, by(Duration_pentiles ) stat( N mean sd median q min max)

tab Duration_pentiles Mortality, row

logistic Mortality i. Duration_pentiles

margins Duration_pentiles

marginsplot, xdimension( Duration_pentiles ) xscale(range(0.5 3.5)) title("MORTALITY") legend(off) ytitle("") xtitle(Duration pentiles[hrs])

pwcompare Duration_pentiles , effects

tyto kategorie se dají spojit: 3+4+5

There is similar mortality in 3rd, 4th and 5th pentils and they can be merged into 1 category (Duration above 60 hours)

recode Duration_pentiles (1 = 1) (2=2) (3=3) (4=3) (5 = 3), gen(Duration_3)

tabstat Duration, by( Duration_3 ) stat( N mean sd median q min max)

Now we have 3 categories of duration of propofol infusion: <20 hours, 20 -60hours and above 60 hours

logistic Mortality i.Duration_3

logistic Mortality i.Duration_3

pwcompare Duration_3 , or effects

As compared to patients with infusion duration <20 min, pacienti with duration of infusion 20-60 min have higher mortality O(R 43.5, p<0,001) as well as patients with the duration above 60 hours (OR 10.6, p<0,001)

## Cumulative dose mg/kg

tabstat Cum_dose , stat( N mean sd median q min max)

Missing data 152-128 = 24 pacients.

graph box Cum_dose

#### vztah k mortalitě:

tabstat Cum_dose, by( Mortality ) stat( N mean sd median q min max)

graph box Cum_dose , by(Mortality)

regress Cum_dose i.Mortality

Nonsurvivors were exposed to cummulative dose by 111 mg/kg higher than survivors . p=0,074. Now mortality will be compared in pentiles according to cummulative dose.

xtile Cum_dose_pentiles= Cum_dose, nq(5)

tabstat Cum_dose , by( Cum_dose_pentiles ) stat( N mean sd median q min max)

tab Cum_dose_pentiles Mortality, row

logistic Mortality i.Cum_dose_pentiles

pwcompare Cum_dose_pentiles , effects

logistic Mortality i.Cum_dose_pentiles

marginsplot, xdimension( Cum_dose_pentiles ) xscale(range(0.5 3.5)) title("MORTALITY") legend(off) ytitle("") xtitle(Cum dose[mg/kg])

logistic Mortality Cum_dose

margins, at (Cum_dose=( 0(200) 2000))

marginsplot, recastci(rarea) ci1opts(fintensity(20) lwidth(vvthin)) ci2opts(fintensity(20) lwidth(vvthin))

marginsplot, recastci(rarea) ci1opts(fintensity(20) lwidth(vvthin))

Pentiles 1-2 and 3-4-5 can be merged into 2 categories (Cummulative dose above or below 360 mg/kg)

recode Cum_dose_pentiles (1 = 0) (2=0) (3=1) (4=1) (5=1), gen(Cum_dose_above360)

tabstat Cum_dose , by( Cum_dose_above360 ) stat( N mean sd median q min max)

logistic Mortality i.Cum_dose_above360

Pacients exposed to cummulative dose above 360 mg/kg have higher mortality: OR 2.74, p=0.007

##

## Symtoms

### Arrhythmia

tab Arrhythmia

66.5% pacients had arrhytmia

#### Relation to mortality:

tab Arrhythmia Mortality, row

logistic Mortality i.Arrhythmia

Mortalitu with arrhytmia is 63.4%, without arrhytmia 27.5%, OR 4.6, p<0,001, logistic regression

### Cardiac failure

tab Cardiac_failure

23% had cardiac failure.

#### Relation to mortality:

tab Cardiac_failure Mortality, row

logistic Mortality i.Cardiac_failure

There is no difference in mortality in patients with and without heart failure (50.4 vs 54.3 %, p=0.69).

### Metabolic acidosis

tab MAC

77% had metabolic acidosis

#### Relation to mortality:

tab MAC Mortality, row

logistic Mortality i.MAC

Mortality with MAC is 56.4% and without MAC is 34.3%, p=0,029, logistic regression

### Lipaemia or TAG

tab Lipaemia

24,3% had lipaemia

#### Relation to mortality:

tab Lipaemia Mortality, row

logistic Mortality i.Lipaemia

Mortality with lipaemia is 62.2% and without it 47.8%, p=0,132

### Hyperkalemia

tab Hyperkalemia

24,3% had hyperkalemia

#### Relation to mortality:

tab Hyperkalemia Mortality, row

**logistic Mortality i.Hyperkalemia** **logistic Mortality i.Hyperkalemia**

logistic Mortality i.Hyperkalemia

Mortality with hyperkalemia is 70.3% and without í 45.2%, OR 2.9, p= 0,009, logistic regression

### Liver tests

tab Liver_tests

13.2% of patients had elevated liver function tests

#### Relation to mortality:

tab Liver_tests Mortality, row

logistic Mortality i.Liver_tests

There is no difference in mortality of patients with (50%) and without (52%) elevated LFTs, p=0.9.

### Rhabdo (myo, ^CK)

tab Rhabdo

There are 56% pacients with signs of rhabdomyolysis.

#### Relation to mortality:

tab Rhabdo Mortality, row

logistic Mortality i.Rhabdo

Mortality of pacients with ( 55%) and without (46%) rhabdomyolysisis not different, p=0,27

### AKI (anuria, oliguria, ^creatinine)

tab AKI

There are 39.5% of patients with AKI

#### Relation to mortality:

tab AKI Mortality, row

logistic Mortality i.AKI

There is no difference in mortality of patients with (51.7%) and without (51.1%) AKI, p=0.94

### Discoloration of urine

tab Discoloration_of_urine

10.5% had discoloration of urine.

#### Relation to mortality:

tab Discoloration_of_urine Mortality, row

logistic Mortality i.Discoloration_of_urine

Pacients with discoloration of urine had mortality of 68.8% as compared to 49.3% in the others, p=0.148.

### Hepatomegaly or fatty infiltration of liver

tab Hepatomegaly

There were 10.5% of patients withhepatomegaly or fatty infiltration of the liver.

#### Relation to mortality

tab Hepatomegaly Mortality, row

logistic Mortality i.Hepatomegaly

Patients with hepatomegaly or fatty infiltration of the liver had mortality of 81% and without it 48%, p= 0,019, logistic regression

### Pulmonary oedema

tab Pulmonary_oedema

There were only 4 (2,6%) patients with pulmonary oedema.

#### Relation to mortality:

tab Pulmonary_oedema Mortality, row

All 4 patients with pulmonary oedema died, as compared to 50% of patients without pulmonary oedema.

### Fever

tab Fever

19% of patients had fever.

#### Relation to mortality:

tab Fever Mortality, row

logistic Mortality i.Fever

Mortality of patients with fever is 86.2% as compared to 43% in patients without fever, p<0,001, logistic regression

### Hypotension

tab Hypotension

29.6% had hypotension

#### Relation to mortality:

tab Hypotension Mortality, row

logistic Mortality i.Hypotension

Hypotensive patients had 64% mortality as compared to 46% in those without hypotension, OR 2.1, p=0.037, logistic regression

### ECG changes

tab ECG

67.1% had Other ECG changes.

#### Relation to mortality:

tab ECG Mortality, row

logistic Mortality i.ECG

Mortality of patients with Other ECG changes was 61% as compared to 32% without them: OR 3.3, p<0.001

# Multivariant analysis

## Missing values analysis

**misstable summarize Mortality Age Sex Child Male Resp_infection TBI Status_epilepticus Nontrauma_neuro_dg Other Average_dose Duration Cum_dose Arrhythmia Cardiac_failure MAC Lipaemia Hyperkalemia Liver_tests Rhabdo AKI Discoloration_of_urine Hepatomegaly Pulmonary_oedema Fever Hypotension ECG Catecholamines**

misstable summarize Mortality Age Sex Child Male Resp_infection TBI Status_epilepticus Nontrauma_neuro_dg Other Average_dose Duration Cum_dose Arrhythmia Cardiac_failure MAC Lipaemia Hyperkalemia Liver_tests Rhabdo AKI Discoloration_of_urine Hepatomegaly Pulmonary_oedema Fever Hypotension ECG Catecholamines

Most missing values (24) is for average dose.

**misstable patterns Mortality Age Sex Male Resp_infection TBI Status_epilepticus Nontrauma_neuro_dg Other Average_dose Duration Arrhythmia Cardiac_failure MAC Lipaemia Hyperkalemia Liver_tests Rhabdo AKI Discoloration_of_urine Hepatomegaly Pulmonary_oedema Fever Hypotension ECG Catecholamines**

misstable patterns Mortality Age Sex Resp_infection TBI Status_epilepticus Nontrauma_neuro_dg Other Average_dose Duration Arrhythmia Cardiac_failure MAC Lipaemia Hyperkalemia Liver_tests Rhabdo AKI Discoloration_of_urine Hepatomegaly Pulmonary_oedema Fever Hypotension ECG Catecholamines

, frequency

There are 125 (82%) patients with complete data set, but sex can be ignored, giving 128 cases with complete analysable data

## Correlation analysis among variables

### Symptoms

pwcorr Arrhythmia Cardiac_failure MAC Lipaemia Hyperkalemia Liver_tests Rhabdo AKI Discoloration_of_urine Hepatomegaly Pulmonary_oedema Fever Hypotension ECG Catecholamines, sig print(10)

Table shows correlation coefficients and p values. Only values with p<0.1 are displayed.

And now only parametrs influencing mortality in univariate analysis.

pwcorr AgeOver55 Resp_infection TBI Other Average_dose_above5 Duration_3 Cum_dose_above360 Arrhythmia MAC Hyperkalemia Hepatomegaly Fever Hypotension ECG , sig print(10)

Of note are following correlations: ECG changes and Arrhythmia (R=0.87, p<0,001). Indeed Cumulative dose was calculated from Average dose (R=0,38, p<0,001) and Duration (R=0,47, p<0,001)

Hepatomegaly and Resp. infection, R= 0.59, p<0,001

Other and TBI, R=-0,4, p<0,001

TBI and Age over 55, R=-0,31, p<0,001

Average dose and Age over 55, R=-0,34, p<0,001

Cumulative dose and Age over 55, R=0,39, p<0,001

Arrhytmia and Age over 55, R=-0,39, p<0,001

Arrhytmia a ndAverage dose, R=0,31, p<0,001

ECG and Age over 55, R=-0,39, p<0,001

For further analysis – Average dose and Duration is used and Cumulative dose is withdrawn. Arrhytmia and other ECG changes represent each other – only other ECH changes are used in the analysis.

## Final model for logistic regression

logistic Mortality i.Average_dose_above5 i.Duration_3 i.TBI i.MAC i.Fever

coefplot, drop(_cons) xline(1) eform

coefplot, drop(_cons) xline(1) eform ciopts(recast(rcap))

estat class

lroc

lsens

estat ic

# Additional calculations

## Fever and Average infusion rate

tabstat Average_dose, by( Fever ) stat( N mean sd q min max)

graph box Average_dose , over(Fever) title("Fever")

regress Average_dose i.Fever, vce(robust)

Patients with fever recieved higher propofol infusion rate (by 1.72 mg/kg/h; p=0.004).
