# Supplementary figures and images for: Propofol infusion syndrome: a structured review of experimental studies and 153 published case reports
Source: Crit Care. 2015 Nov 12;19:398. doi: 10.1186/s13054-015-1112-5 (PMC4642662; doi:10.1186/s13054-015-1112-5)

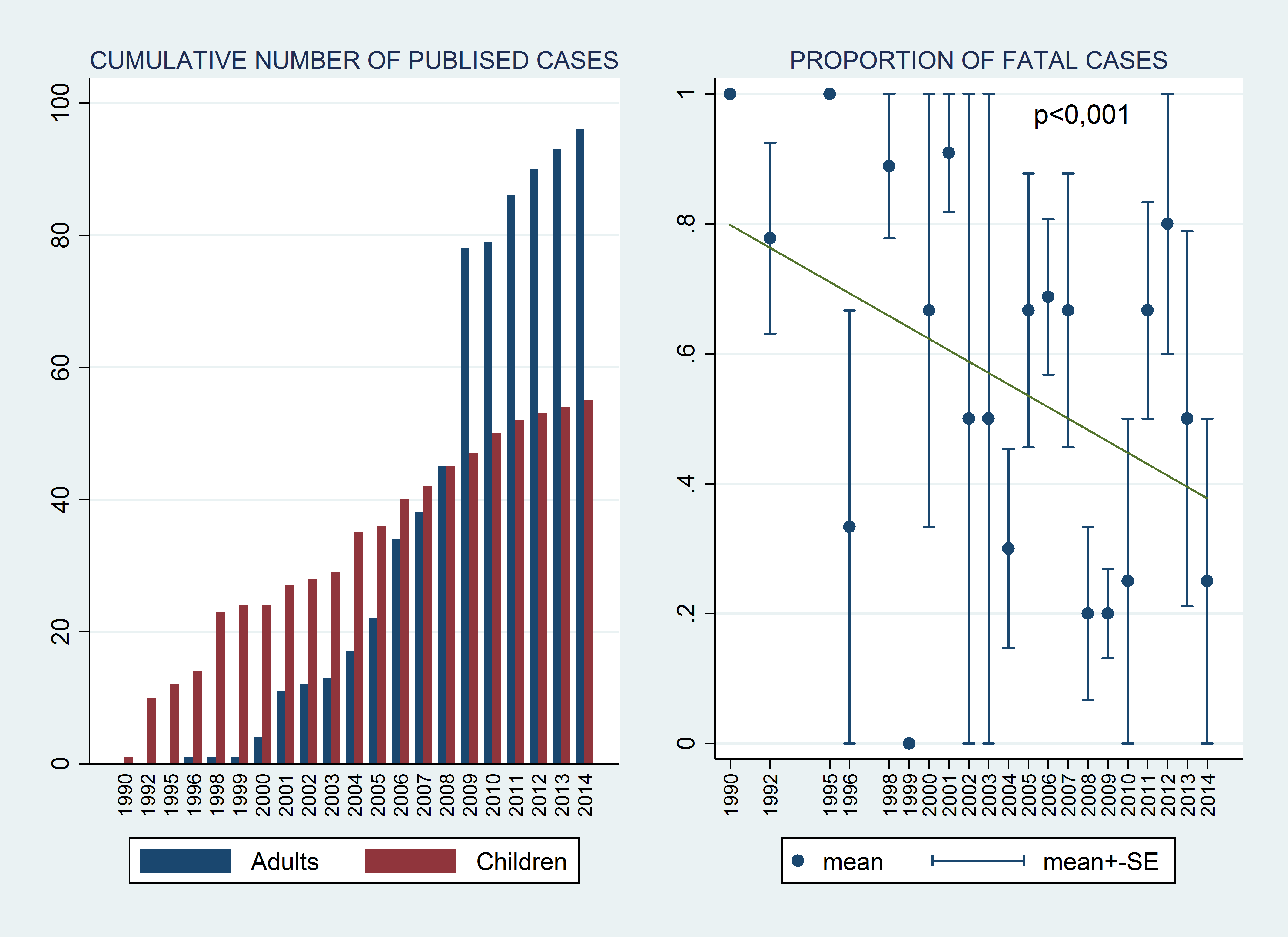

Supplement: Additional file 2: Figure S1. — Cumulative number of published cases of PRIS between 1990 and 2014 (left) and proportion of fatal cases published each year (right). Dashed line shows a trend determined by linear regression. (PNG 577 kb) [file 13054_2015_1112_MOESM2_ESM.png]

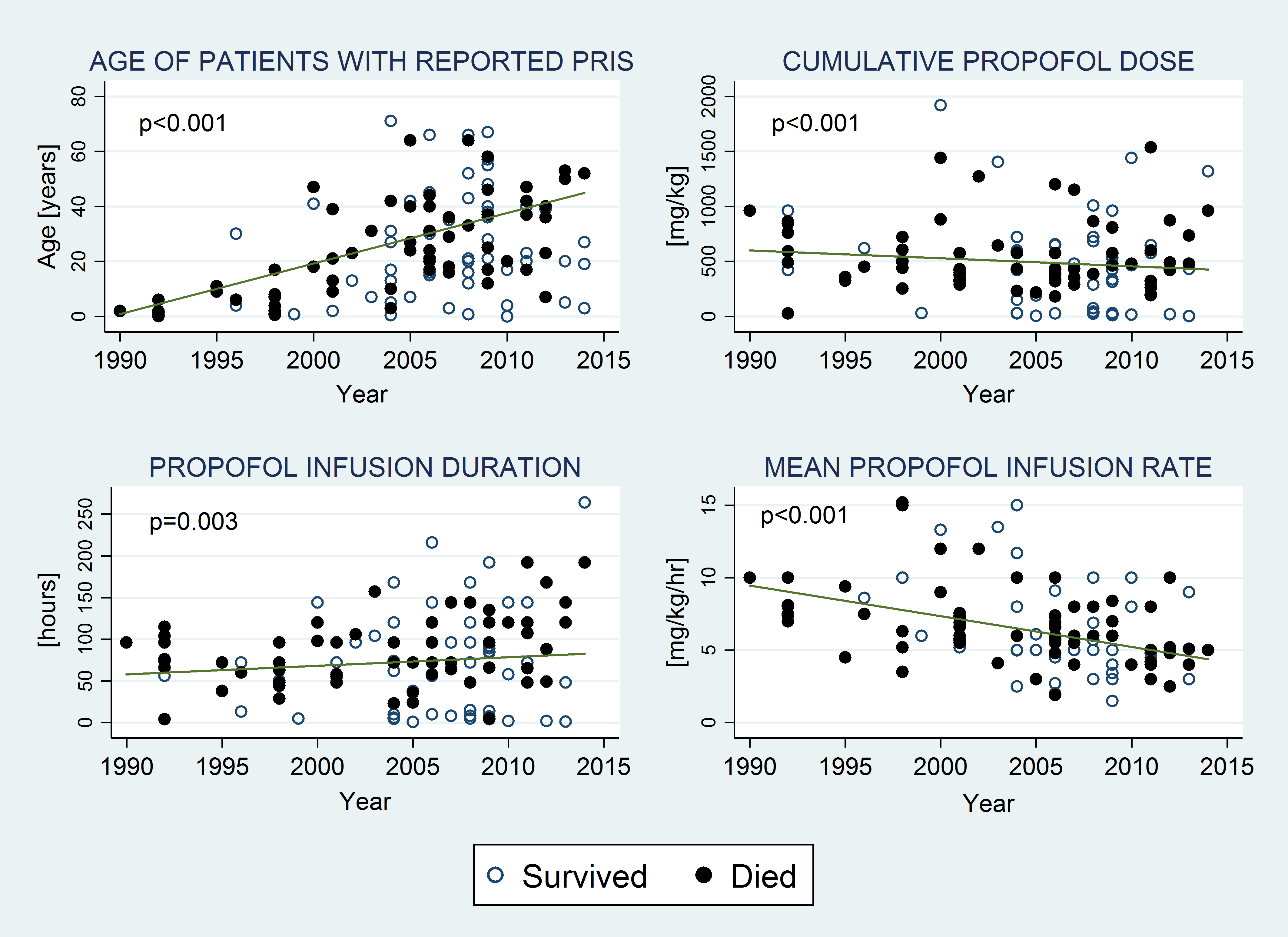

Supplement: Additional file 3: Figure S2. — The evolution of patients’ age, cumulative propofol dose, propofol infusion duration and mean propofol infusion rate leading to PRIS in published cases between 1990 and 2014. Each dot represents an individual case, the lines are trends determined by linear regression from all patients. (PNG 455 kb) [file 13054_2015_1112_MOESM3_ESM.png]

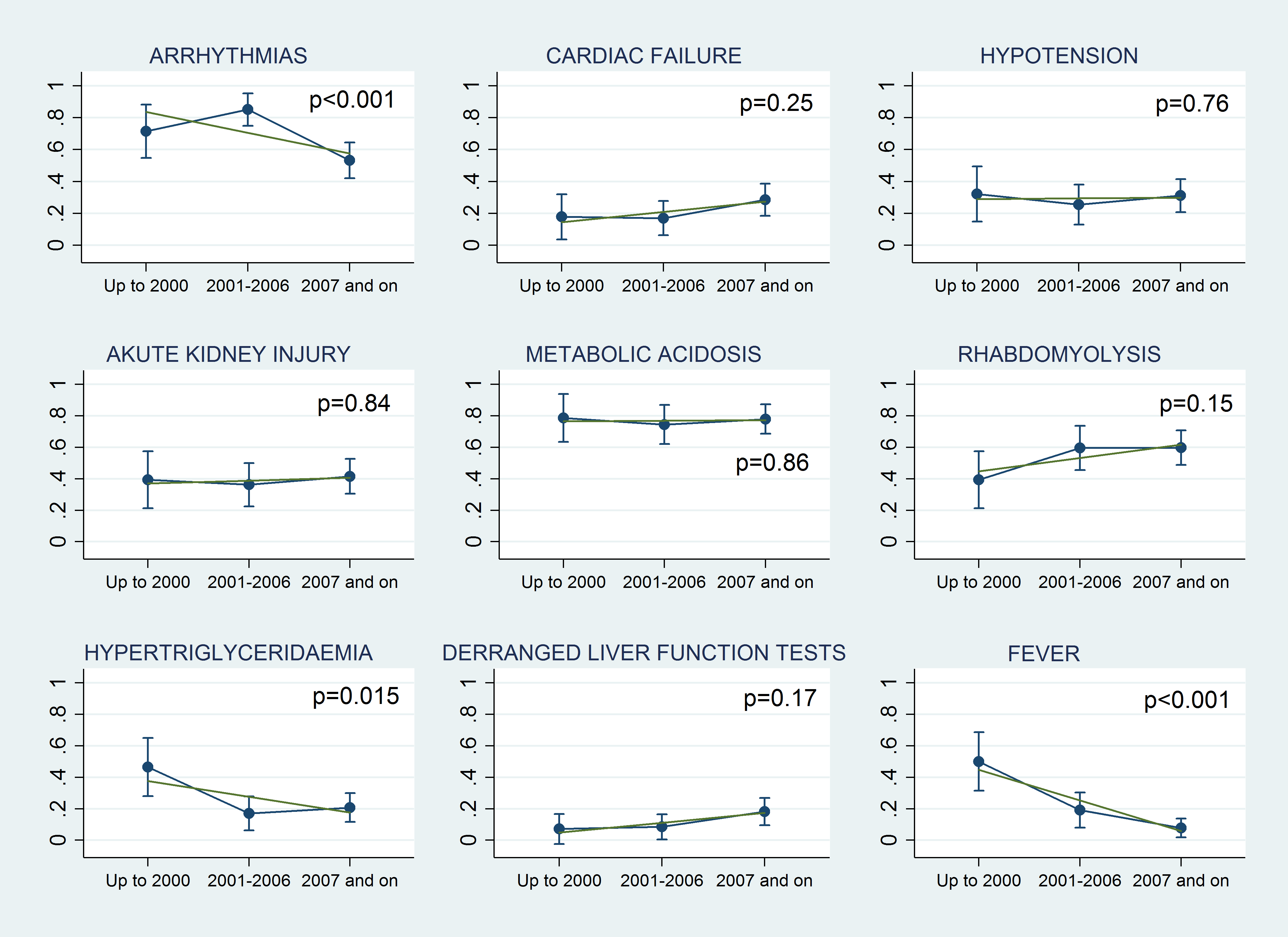

Supplement: Additional file 4: Figure S3. — Frequency of symptoms in reported cases of PRIS. Data presented as means, vertical bars are 95 % confidence intervals. (PNG 356 kb) [file 13054_2015_1112_MOESM4_ESM.png]

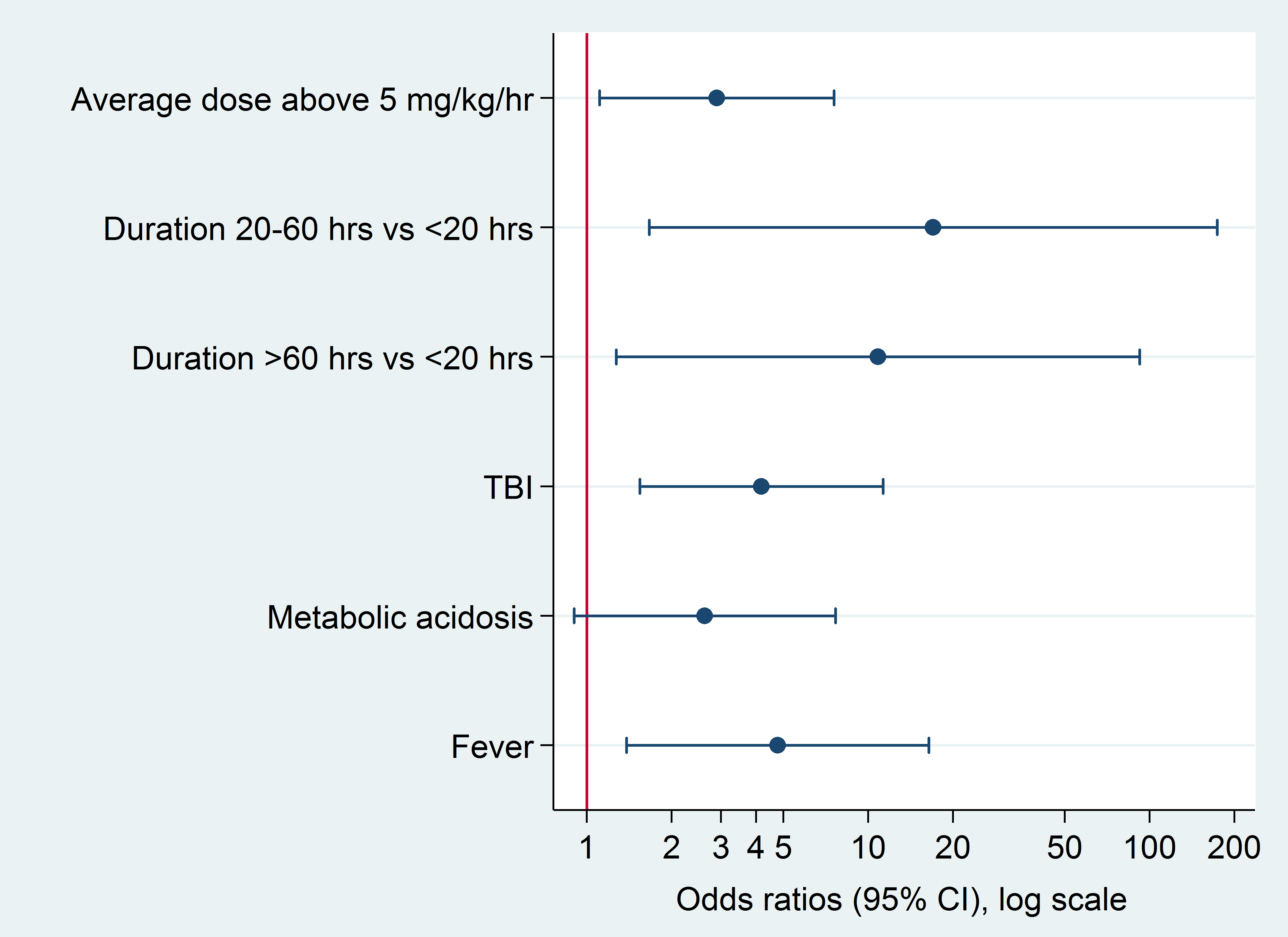

Supplement: Additional file 5: Figure S4A. — Adjusted odds ratio of factors influencing mortality of PRIS in multivariate model. (PNG 224 kb) [file 13054_2015_1112_MOESM5_ESM.png]

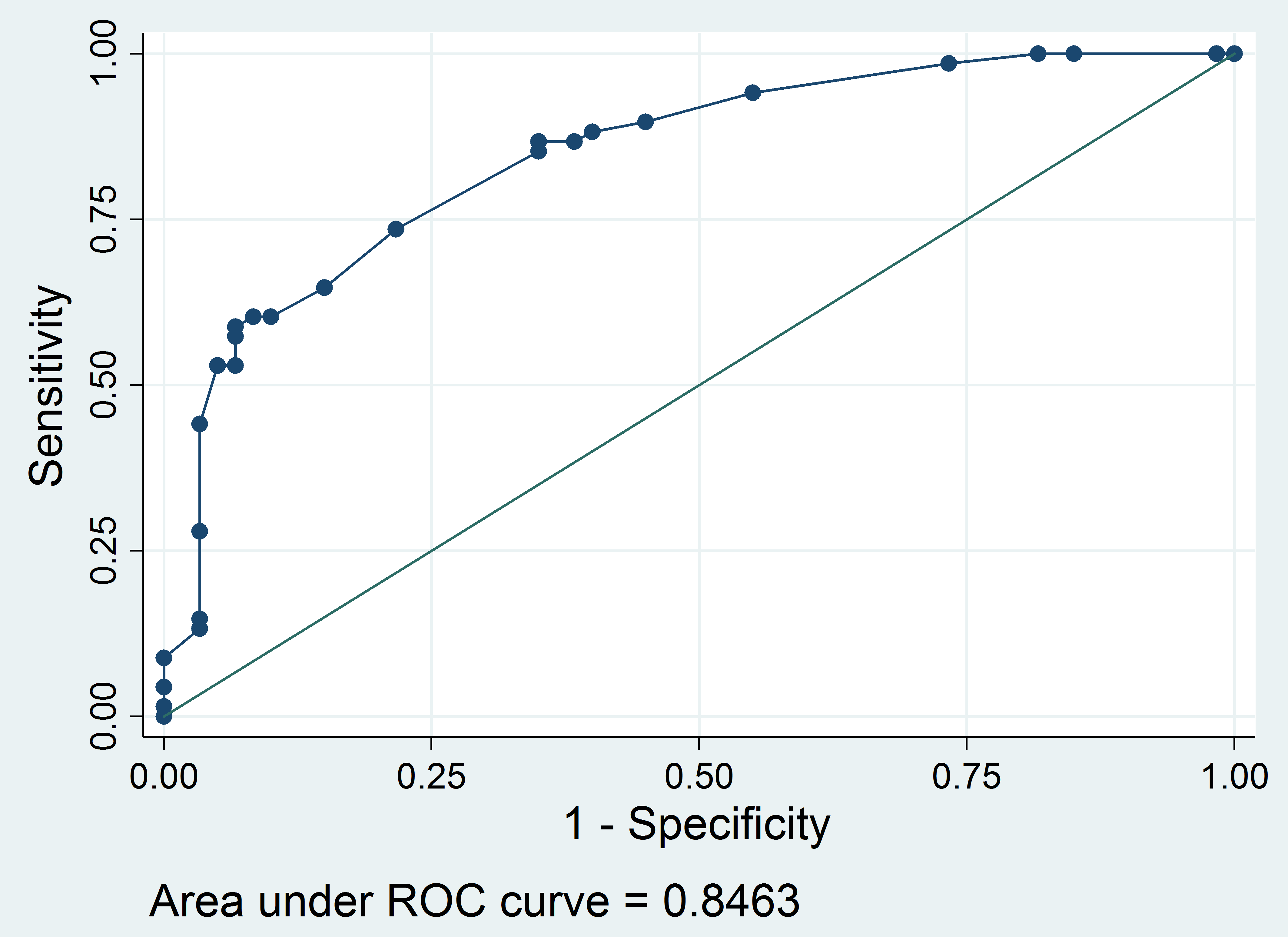

Supplement: Additional file 6: Figure S4B. — Receiver operator curve (ROC) of the final model. (PNG 304 kb) [file 13054_2015_1112_MOESM6_ESM.png]

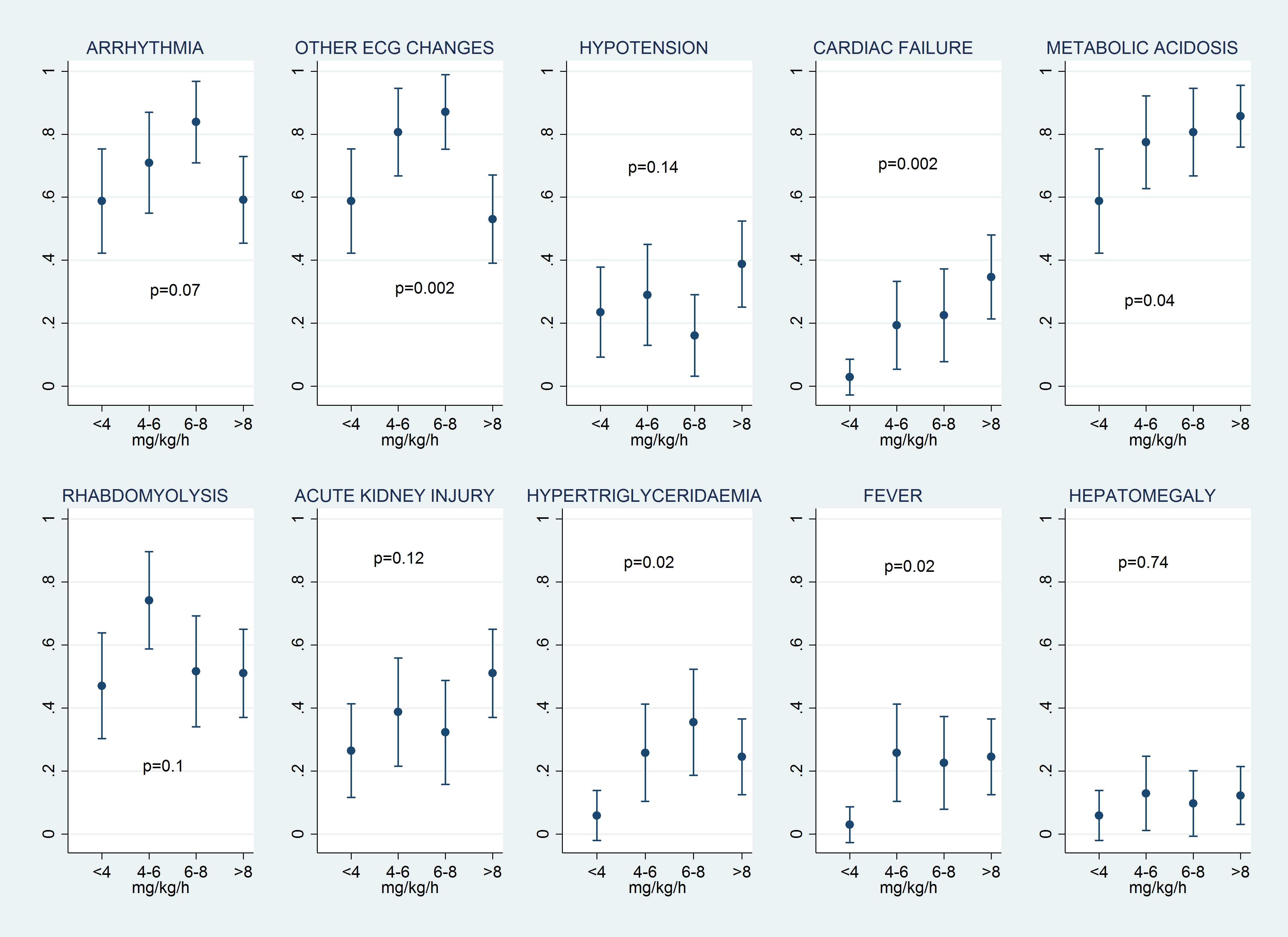

Supplement: Additional file 7: Figure S5. — Relative frequency (y axis) of symptoms of PRIS in patients divided into four quartiles of reported propofol infusion rate. Data presented as means, vertical bars are 95 % confidence interval. (PNG 375 kb) [file 13054_2015_1112_MOESM7_ESM.png]

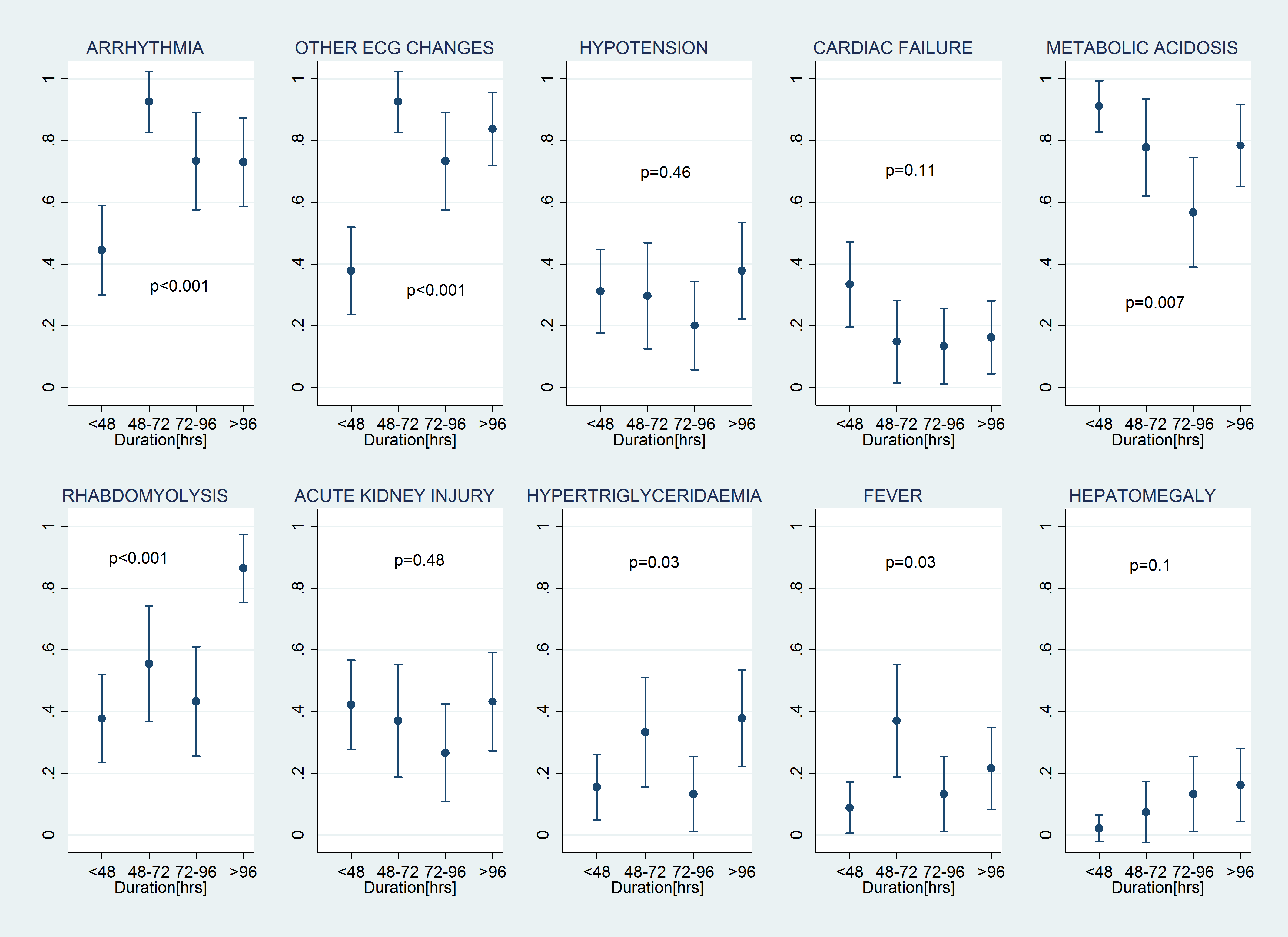

Supplement: Additional file 8: Figure S6. — Relative frequency (y axis) of symptoms of PRIS in patients divided into four quartiles of reported propofol infusion duration. Data presented as means, vertical bars are 95 % confidence intervals. (PNG 389 kb) [file 13054_2015_1112_MOESM8_ESM.png]
